# Pharmacologic Therapy of Diabetes and Overall Cancer Risk and Mortality: A Meta-Analysis of 265 Studies

**DOI:** 10.1038/srep10147

**Published:** 2015-06-15

**Authors:** Lang Wu, Jingjing Zhu, Larry J. Prokop, Mohammad Hassan Murad

**Affiliations:** 1 Center for Clinical and Translational Science, Mayo Clinic, Rochester, Minnesota; 2 Program of Quantitative Methods in Education, University of Minnesota, Minneapolis, Minnesota; 3 Mayo Clinic Libraries, Mayo Clinic, Rochester, Minnesota; 4 Division of Preventive Medicine, Mayo Clinic, Rochester, Minnesota; 5 Mayo Clinic Robert D. and Patricia E. Kern Center for the Science of Health Care Delivery, Mayo Clinic, Rochester, Minnesota

## Abstract

Different anti-diabetic medications (ADMs) may modify cancer risk and mortality in patients with diabetes. We conducted a systematic review and meta-analysis to estimate the magnitude of association and quality of supporting evidence for each ADM. A total of 265 studies (44 cohort studies, 39 case-control studies, and 182 randomized controlled trials (RCT)) were identified, involving approximately 7.6 million and 137,540 patients with diabetes for observational studies and RCTs, respectively. The risk of bias overall was moderate. Meta-analysis demonstrated that the use of metformin or thiazolidinediones was associated with a lower risk of cancer incidence (RR = 0.86, 95% CI 0.83-0.90, I^2^ = 88.61%; RR = 0.93, 95% CI 0.91-0.96, I^2^ = 0.00% respectively). On the other hand, insulin, sulfonylureas and alpha glucosidase inhibitor use was associated with an increased risk of cancer incidence (RR = 1.21, 95% CI 1.08-1.36, I^2^ = 96.31%; RR = 1.20, 95% CI 1.13-1.27, I^2^ = 95.02%; RR = 1.10, 95% CI 1.05-1.15, I^2^ = 0.00% respectively). Use of other types of ADMs was not significantly associated with cancer risk. This study indicates that some ADMs may modify the risk of cancer in individuals with diabetes. Knowledge of this risk may affect the choice of ADM in individuals concerned about cancer or at increased risk for cancer.

Diabetes is a prevalent disease associated with large global public health burden^1^,^2^. The age-standardized adult diabetes prevalence was nearly 10% in 2008^3^, and the prevalence is expected to be increased by 50% in the next two decades^4^. Numerous types of medications exist for controlling diabetes, including metformin, sulfonylureas, thiazolidinediones (TZD), insulin, dipeptidyl peptidase-4 (DPP-4) inhibitors, alpha-glucosidase inhibitors, glinides and glucagon-like peptide-1 (GLP-1) agonists^5^. Research has demonstrated that diabetes itself can increase the risk of developing cancer^6^,^7^,^8^, and different types of anti-diabetic medications (ADMs) can modify the risk of cancer in patients with diabetes^9^ Although the risk increase for an individual is small, this issue is critical due to the high prevalence of diabetes. A better understanding of which of the current medications affect cancer risk can guide clinical practice and impact patients’ decisions.

Several original studies and evidence summaries have evaluated at least one component or one drug relevant to this research question^10^,^11^,^12^,^13^,^14^,^15^,^16^,^17^,^18^,^19^,^20^,^21^,^22^,^23^,^24^,^25^,^26^,^27^,^28^,^29^,^30^,^31^,^32^,^33^,^34^,^35^. However, informed decision making requires a comprehensive summary of all available diabetes treatments that allows comparing the various options in terms of their effect on cancer risk and mortality. Considering different cancers can be associated with each other^36^,^37^ and individuals concerned about cancer may want to know the effects of ADMs on overall cancer risk, we thus conducted this systematic review and meta-analysis.

## Results

### Literature Search

**Figure 1:**
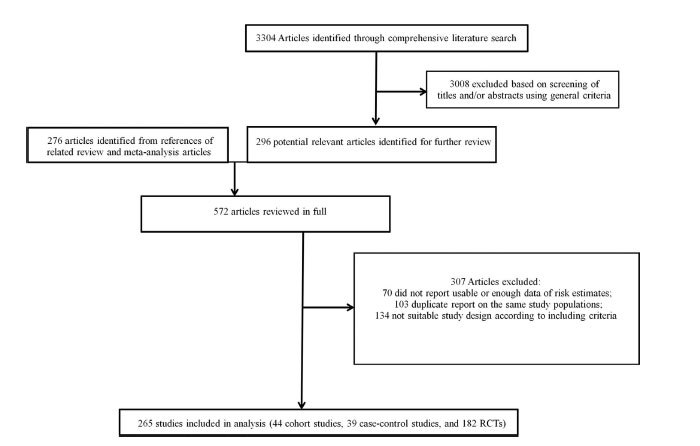
Flow chart for selection of eligible studies.

The detailed steps of the literature search are shown in Fig. 1. Overall, a total of 265 studies (44 cohort studies, 39 case-control studies, and 182 RCTs) met our inclusion criteria and were included in this review (see Table S1 and S2, and supplementary references). The detailed numbers of studies that evaluated cancer risk or mortality for each type of ADM are shown in supplementary material. Briefly, the three types of ADMs with largest number of studies are: 1) TZD: 120 studies for incidence (15 case-control studies, 12 cohort studies, 93 RCTs), 16 studies for mortality (all RCTs); 2) Insulin: 73 studies for incidence (34 case-control studies, 26 cohort studies, 13 RCTs), 12 studies for mortality (10 cohort studies and 2 RCTs); and 3) sulfonylureas: 72 studies for incidence (18 case-control studies, 16 cohort studies, 38 RCTs), 12 studies for mortality (4 cohort studies and 8 RCTs). Among the 85 studies that evaluated insulin, 59 focused on type 2 diabetes whereas the remaining studies did not specify diabetes type.

### Study Characteristics

The detailed characteristics of the included studies are shown in Table S1 and Table S2. For cohort studies, 18 studies were conducted in Europe, 16 in America, 9 in Asia, 1 for international. For case-control studies, 11 studies were conducted in Europe, 19 in America, 7 in Asia, 2 for international. For RCTs, 33 studies were conducted in Europe, 51 in America, 25 in Asia, 1 in Africa, 66 for international, and 7 for unknown. Overall the included observational studies enrolled approximately 7.6 million patients with diabetes and had a median follow up of about 5 years (range 1–34 years), and RCTs enrolled 137,540 patients with diabetes and had a median follow up of 0.5 year (range 8 weeks-8.7 years).

The overall NOS quality scores for observational studies are listed in Table S1. Overall, the studies had fair methodological quality: 51 studies were categorized with low risk of bias, 31 with moderate risk, and 1 with high risk. For the extracted estimations, 36 of the overall 39 case-control studies and 33 of the overall 44 cohort studies were adjusted for important covariates. The overall risks of bias for RCTs are listed in Table S2. Among the 182 RCTs, only 36 were determined as low risk, and other 146 trials had high or unreported risks in at least one of the 3 components assessed and were thus categorized as high risk.

### Meta-analysis

The summary risk estimations between each specific type of ADMs and cancer incidence and mortality are shown in Table 1 and 2 respectively.

Insulin use was significantly associated with an increased risk of cancer (73 studies; RR = 1.21, 95% CI 1.08-1.36) (Table 1a). There was considerable heterogeneity between studies (I^2^ = 96.31%), which could potentially be partially explained by different study designs. There was no indication of significant publication bias (p = 0.15). In subgroup analyses, the association was detected in studies adjusting for covariates, observational studies, and studies with low risk of bias. With regard to cancer mortality, we did not detect significant association with insulin use (12 studies; RR = 1.19, 95% CI 0.80–1.77) (Table 2a). The heterogeneity between studies was also considerable (I^2 ^= 98.19%), and it was suggested to be explained by estimate adjustments. Publication bias existed as indicated by p = 0.02 of Egger’s test. The subgroup analyses suggested an increased risk in studies adjusting for covariates and studies with low risk of bias.

Meta-analysis of 66 and 12 studies respectively, demonstrated significant association between the use of metformin and the risk of cancer (RR = 0.86, 95% CI 0.83-0.90) and cancer mortality (RR = 0.70, 95% CI 0.53-0.94) (Table 1b and 2b). There were considerable heterogeneity between studies for both cancer incidence (I^2^ = 88.61%) and mortality (I^2^ = 54.53). Study design and estimate adjustments potentially explain the heterogeneity of cancer incidence but neither factor explains heterogeneity for cancer mortality. And publication bias was likely for cancer incidence (p = 0.01), but not for cancer mortality (p = 0.96). In almost all strata of subgroup analyses except for RCTs, such an inverse association was detected.

Use of sulfonylureas was associated with an increased risk of cancer (72 studies, RR = 1.20, 95% CI 1.13-1.27) (Table 1c). The heterogeneity existed with I^2^ = 95.02%, and similar with the case of insulin, this heterogeneity can potentially be explained by different study design. No significant publication bias was detected (p = 0.06). In the subgroup analyses, such an association was detected in strata of studies adjusting for covariates, observational studies, and studies with low risk of bias. On the other hand, we did not detect significant association between sulfonylureas use and cancer mortality (12 studies, RR = 1.08, 95% CI 0.99-1.18) (Table 2c). No substantial heterogeneity existed between studies (I^2^ = 0.00%), and the test for publication bias was nonsignificant (p = 0.67). No significant association was detected in subgroup analyses as well.

Similar to the case of metformin, TZD use was associated with a decreased risk of cancer (119 studies, RR = 0.93, 95% CI 0.91-0.96) (Table 1d). There was no considerable heterogeneity between studies (I^2^ = 0.00%). The test for publication bias was nonsignificant (p = 0.37). With regard to cancer mortality, we did not detect significant association (16 studies, RR = 1.40, 95% CI 0.57-3.40) (Table 2d). Also there was no substantial heterogeneity between studies (I^2^ = 0.00%), although the publication bias was likely (p = 0.01).

Meta-analysis of 62 studies showed that DPP-4 inhibitors were not associated with the risk of cancer (RR = 0.92, 95% CI 0.82-1.04) (Table 1e). There was no heterogeneity between studies (I^2^ = 0.00%), and the test for publication bias was nonsignificant (p = 0.87). The nonsignificant finding was confirmed by subgroup analyses. With regard to cancer mortality, there was only one study which indicated no significant association (RR = 0.17, 95% CI 0.01–4.18) (Table 2e).

Alpha glucosidase inhibitor use was associated with increased risk of cancer (13 studies, RR =  1.10, 95% CI 1.05-1.15) (Table 2f). No substantial heterogeneity between studies existed (I^2^ = 0.00%), and the test for publication bias was nonsignificant (p = 0.50). The significant association was also detected in subgroup of studies adjusting for covariates. On the other hand, there were only two studies with estimations for cancer mortality, and the overall association was not significant (RR = 1.40, 95% CI 0.09-21.94) (Table 2f).

Meta-analysis of 8 studies did not demonstrate a significant association between glinides and risk of cancer (RR = 1.06 and 95% CI 0.83–1.37) (Table 1g), which was confirmed by subgroup analyses. There was no considerable heterogeneity between studies (I^2^ = 25.00%), and publication bias was likely (p = 0.01). For GLP-1 agonist use, meta-analysis of 16 studies demonstrated no significant association with cancer risk (RR = 1.12, 95% CI 0.61–2.06) (Table 1h). There was no heterogeneity between studies (I^2^ = 0.00%), and the test for publication bias was nonsignificant (p = 0.48). The associations in subgroup analyses were not significant as well.

Dapagliflozin was not associated with the risk of cancer (7 studies, RR = 0.90, 95% CI 0.49–1.65) (Table 1i). There was no heterogeneity between studies (I^2 ^= 0.00%), the test for publication bias was nonsignificant (p = 0.15).

## Discussion

We updated the existing evidence base and presented a comprehensive summary of the association between cancer risk and mortality and the various available ADMs.

We found that, relative to non-use, metformin was associated with a 14% and 30% reduction in risk of cancer incidence and mortality, respectively. On the other hand, use of insulin, sulfonylureas or alpha glucosidase inhibitor was associated with a 21%, 20%, and 10% increase in the risk of cancer incidence, respectively, but no association with cancer mortality. TZD use was associated with a 7% decrease in risk of cancer incidence but no association with cancer mortality. For other types of ADMs, including DPP-4 inhibitor, glinides, GLP-1 agonist and Dapagliflozin, no significant association with cancer incidence or mortality was found. The associations from subgroup analyses were not always consistent with the overall results, especially for the analysis based on gender, while the power issue needs to be considered when interpreting these results since in certain strata only limited number of studies is available.

Preclinical studies have suggested an anti-tumor effect of metformin, mediating by inhibition of the mammalian target of rapamycin pathway, which is known to be an effector of growth factor signaling activated in malignant cells, as well as activation of adenosine monophosphate-activated protein kinase, an energy sensor that regulates a variety of cell function^38^. Besides, it may inhibit cyclin D1 expression and Rb phosphorylation, which further inhibit cell growth and promote senescence^39^. Although the doses used in these preclinical studies are usually higher than those used in clinical practice, these experiments provided a mechanistic rationale of the anti-tumor effect of metformin. Our results are largely consistent with previous meta-analyses with similar context^21^,^23^,^34^,^35^, supporting an overall reducing effect of metformin on cancer risk. On the other hand, there is considerable heterogeneity between studies, which partially could be contributed to different study designs. In a subgroup analysis the risk reduction effect of metformin was demonstrated in observational studies while not in RCTs, which confirms a previous meta-analysis^24^. However, we should acknowledge that the average follow up time in observational studies is much longer than that in RCTs, which could potentially explain this finding.

TZDs were also demonstrated to have antitumor capacities through preclinical studies. It is shown to induce cell apoptosis by increasing p53 and reducing Bcl-2^40^. Besides, it could induce cell growth arrest, prevent cell differentiation and cancer invasion through inhibition of the ubiquitin-proteasome system and the extracellular signal-regulated kinase pathway^40^,^41^. In our study we demonstrated a cancer risk reduction effect of TZDs, which is consistent with previous meta-analyses^27^,^31^, although its protective effect was not supported by subgroup of RCTs. However, we should note that use of one specific kind of TZDs, pioglitazone, is associated with increased risk of bladder cancer, as previously demonstrated^13^. Individuals with diabetes, especially for those with a family history of bladder cancer, may want to consider this in their medication decision.

Insulin is a growth factor which could stimulate neoplastic growth^42^,^43^. It is shown to promote carcinogenesis by increasing insulin-like growth factor-1 activity, stimulating multiple signaling cascades, enhancing cell proliferation, and affecting metabolism^44^. In this meta-analysis, we conclude that insulin use is associated with a significantly increased risk of cancer, which is consistent with conclusions of prior studies^25^,^26^,^29^,^33^ and brings the evidence base up to date providing a more precise estimation. However, it is worth noting that this association was not detected in RCTs, which suggests that insulin may not increase cancer risk at least in the short term. Further studies are warranted to clarify this question. Similarly, sulfonylureas could increase insulin secretion and exerts similar effect. Our analysis demonstrates an association of sulfonylureas with cancer risk, which adds additional studies to a previous meta-analysis^35^ and updates the estimate of association.

This systematic review and meta-analysis has several strengths. We conducted a comprehensive search involving 6 electronic databases without language restriction, adding more studies to some of the previous systematic reviews to produce more precise estimates. We also evaluated most of the available ADMs.

Several limitations of this study should be noted. Firstly, the dose-response relationship could not be assessed in this analysis, which is due to data unavailability of most of included studies. Secondly, based on subgroup analyses of study design, those significant associations between ADMs and cancer risk detected in our study mainly come from observational studies. The largest limitation of observational studies is the lack of experimental random allocation to the intervention, which decreases the validity of the findings. For example, it was well established that several time-related biases, including immortal time bias, time-window bias, and bias from time lag and latency, might significantly mask the real effects^45^. Without individual data with time varying drug exposure, these biases could not be sufficiently corrected. On the other hand, for most RCTs, the average follow up time is much shorter than that of observational studies, making them less appropriate to detect an association with cancer, an outcome that requires longer exposure time. Thirdly, it is worth noting that for the majority of included studies, the comparison group for each evaluated ADM was with other ADM(s), which were demonstrated to inherently affect risk of cancer as well. Therefore the pooled risk estimates for each ADM might be confounded.

Although we demonstrated that some ADMs modify the risk of cancer, the magnitude of association in absolute terms is not large. This absolute risk however is dependent on the baseline risk of an individual. Therefore, in patients with increased cancer risk due to family history or smoking, or those particularly concerned about cancer, we provide the information needed for shared decision making. It is possible that for some poorly controlled patients, the cancer risk is less important; whereas for others it is important. Patients’ values and preferences along with the best available evidence are needed for decision making.

In conclusion, our study demonstrated that some ADMs may modify the risk of cancer in individuals with diabetes. Knowledge of this risk may affect the choice of ADM in individuals concerned about cancer or at increased risk for cancer.

## Methods

We developed a study protocol that defined inclusion and exclusion criteria, search strategy, outcomes and analysis methods. This systematic review is conducted following guidance provided by the Cochrane Handbook and is reported according to the Preferred Reporting Items for Systematic reviews and Meta-Analyses guidelines^46^.

### Data Sources and Search Strategies

A comprehensive search of Ovid Medline In-Process & Other Non-Indexed Citations, Ovid MEDLINE, Ovid EMBASE, Ovid Cochrane Central Register of Controlled Trials, Ovid Cochrane Database of Systematic Reviews, and Scopus was conducted from each database’s earliest inception to March 2014. There were no search restrictions based on language or type of population. The search strategy was designed and conducted by an experienced librarian with input from the study’s principle investigator. Controlled vocabulary supplemented with keywords was used to search for studies of the risk of cancer from ADMs. The detailed strategy is within the online supplementary material. We also reviewed references of over fifty related review articles and meta-analyses to identify additional potential studies.

### Study Selection

Studies were eligible if they (i) were cohort studies, case–control studies, or RCTs; (ii) clearly defined and evaluated exposure to ADMs; (iii) reported incidence or mortality of cancer in patients with diabetes; (iv) reported relative risk (RR), hazard ratio (HR), odds ratio (OR), or sufficient data for calculation. Studies were included regardless of publication status, sample size, length of follow-up, or language of publication. If multiple publications from the same study were identified, we included the study with the largest number of cases and most applicable information^47^,^48^.

### Data Extraction and Quality Assessment

A pair of investigators independently carried out the abstract screening, full text screening, data abstraction, and quality assessment. Disagreements were resolved by consensus, with input from the senior investigator. Data abstracted from each study included authors’ name, year of publication, study region, characteristics of study population, sample size, age, length of follow-up, types of ADMs, RR or HR or OR and the 95% confidence interval (CI), matched or adjusted confounding variables, risk of bias indicators and outcomes of interest. We focused on risk estimation of using one specific type of ADM vs no treatment of the particular medication of interest in our study. If multiple estimates of the association for the same outcome were reported, we abstracted the estimate that was most appropriately adjusted. If no adjusted estimates were presented, we used the crude estimate^49^. When the eligible studies did not present enough data or important information, corresponding and/or first authors were contacted.

To assess the study quality, we used the Newcastle-Ottawa Quality Assessment Scale (NOS)^50^,^51^ for observational studies in terms of population and sample methods, exposure and outcome descriptions, and statistical matching/adjustments of the data. A score between 7–9 represents low risk of bias, 4–6 represents moderate risk of bias, and 0–3 represents high risk of bias. The quality of RCTs was assessed using a revised form of Cochrane Collaboration’s tool for assessing risk of bias in randomized trials^52^ focusing on the adequacy of randomization, allocation concealment procedures and blinding. If any one of these three components was judged as high or unclear risk, the trial was categorized as high risk trial; otherwise the trial was determined as low risk trial.

### Statistical Methods

We extracted or calculated RR (or HR or OR) and related 95% CI from each included studies. Due to the rarity of cancer in general population, RR, HR and OR were deemed equivalent. We then pooled the log transformed RR using the DerSimonian & Laird random effects method with the estimate of heterogeneity from the Mantel-Haenszel model^53^. We conducted subgroup analyses based on study design, whether estimates were adjusted and based on the gender of participants. We conducted sensitivity analysis in which we only included studies with low risk of bias. We used I^2^ to assess the heterogeneity across the include studies, where I^2>^50% suggests high heterogeneity^48^,^54^. Publication bias was evaluated via Egger’s linear regression test^55^. A P value of less than 0.05 was considered representative of significant publication bias. All statistical analyses were conducted using Comprehensive Meta-analysis Version 2, Biostat, Englewood NJ (2005).

## Additional Information

**How to cite this article**: Wu, L. *et al.* Pharmacologic therapy of diabetes and overall cancer risk and mortality: A Meta-analysis of 265 studies. *Sci. Rep.* **5**, 10147; doi: 10.1038/srep10147 (2015).

## Supplementary Material

Supplementary Information

Supplementary Tables

## Figures and Tables

**Table 1:** (a-i) Summary and subgroup analyses of the association between anti-diabetic medications and cancer incidence.

**Anti-diabetic medication kind**	**Number of studies**	**RR**	**95% CI lower boundary**	**95% CI higher boundary**	**P value for effect**	**P value for Egger’s test**	**I**^**2**^ **(%)**	**P value for difference between subgroups**
(a) Insulin								
All studies	73	1.21	1.08	1.36	0.00	0.15	96.31	—
Estimates adjusted?								
No	22	1.02	0.80	1.30	0.85	—	47.01	0.15
Yes	51	1.25	1.10	1.42	0.00	—	97.34	
Study design								
Case-control	34	1.40	1.12	1.75	0.00	—	97.27	0.03
Cohort	26	1.11	1.04	1.20	0.00	—	82.97	
RCT	13	1.00	0.88	1.13	0.96	—	0.00	
Studies with low risk of bias	39	1.20	1.03	1.39	0.02	—	98	
Gender								
Female	16	1.06	0.86	1.31	0.59	—	76	
Male	16	0.92	0.79	1.09	0.33	—	80	
(b) Metformin								
All studies	66	0.86	0.83	0.90	0.00	0.01	88.61	—
Estimates adjusted?								
No	29	0.77	0.70	0.85	0.00	—	0.00	0.01
Yes	37	0.88	0.84	0.92	0.00	—	93.04	
Study design								
Case-control	22	0.71	0.63	0.80	0.00	—	83.33	0.00
Cohort	21	0.88	0.83	0.92	0.00	—	95.18	
RCT	23	1.05	0.94	1.18	0.36	—	0.00	
Studies with low risk of bias	29	0.89	0.85	0.93	0.00	—	94	
Gender								
Female	13	0.76	0.69	0.84	0.00	—	0	
Male	12	0.82	0.70	0.97	0.02	—	63	
(c) Sulfonylureas								
All studies	72	1.20	1.13	1.27	0.00	0.06	95.02	—
Estimates adjusted?								
No	42	1.30	1.18	1.43	0.00	—	0.00	0.15
Yes	30	1.19	1.12	1.27	0.00	—	97.88	
Study design								
Case-control	18	1.52	1.16	1.98	0.00	—	97.83	0.00
Cohort	16	1.11	1.04	1.18	0.00	—	97.45	
RCT	38	0.91	0.81	1.02	0.12	—	0.00	
Studies with low risk of bias	32	1.17	1.10	1.24	0.00	—	98	
Gender								
Female	16	1.83	0.61	5.55	0.28	—	95	
Male	18	1.00	0.81	1.23	0.99	—	62	
(d) TZDs								
All studies	119	0.93	0.91	0.96	0.00	0.37	0.00	—
Estimates adjusted?								
No	97	0.92	0.83	1.02	0.10	—	0.00	0.86
Yes	22	0.91	0.82	1.01	0.06	—	68.77	
Study design								
Case-control	15	0.98	0.90	1.08	0.73	—	41.32	0.10
Cohort	12	0.80	0.68	0.95	0.01	—	65.74	
RCT	92	0.96	0.86	1.08	0.48	—	0.00	
Studies with low risk of bias	25	0.93	0.85	1.01	0.07	—	60	
Gender								
Female	35	0.76	0.57	1.01	0.06	—	0	
Male	35	0.88	0.74	1.05	0.16	—	0	
(e) DPP-4 inhibitor								
All studies	62	0.92	0.82	1.04	0.17	0.87	0.00	—
Estimates adjusted?								
No	60	0.93	0.82	1.05	0.22	—	0.00	0.38
Yes	2	0.64	0.28	1.47	0.29	—	0.00	
Study design								
Case-control	1	0.52	0.13	2.13	0.36	—	0.00	0.54
Cohort	2	0.78	0.50	1.22	0.28	—	0.00	
RCT	59	0.94	0.83	1.06	0.30	—	0.00	
Studies with low risk of bias	24	0.95	0.83	1.08	0.42	—	0	
Gender								
Female	8	0.52	0.19	1.40	0.20	—	0	
Male	7	0.64	0.15	2.74	0.54	—	0	
(f) alpha glucosidase inhibitor								
All studies	13	1.10	1.05	1.15	0.00	0.50	0.00	—
Estimates adjusted?								
No	6	0.89	0.63	1.26	0.52	—	0.00	0.24
Yes	7	1.10	1.05	1.15	0.00	—	0.00	
Study design								
Case-control	6	1.12	0.91	1.38	0.30	—	15.23	0.52
Cohort	2	0.93	0.68	1.27	0.64	—	0.00	
RCT	5	0.69	0.17	2.73	0.59	—	0.00	
Studies with low risk of bias	6	1.10	1.05	1.15	0.00	—	0	
Gender								
Female	4	1.04	0.64	1.67	0.88	—	0	
Male	4	1.02	0.52	2.01	0.95	—	0	
(g) Glinides								
All studies	8	1.06	0.83	1.37	0.62	0.01	25.00	—
Estimates adjusted?								
No	4	0.77	0.48	1.23	0.28	—	0.00	0.10
Yes	4	1.20	0.94	1.53	0.14	—	21.79	
Study design								
Case-control	3	0.99	0.50	1.93	0.97	—	45.44	0.82
Cohort	3	0.96	0.68	1.35	0.81	—	0.00	
RCT	2	0.50	0.07	3.76	0.50	—	0.00	
Studies with low risk of bias	3	1.15	0.83	1.60	0.41	—	48	
Gender								
Female	1	0.77	0.35	1.70	0.52	—	—	
Male	2	0.81	0.44	1.48	0.49	—	—	
(h) GLP-1 agonist								
All studies	16	1.12	0.61	2.06	0.72	0.48	0.00	—
Estimates adjusted?								
No	14	1.04	0.45	2.39	0.93	—	0.00	0.80
Yes	2	1.22	0.50	2.98	0.67	—	0.00	
Study design								
Cohort	2	1.22	0.50	2.98	0.67	—	0.00	0.80
RCT	14	1.04	0.45	2.39	0.93	—	0.00	
Studies with low risk of bias	5	1.26	0.58	2.73	0.56	—	0	
Gender								
Female	5	0.92	0.16	5.37	0.92	—	0	
Male	5	0.78	0.14	4.53	0.79	—	0	
(i) Dapagliflozin								
All studies	7	0.90	0.49	1.65	0.73	0.15	0.00	—
Studies with low risk of bias	5	0.88	0.46	1.68	0.70	—	0	

RR: Relative Risk.

**Table 2:** (a–f) Summary and subgroup analyses of the association between anti-diabetic medications and cancer mortality.

**Anti-diabetic medication kind**	**Number of studies**	**RR**	**95% CI lower boundary**	**95% CI higher boundary**	**P value for effect**	**P value for Egger’s test**	**I**^**2**^ **(%)**	**P value for difference between subgroups**
(a) Insulin								
All studies	12	1.19	0.80	1.77	0.40	0.02	98.19	—
Estimates adjusted?								
No	2	0.47	0.41	0.55	0.00	—	87.05	0.00
Yes	10	1.49	1.07	2.05	0.02	—	90.17	
Study design								
Cohort	10	1.16	0.75	1.82	0.50	—	98.46	0.85
RCT	2	1.27	0.60	2.66	0.53	—	72.90	
Studies with low risk of bias	8	1.53	1.08	2.18	0.02	—	92	
Gender								
Female	1	0.51	0.47	0.55	0.00	—	—	
Male	1	0.44	0.41	0.47	0.00	—	—	
(b) Metformin								
All studies	12	0.70	0.53	0.94	0.02	0.96	54.53	—
Estimates adjusted?								
No	5	1.27	0.59	2.70	0.54	—	23.75	
Yes	7	0.62	0.46	0.85	0.00	—	62.13	
Study design								
Cohort	6	0.66	0.49	0.89	0.01	—	61.26	0.51
RCT	6	0.91	0.37	2.23	0.83	—	52.60	
Studies with low risk of bias	5	0.69	0.52	0.91	0.01	—	60	
Gender								
Female	2	0.83	0.06	12.29	0.89	—	—	
Male	2	0.71	0.05	9.48	0.80	—	—	
(c) Sulfonylureas								
All studies	12	1.08	0.99	1.18	0.07	0.67	0.00	—
Estimates adjusted?								
No	7	1.18	0.28	5.06	0.82	—	0.00	0.82
Yes	5	1.00	0.73	1.35	0.98	—	58.82	
Study design								
Cohort	4	1.04	0.74	1.44	0.83	—	64.91	0.50
RCT	8	0.78	0.37	1.65	0.51	—	0.00	
Studies with low risk of bias	5	1.04	0.76	1.41	0.82	—	53	
Gender								
Female	5	1.60	0.28	9.12	0.59	—	0	
Male	5	1.67	0.29	9.61	0.56	—	0	
(d) TZDs								
All studies	16	1.40	0.57	3.40	0.46	0.01	0.00	—
Studies with low risk of bias	1	0.99	0.02	49.53	1.00	—	—	
Gender								
Female	11	1.13	0.35	3.63	0.84	—	0	
Male	10	1.12	0.33	3.84	0.85	—	0	
(e) DPP-4 inhibitor								
All studies	1	0.17	0.01	4.18	0.28	—	—	—
(f) alpha glucosidase inhibitor								
All studies	2	1.40	0.09	21.94	0.81	—	—	—
Gender								
Female	2	1.34	0.09	20.50	0.83	—	—	
Male	2	1.41	0.09	22.08	0.81	—	—	

RR: Relative Risk.
