# Supplementary material for: Pharmacologic Therapy of Diabetes and Overall Cancer Risk and Mortality: A Meta-Analysis of 265 Studies
Source: Sci Rep. 2015 Jun 15;5:10147. doi: 10.1038/srep10147 (PMC4467243; doi:10.1038/srep10147)

Subject Area: Public Health

Pharmacologic therapy of diabetes and overall cancer risk and mortality: A Meta-analysis of 265 studies

Lang Wu[1], Jingjing Zhu[2], Larry J. Prokop[3], Mohammad Hassan Murad[4,5]

1 Center for Clinical and Translational Science, Mayo Clinic, Rochester, Minnesota;

2 Program of Quantitative Methods in Education, University of Minnesota, Minneapolis, Minnesota;

3 Mayo Clinic Libraries, Mayo Clinic, Rochester, Minnesota;

4 Division of Preventive Medicine, Mayo Clinic, Rochester, Minnesota;

5 Mayo Clinic Robert D. and Patricia E. Kern Center for the Science of Health Care Delivery, Mayo Clinic, Rochester, Minnesota

Corresponding Author:

Lang Wu, Center for Clinical and Translational Science, Mayo Clinic, 200 First Street SW, Rochester, MN 55905. Email: Wu.Lang@mayo.edu; Tel: 1-507-293-1756

Running head

diabetic medication and cancer risk and mortality

1). Literature Searching Strategy

Ovid

Database(s): Embase 1988 to 2014 Week 10, Ovid MEDLINE(R) In-Process & Other Non-Indexed Citations and Ovid MEDLINE(R) 1946 to Present, EBM Reviews - Cochrane Central Register of Controlled Trials January 2014, EBM Reviews - Cochrane Database of Systematic Reviews 2005 to January 2014
Search Strategy:

#	Searches	Results
1	exp Diabetes Mellitus/dt [Drug Therapy]	145767
2	exp antidiabetic agent/ae	28533
3	((antidiabet* or "anti-diabet*" or hypoglycemic* or antihyperglycemic* or "anti-hyperglycemic*") adj (agent* or drug* or medication* or compound*)).ti.	4400
4	(aconitan or alagebrium or albiglutide or amlintide or gamolenate or insulin or davalintide or "dehydrotumulosic acid*" or denagliptin or dulaglutide or emeriamine or exendin or ganoderan or hypoglycine or ingliforib or langlenatide or liraglutide or lixisenatide or "managlinat dialanetil" or otelixizumab or piragliatin or pramlintide or proglycosyn or semaglutide or taspoglutide or sulfonylurea or thiazolidinedione* or aleglitazar or alogliptin or anagliptin or arhalofenate or atigliflozin or bisegliptin or buformin or canagliflozin or carmegliptin or cevoglitazar or chiglitazar or dapagliflozin or deriglidole or dutogliptin or empagliflozin or ertiprotafib or ertugliflozin or evogliptin or farglitazar or fenofibrate or gemigliptin or glibenclamide or glitazone or glymidineor gosogliptin or imeglimin or imiglitazar or indeglitazar or ipragliflozin or thiazolidide or limiglidole or linagliptin or linagliptin or linogliride or luseogliflozin or meglitinide or melogliptin or metformin or midaglizole or mitiglinide or muraglitazar or naglivan or nateglinide or naveglitazar or norasterriquinone or omarigliptin or oxeglitazar or "palmoxiric acid*" or peliglitazar or pemaglitazar or phenformin or pirogliride or ragaglitazar or reglitazar or remogliflozin or repaglinide or saroglitazar or saxagliptin or sergliflozin or sipoglitazar or sitagliptin or sodelglitazar or solabegron or sotagliflozin or succinobucol or sulfonylurea or teneligliptin or tesaglitazar or tibeglisene or tifenazoxide or "tofogliflozinor tormentic acid" or trelagliptin or trestatin or "valine pyrrolidide" or vildagliptin or acetohexamide or carboxytolbutamide or carbutamide or chlorpropamide or diabiphage or gliamilide or glibenclamide or glibornuride or glibutimine or glicaramide or gliclazide or glicondamide or gliflumide or glimepiride or glipalamide or glipentide or glipizide or gliquidone or glisamuride or glisolamide or glisoxepide or glucosulfa or glybuthiazol or glybuzole or glycyclamide or glyhexamide or glyoctamide or glyparamide or glypinamide or glyprothiazol or glysobuzole or hydroxyhexamide or hydroxytolbutamide or metahexamide or tolazamide or tolbutamide or pioglitazone or balaglitazone or ciglitazone or darglitazone or edaglitazone or englitazone or glimepiride or rosiglitazone or lobeglitazone or netoglitazone or rivoglitazone or rosiglitazone or troglitazone or antidiabetics or "anti-diabetics" or hypoglycemics or antihyperglycemics or "anti-hyperglycemics").ti.	272148
5	or/1-4	382330

6	exp Neoplasms/	5097665
7	(cancer* or neoplasm* or neoplastic or paraneoplas* or tumor* or tumor* or neoplasia* or "section 16" or leukemia* or carcinoma* or lymphoma* or Astrocytoma* or glioma* or adenoma* or carcinoid* or Sarcoma* or ostesarcoma* or histiocytoma* or craniopharyngioma* or ependymoma* or chordoma* or "Chronic Myeloproliferative Disorder*" or craniopharyngioma* or "Mycosis Fungoide*" or "Sézary Syndrome*" or Esthesioneuroblastoma* or melanoma* or retinoblastoma* or histeocytoma* or "gestational trophoblastic disease*" or histiocytos* or burkitt* or Macroglobulinemia* or Mesothelioma* or neuroblastoma* or Papillomatos* or paraganglioma* or "pheochromocytoma* multiple myeloma*" or blastoma* or Rhabdomyosarcoma* or nonmelanoma* or metasta*).mp.	6088755
8	6 or 7	6405629
9	5 and 8	39688
10	exp Randomized Controlled Trial/	689657
11	exp triple blind procedure/	56
12	exp Double-Blind Method/	331178
13	exp Single-Blind Method/	48066
14	exp Cohort Studies/	1582654
15	exp longitudinal study/	1010625
16	exp retrospective study/	807641
17	exp prospective study/	657941
18	exp case-control studies/	733734
19	"limit follow up studies to medline only. embase maps to follow up".ti.	0
20	exp follow up studies/	1256131
21	((randomized adj2 study) or (randomized adj2 trial) or (randomised adj2 study) or (randomised adj2 trial) or (doubl* adj blind*) or (doubl* adj mask*) or (singl* adj blind*) or (singl* adj mask*) or (tripl* adj blind*) or (tripl* adj mask*) or (trebl* adj blind*) or (trebl* adj mask*) or "cohort study" or "cohort survey" or "cohort analysis" or "longitudinal study" or "longitudinal survey" or "longitudinal analysis" or "retrospective study" or "retrospective survey" or "retrospective analysis" or "prospective study" or "prospective survey" or "prospective analysis" or "follow-up study" or "follow-up survey" or "follow-up analysis" or "case control study" or "case base study" or "case referrent study" or "case referent study" or "case compeer study" or "case comparison study").mp.	2579129
22	from 20 keep 730794-1217120	486327
23	or/10-18	3035875
24	21 or 22 or 23	3663594
25	9 and 24	4752
26	from 9 keep 23177-39031	15855
27	limit 26 to randomized controlled trial [Limit not valid in CDSR; records were retained]	567

28	25 or 27	4752
29	limit 28 to (book or book series or editorial or erratum or letter or note or addresses or autobiography or bibliography or biography or comment or dictionary or directory or interactive tutorial or interview or lectures or legal cases or legislation or news or newspaper article or overall or patient education handout or periodical index or portraits or published erratum or video-audio media or webcasts) [Limit not valid in Embase,Ovid MEDLINE(R),Ovid MEDLINE(R) In-Process,CCTR,CDSR; records were retained]	180
30	28 not 29	4572
31	30 not (exp animals/ not exp humans/)	4523
32	from 9 keep 39679-39688	10
33	31 or 32	4533
34	remove duplicates from 33	3304

<u>Scopus</u>

1 TITLE((antidiabet* W/1 agent*) or (antidiabet* W/1 drug*) or (antidiabet* W/1 medication*) or (antidiabet* W/1 compound*) or ("anti-diabet*" W/1 agent*) or ("anti-diabet*" W/1 drug*) or ("anti-diabet*" W/1 medication*) or ("anti-diabet*" W/1 compound*) or (hypoglycemic* W/1 agent*) or (hypoglycemic* W/1 drug*) or (hypoglycemic* W/1 medication*) or (hypoglycemic* W/1 compound*) or (antihypoglycemic* W/1 agent*) or (antihypoglycemic* W/1 drug*) or (antihypoglycemic* W/1 medication*) or (antihypoglycemic* W/1 compound*) or ("anti-hypoglycemic*" W/1 agent*) or ("anti-hypoglycemic*" W/1 drug*) or ("anti-hypoglycemic*" W/1 medication*) or ("anti-hypoglycemic*" W/1 compound*))

2 TITLE(aconitan Or alagebrium or albiglutide or amlintide or gamolenate or insulin or davalintide or "dehydrotumulosic acid*" or denagliptin or dulaglutide or emeriamine or exendin or ganoderan or hypoglycine or ingliforib or langlenatide or liraglutide or lixisenatide or "managlinat dialanetil" or otelixizumab or piragliatin or pramlintide or proglycosyn or semaglutide or taspoglutide or sulfonylurea or thiazolidinedione* or aleglitazar or alogliptin or anagliptin or arhalofenate or atigliflozin or bisegliptin or buformin or canagliflozin or carmegliptin or cevoglitazar or chiglitazar or dapagliflozin or deriglidole or dutogliptin or empagliflozin or ertiprotafib or ertugliflozin or evogliptin or farglitazar or fenofibrate or gemigliptin or glibenclamide or glitazone or glymidine or gosogliptin or imeglimin or imiglitazar or indeglitazar or ipragliflozin or thiazolidide or limiglidole or linagliptin or linagliptin or linogliride or luseogliflozin or meglitinide or melogliptin or metformin or midaglizole or mitiglinide or muraglitazar or naglivan or nateglinide or naveglitazar or norasterriquinone or omarigliptin or oxeglitazar or "palmoxiric acid*" or peliglitazar or pemaglitazar or phenformin or pirogliride or ragaglitazar or reglitazar or remogliflozin or repaglinide or saroglitazar or saxagliptin or sergliflozin or sipoglitazar or sitagliptin or sodelglitazar or solabegron or sotagliflozin or succinobucol or sulfonylurea or teneligliptin or tesaglitazar or tibeglisene or tifenazoxide or tofogliflozin or "tormentic acid" or trelagliptin or trestatin or "valine pyrrolidide" or vildagliptin or acetohexamide or carboxytolbutamide or carbutamide or chlorpropamide or diabiphage or gliamilide or glibenclamide or glibornuride or glibutimine or glicaramide or gliclazide or glicondamide or gliflumide or glimepiride or glipalamide or glipentide or glipizide or gliquidone or glisamuride or glisolamide or glisoxepide or glucosulfa or glybuthiazol or glybuzole or glycyclamide or glyhexamide or glyoctamide or glyparamide or glypinamide or glyprothiazol or glysobuzole or hydroxyhexamide or hydroxytolbutamide or metahexamide or tolazamide or tolbutamide or pioglitazone or balaglitazone or ciglitazone or darglitazone or edaglitazone or englitazone or glimepiride or rosiglitazone or lobeglitazone or netoglitazone or rivoglitazone or rosiglitazone or troglitazone or

antidiabetics or "anti-diabetics" or hypoglycemics or antihyperglycemics or "anti-hyperglycemics")

3 TITLE-ABS-KEY(cancer* or neoplasm* or neoplastic or paraneoplas* or tumor* or tumor* or neoplasia* or "section 16" or leukemia* or carcinoma* or lymphoma* or Astrocytoma* or glioma* or adenoma* or carcinoid* or Sarcoma* or ostesarcoma* or histiocytoma* or craniopharyngioma* or ependymoma* or chordoma* or "Chronic Myeloproliferative Disorder*" or craniopharyngioma* or "Mycosis Fungoide*" or "Sézary Syndrome*" or Esthesioneuroblastoma* or melanoma* or retinoblastoma* or histeocytoma* or "gestational trophoblastic disease*" or histiocytos* or burkitt* or Macroglobulinemia* or Mesothelioma* or neuroblastoma* or Papillomatos* or paraganglioma* or "pheochromocytoma* multiple myeloma*" or blastoma* or Rhabdomyosarcoma* or nonmelanoma* or metasta*)

4 TITLE-ABS-KEY((randomized W/2 study) OR (randomized W/2 trial) OR (randomised W/2 study) OR (randomised W/2 trial) OR (doubl* W/1 blind*) or (doubl* W/1 mask*) or (singl* W/1 blind*) or (singl* W/1 mask*) or (tripl* W/1 blind*) or (tripl* W/1 mask*) or (trebl* W/1 blind*) or (trebl* W/1 mask*) or "cohort study" OR "cohort survey" OR "cohort analysis" OR "longitudinal study" OR "longitudinal survey" OR "longitudinal analysis" OR "retrospective study" OR "retrospective survey" or "retrospective analysis" OR "prospective study" OR "prospective survey" OR "prospective analysis" OR "follow-up study" OR "follow-up survey" OR "follow-up analysis" OR"case control study" or "case base study" or "case referrent study" or "case referent study" or "case compeer study" or "case comparison study")

5 (1 or 2) and 3 and 4

6 PMID(0*) OR PMID(1*) OR PMID(2*) OR PMID(3*) OR PMID(4*) OR PMID(5*) OR PMID(6*) OR PMID(7*) OR PMID(8*) OR PMID(9*)

7 5 and not 6

8 DOCTYPE(le) OR DOCTYPE(ed) OR DOCTYPE(bk) OR DOCTYPE(er) OR DOCTYPE(no) OR DOCTYPE(sh)

9 7 and not 8

2). Number of included studies for each specific kind of ADMs

Type of ADM	Cancer Incidence				Cancer Mortality			
	Overall	Cohort study	Case-Control study	Clinical trial	Overall	Cohort study	Case-Control study	Clinical trial
Insulin	73	26	34	13	12	10	-	2
Metformin	66	21	22	23	12	6	-	6
sulfonylureas	72	16	18	38	12	4	-	8
TZD	120	12	15	93	16	-	-	16
DPP-4 inhibitors	62	2	1	59	1	-	-	1
alpha-glucosidase inhibitors	13	2	6	5	2	-	-	2
glinides	8	3	3	2	-	-	-	-
GLP-1 agonists	16	2	-	14	-	-	-	-
dapagliflozin	7	-	-	7				

3). Supplementary references for Table 1 and 2

1. Schlesinger S, Aleksandrova K, Pischon T, Jenab M, Fedirko V, Trepo E, et al. Diabetes mellitus, insulin treatment, diabetes duration, and risk of biliary tract cancer and hepatocellular carcinoma in a European Cohort. Ann Oncol. 2013; 24(9): 2449-55.
2. Sluik D, Boeing H, Montonen J, Kaaks R, Lukanova A, Sandbaek A, et al. HbA1c measured in stored erythrocytes is positively linearly associated with mortality in individuals with diabetes mellitus. PLoS ONE. 2012; 7 (6)(e38877).
3. Mathieu C, Barnett AH, Brath H, Conget I, De Castro JJ, Goke R, et al. Effectiveness and tolerability of second-line therapy with vildagliptin vs. other oral agents in type 2 diabetes: A real-life worldwide observational study (EDGE). Int J Clin Pract. 2013; 67 (10): 947-56.
4. Geraldine N, Marc A, Carla T, Chantal M, Stefaan B, Welcome W, et al. Relation between diabetes, metformin treatment and the occurrence of malignancies in a Belgian primary care setting. Diabetes Res Clin Pract. 2012; 97(2): 331-6.
5. Bowker SL, Yasui Y, Veugelers P, Johnson JA. Glucose-lowering agents and cancer mortality rates in type 2 diabetes: assessing effects of time-varying exposure. Diabetologia. 2010; 53(8): 1631-7.
6. Gu Y, Wang C, Zheng Y, Hou X, Mo Y, Yu W, et al. Cancer incidence and mortality in patients with type 2 diabetes treated with human insulin: a cohort study in Shanghai. PLoS ONE. 2013; 8(1): e53411.
7. Carstensen B, Witte DR, Friis S. Cancer occurrence in Danish diabetic patients: duration and insulin effects.[Erratum appears in Diabetologia. 2012 Apr;55(4):1223]. Diabetologia. 2012; 55(4): 948-58.
8. Bazelier MT, de Vries F, Vestergaard P, Leufkens HGM, De Bruin ML. Use of thiazolidinediones and risk of bladder cancer: disease or drugs? Curr Drug Saf. 2013; 8(5): 364-70.
9. Nkontchou G, Cosson E, Aout M, Mahmoudi A, Bourcier V, Charif I, et al. Impact of metformin on the prognosis of cirrhosis induced by viral hepatitis C in diabetic patients. J Clin Endocrinol Metab. 2011; 96(8): 2601-8.
10. Neumann A, Weill A, Ricordeau P, Fagot JP, Alla F, Allemand H. Pioglitazone and risk of bladder cancer among diabetic patients in France: a population-based cohort study. Diabetologia. 2012; 55(7): 1953-62.
11. Hense HW, Kajuter H, Wellmann J, Batzler WU. Cancer incidence in type 2 diabetes patients - First results from a feasibility study of the D2C cohort. Diabetology and Metabolic Syndrome. 2011; 3 (1)(15).
12. Yang X, So W-Y, Ma RCW, Yu LWL, Kong APS, Lee HM, et al. Use of thiazolidinedione and cancer risk in Type 2 diabetes: the Hong Kong diabetes registry. Diabetes Res Clin Pract. 2012; 97(1): e13-7.
13. Yang X, So WY, Ma RCW, Yu LWY, Ko GTC, Kong APS, et al. Use of sulphonylurea and cancer in type 2 diabetes-The Hong Kong Diabetes Registry. Diabetes Res Clin Pract. 2010; 90(3): 343-51.
14. Buchs AE, Silverman BG. Incidence of malignancies in patients with diabetes mellitus and correlation with treatment modalities in a large Israeli health maintenance organization: a historical cohort study. Metabolism. 2011; 60(10): 1379-85.
15. Bo S, Ciccone G, Rosato R, Villois P, Appendino G, Ghigo E, et al. Cancer mortality reduction and metformin: a retrospective cohort study in type 2 diabetic patients. Diabetes Obes Metab. 2012; 14(1): 23-9.
16. Fujimoto K, Hamamoto Y, Honjo S, Kawasaki Y, Mori K, Tatsuoka H, et al. Possible link of pioglitazone with bladder cancer in Japanese patients with type 2 diabetes. Diabetes Res Clin Pract. 2013; 99(2): e21-3.
17. Jin S-M, Song SO, Jung CH, Chang J-S, Suh S, Kang SM, et al. Risk of Bladder Cancer among Patients with Diabetes Treated with a 15 mg Pioglitazone Dose in Korea: A Multi-Center Retrospective Cohort Study. J Korean Med Sci. 2014; 29(2): 238-42.
18. Kim YI, Kim SY, Cho SJ, Park JH, Choi IJ, Lee YJ, et al. Long-term metformin use reduces gastric cancer risk in type 2 diabetics without insulin treatment: A nationwide cohort study. Alimentary Pharmacology and Therapeutics. 2014.
19. Ruiter R, Visser LE, van Herk-Sukel MPP, Coebergh J-WW, Haak HR, Geelhoed-Duijvestijn PH, et al. Lower risk of cancer in patients on metformin in comparison with those on sulfonylurea derivatives: results from a large population-based follow-up study. Diabetes Care. 2012; 35(1): 119-24.
20. Landman GWD, Kleefstra N, van Hateren KJJ, Groenier KH, Gans ROB, Bilo HJG. Metformin associated with lower cancer mortality in type 2 diabetes: ZODIAC-16. Diabetes Care. 2010; 33(2): 322-6.
21. Forssas E, Sund R, Manderbacka K, Arffman M, Ilanne-Parikka P, Keskimaki I. Increased cancer mortality in diabetic people treated with insulin: a register-based follow-up study. BMC Health Serv Res. 2013; 13: 267.

22. Lind M, Fahlen M, Eliasson B, Oden A. The relationship between the exposure time of insulin glargine and risk of breast and prostate cancer: an observational study of the time-dependent effects of antidiabetic treatments in patients with diabetes. Prim Care Diabetes. 2012; 6(1): 53-9.
23. Tseng CH. Diabetes and risk of prostate cancer: a study using the National Health Insurance. Diabetes Care. 2011; 34(3): 616-21.
24. Tseng CH. Type 2 diabetes, smoking, insulin use, and mortality from hepatocellular carcinoma: A 12-year follow-up of a national cohort in Taiwan. Hepatology International. 2013; 7 (2): 693-702.
25. Qiu H, Rhoads GG, Berlin JA, Marcella SW, Demissie K. Initial metformin or sulphonylurea exposure and cancer occurrence among patients with type 2 diabetes mellitus. Diabetes Obes Metab. 2013; 15(4): 349-57.
26. van Staa TP, Patel D, Gallagher AM, de Bruin ML. Glucose-lowering agents and the patterns of risk for cancer: a study with the General Practice Research Database and secondary care data. Diabetologia. 2012; 55(3): 654-65.
27. Currie CJ, Poole CD, Evans M, Peters JR, Morgan CL. Mortality and other important diabetes-related outcomes with insulin vs other antihyperglycemic therapies in type 2 diabetes. J Clin Endocrinol Metab. 2013; 98(2): 668-77.
28. Libby G, Donnelly LA, Donnan PT, Alessi DR, Morris AD, Evans JMM. New users of metformin are at low risk of incident cancer: a cohort study among people with type 2 diabetes. Diabetes Care. 2009; 32(9): 1620-5.
29. Campbell PT, Deka A, Jacobs EJ, Newton CC, Hildebrand JS, McCullough ML, et al. Prospective study reveals associations between colorectal cancer and type 2 diabetes mellitus or insulin use in men. Gastroenterology. 2010; 139(4): 1138-46.
30. Sun GEC, Wells BJ, Yip K, Zimmerman R, Raghavan D, Kattan MW, et al. Gender-specific effects of oral hypoglycaemic agents on cancer risk in type 2 diabetes mellitus. Diabetes, Obesity and Metabolism. 2014; 16 (3): 276-83.
31. Kanadiya MK, Gohel TD, Sanaka MR, Thota PN, Shubrook JH, Jr. Relationship between type-2 diabetes and use of metformin with risk of colorectal adenoma in an American population receiving colonoscopy. Journal of diabetes and its complications. 2013; 27(5): 463-6.
32. Morden NE, Liu SK, Smith J, Mackenzie TA, Skinner J, Korc M. Further exploration of the relationship between insulin glargine and incident cancer: a retrospective cohort study of older Medicare patients. Diabetes Care. 2011; 34(9): 1965-71.
33. Chuang TY, Lewis DA, Spandau DF. Decreased incidence of nonmelanoma skin cancer in patients with type 2 diabetes mellitus using insulin: a pilot study. Br J Dermatol. 2005; 153(3): 552-7.
34. Ferrara A, Lewis JD, Quesenberry CP, Jr., Peng T, Strom BL, Van Den Eeden SK, et al. Cohort study of pioglitazone and cancer incidence in patients with diabetes. Diabetes Care. 2011; 34(4): 923-9.
35. Onitilo AA, Stankowski RV, Berg RL, Engel JM, Glurich I, Williams GM, et al. Type 2 diabetes mellitus, glycemic control, and cancer risk. Eur J Cancer Prev. 2014; 23 (2): 134-40.
36. Romley JA, Goldman DP, Solomon M, McFadden D, Peters AL. Exenatide therapy and the risk of pancreatitis and pancreatic cancer in a privately insured population. Diabetes Technol Ther. 2012; 14(10): 904-11.
37. Funch D, Gydesen H, Tornoe K, Major-Pedersen A, Chan KA. A prospective, claims-based assessment of the risk of pancreatitis and pancreatic cancer with liraglutide compared to other antidiabetic drugs. Diabetes, Obesity and Metabolism. 2014; 16 (3): 273-5.
38. Vallarino C, Perez A, Fusco G, Liang H, Bron M, Manne S, et al. Comparing pioglitazone to insulin with respect to cancer, cardiovascular and bone fracture endpoints, using propensity score weights. Clin Drug Invest. 2013; 33(9): 621-31.
39. Chlebowski RT, McTiernan A, Wactawski-Wende J, Manson JE, Aragaki AK, Rohan T, et al. Diabetes, metformin, and breast cancer in postmenopausal women. J Clin Oncol. 2012; 30(23): 2844-52.
40. Luo J, Chlebowski R, Wactawski-Wende J, Schlecht NF, Tinker L, Margolis KL. Diabetes and lung cancer among postmenopausal women. Diabetes Care. 2012; 35(7): 1485-91.
41. Gates MA, Poole EM, Hu FB, Tworoger SS. Diabetes, insulin use and ovarian cancer incidence. Am J Epidemiol. 2013; Conference: 46th Annual Society for Epidemiologic Research, SER Meeting Boston, MA United States. Conference Start: 20130618 Conference End: 20130621. Conference Publication: (var.pagings). 177: S122.
42. Oliveria SA, Koro CE, Ulcickas Yood M, Sowell M. Cancer incidence among patients treated with antidiabetic pharmacotherapy. Diabetes and Metabolic Syndrome: Clinical Research and Reviews. 2008; 2 (1): 47-57.
43. Velicer CM, Dublin S, White E. Diabetes and the risk of prostate cancer: the role of diabetes treatment and complications. Prostate Cancer Prostatic Dis. 2007; 10(1): 46-51.

44. Mellbin LG, Malmberg K, Norhammar A, Wedel H, Ryden L. Prognostic implications of glucose-lowering treatment in patients with acute myocardial infarction and diabetes: experiences from an extended follow-up of the Diabetes Mellitus Insulin-Glucose Infusion in Acute Myocardial Infarction (DIGAMI) 2 Study. Diabetologia. 2011; 54(6): 1308-17.
45. Bordeleau L, Yakubovich N, Dagenais GR, Rosenstock J, Probstfield J, Chang Yu P, et al. The association of basal insulin glargine and/or n-3 fatty acids with incident cancers in patients with dysglycemia. Diabetes Care. 2014; 37(5): 1360-6.
46. Rodriguez A, Reviriego J, Karamanos V, del Canizo FJ, Vlachogiannis N, Drossinos V. Management of cardiovascular risk factors with pioglitazone combination therapies in type 2 diabetes: an observational cohort study. Cardiovasc Diabetol. 2011; 10: 18.
47. Bosco JLF, Antonsen S, Sorensen HT, Pedersen L, Lash TL. Metformin and incident breast cancer among diabetic women: a population-based case-control study in Denmark. Cancer Epidemiol Biomarkers Prev. 2011; 20(1): 101-11.
48. Chang C-H, Lin J-W, Wu L-C, Lai M-S, Chuang L-M, Chan KA. Association of thiazolidinediones with liver cancer and colorectal cancer in type 2 diabetes mellitus. Hepatology. 2012; 55(5): 1462-72.
49. Dash C, Palmer JR, Boggs DA, Rosenberg L, Adams-Campbell LL. Type 2 diabetes and the risk of colorectal adenomas: Black Women's Health Study. Am J Epidemiol. 2014; 179(1): 112-9.
50. Maisonneuve P, Lowenfels AB, Bueno-de-Mesquita HB, Ghadirian P, Baghurst PA, Zatonski WA, et al. Past medical history and pancreatic cancer risk: Results from a multicenter case-control study. Ann Epidemiol. 2010; 20(2): 92-8.
51. Margel D, Urbach D, Lipscombe LL, Bell CM, Kulkarni G, Austin PC, et al. Association between metformin use and risk of prostate cancer and its grade. J Natl Cancer Inst. 2013; 105(15): 1123-31.
52. Murtola TJ, Tammela TL, Lahtela J, Auvinen A. Antidiabetic medication and prostate cancer risk: a population-based case-control study. Am J Epidemiol. 2008; 168(8): 925-31.
53. Baradaran N, Ahmadi H, Salem S, Lotfi M, Jahani Y, Mehrsai AR, et al. The protective effect of diabetes mellitus against prostate cancer: role of sex hormones. The Prostate. 2009; 69(16): 1744-50.
54. Bonelli L, Aste H, Bovo P, Cavallini G, Felder M, Gusmaroli R, et al. Exocrine pancreatic cancer, cigarette smoking, and diabetes mellitus: A case-control study in Northern Italy. Pancreas. 2003; 27 (2): 143-9.
55. Donadon V, Balbi M, Mas MD, Casarin P, Zanette G. Metformin and reduced risk of hepatocellular carcinoma in diabetic patients with chronic liver disease. Liver Int. 2010; 30(5): 750-8.
56. Monami M, Colombi C, Balzi D, Dicembrini I, Giannini S, Melani C, et al. Metformin and cancer occurrence in insulin-treated type 2 diabetic patients. Diabetes Care. 2011; 34(1): 129-31.
57. Mannucci E, Monami M, Balzi D, Cresci B, Pala L, Melani C, et al. Doses of insulin and its analogues and cancer occurrence in insulin-treated type 2 diabetic patients. Diabetes Care. 2010; 33(9): 1997-2003.
58. Mizuno S, Nakai Y, Isayama H, Yanai A, Takahara N, Miyabayashi K, et al. Risk factors and early signs of pancreatic cancer in diabetes: screening strategy based on diabetes onset age. J Gastroenterol. 2013; 48(2): 238-46.
59. Origasa H, Lee SH, Nakagawa H, Kumagai N, Fuse H, Tobe K. Pioglitazone use and bladder cancer - Hospital-based results from a nested case-control study in japan. Japanese Pharmacology and Therapeutics. 2013; 41 (7): 663-7.
60. Kawaguchi T, Taniguchi E, Morita Y, Shirachi M, Tateishi I, Nagata E, et al. Association of exogenous insulin or sulphonylurea treatment with an increased incidence of hepatoma in patients with hepatitis C virus infection. Liver international : official journal of the International Association for the Study of the Liver. 2010; 30(3): 479-86.
61. Chung YW, Han DS, Park KH, Eun CS, Yoo K-S, Park CK. Insulin therapy and colorectal adenoma risk among patients with Type 2 diabetes mellitus: a case-control study in Korea. Dis Colon Rectum. 2008; 51(5): 593-7.
62. Song SO, Kim KJ, Lee B-W, Kang ES, Cha BS, Lee HC. The risk of bladder cancer in korean diabetic subjects treated with pioglitazone. Diabetes Metab J. 2012; 36(5): 371-8.
63. Dabrowski M. Glycated hemoglobin, diabetes treatment and cancer risk in type 2 diabetes. A case-control study. Annals of Agricultural and Environmental Medicine. 2013; 20 (1): 116-21.
64. Evans JMM, Donnelly LA, Emslie-Smith AM, Alessi DR, Morris AD. Metformin and reduced risk of cancer in diabetic patients. Bmj. 2005; 330(7503): 1304-5.
65. Fortuny J, Benavente Y, Bosch R, Garcia-Villanueva M, de Sevilla AF, de Sanjose S. Type 2 diabetes mellitus, its treatment and risk for lymphoma. Eur J Cancer. 2005; 41(12): 1782-7.
66. Fall K, Garmo H, Gudbjornsdottir S, Stattin P, Zethelius B. Diabetes mellitus and prostate cancer risk; A nationwide case-control study within PCBaSe Sweden. Cancer Epidemiology Biomarkers and Prevention. 2013; 22 (6): 1102-9.

67. Little MW, Pugh TFG, Carey FJ, Ndokera R, Ing H, Clark A, et al. The potential protective effect of metformin against pancreatic cancer: A case-control study in two UK centres. Pancreatology. 2011; Conference: 35th Annual Meeting of the Pancreatic Society of Great Britain and Ireland Sutton Coldfield United Kingdom. Conference Start: 20101202 Conference End: 20101203. Conference Publication: (var.pagings). 11 (3): 311.
68. Grimaldi-Bensouda L, Cameron D, Marty M, Barnett AH, Penault-Llorca F, Pollak M, et al. Risk of breast cancer by individual insulin use: An international multicenter study. Diabetes Care. 2014; 37 (1): 134-43.
69. Vinikoor LC, Long MD, Keku TO, Martin CF, Galanko JA, Sandler RS. The association between diabetes, insulin use, and colorectal cancer among Whites and African Americans. Cancer Epidemiol Biomarkers Prev. 2009; 18(4): 1239-42.
70. Silverman DT, Schiffman M, Everhart J, Goldstein A, Lillemoe KD, Swanson GM, et al. Diabetes mellitus, other medical conditions and familial history of cancer as risk factors for pancreatic cancer. Br J Cancer. 1999; 80 (11): 1830-7.
71. Wright JL, Stanford JL. Metformin use and prostate cancer in Caucasian men: results from a population-based case-control study. Cancer Causes Control. 2009; 20(9): 1617-22.
72. Pierce BL, Plymate S, Ostrander EA, Stanford JL. Diabetes mellitus and prostate cancer risk. The Prostate. 2008; 68(10): 1126-32.
73. Cleveland RJ, North KE, Stevens J, Teitelbaum SL, Neugut AI, Gammon MD. The association of diabetes with breast cancer incidence and mortality in the Long Island Breast Cancer Study Project. Cancer Causes and Control. 2012; 23 (7): 1193-203.
74. Yu MC, Tong MJ, Govindarajan S, Henderson BE. Nonviral risk factors for hepatocellular carcinoma in a low-risk population, the non-Asians of Los Angeles County, California. J Natl Cancer Inst. 1991; 83(24): 1820-6.
75. Sehdev A, Shih YCT, Vekhter B, Lyttle C, Polite BN. The role of metformin for primary prevention in non-elderly diabetic colorectal cancer patients. Journal of Clinical Oncology Conference. 2014; 32(3 SUPPL. 1).
76. Chaiteerakij R, Yang JD, Harmsen WS, Slettedahl SW, Mettler TA, Fredericksen ZS, et al. Risk factors for intrahepatic cholangiocarcinoma: association between metformin use and reduced cancer risk. Hepatology. 2013; 57(2): 648-55.
77. Hachem C, Morgan R, Johnson M, Kuebeler M, El-Serag H. Statins and the risk of colorectal carcinoma: A nested case-control study in veterans with diabetes. Am J Gastroenterol. 2009; 104 (5): 1241-8.
78. Wang F, Gupta S, Holly EA. Diabetes mellitus and pancreatic cancer in a population-based case-control study in the San Francisco Bay Area, California. Cancer epidemiology, biomarkers & prevention : a publication of the American Association for Cancer Research, cosponsored by the American Society of Preventive Oncology. 2006; 15(8): 1458-63.
79. Henry SA, Prizment AE, Anderson KE. Duration of diabetes and pancreatic cancer in a case-control study in the midwest and the Iowa Women's Health Study (IWHS) Cohort. Journal of the Pancreas. 2013; 14 (3): 243-9.
80. Koro C, Barrett S, Qizilbash N. Cancer risks in thiazolidinedione users compared to other anti-diabetic agents. Pharmacoepidemiol Drug Saf. 2007; 16(5): 485-92.
81. Hassan MM, Curley SA, Li D, Kaseb A, Davila M, Abdalla EK, et al. Association of diabetes duration and diabetes treatment with the risk of hepatocellular carcinoma. Cancer. 2010; 116(8): 1938-46.
82. Li D, Yeung S-CJ, Hassan MM, Konopleva M, Abbruzzese JL. Antidiabetic therapies affect risk of pancreatic cancer. Gastroenterology. 2009; 137(2): 482-8.
83. Li D, Tang H, Hassan MM, Holly EA, Bracci PM, Silverman DT. Diabetes and risk of pancreatic cancer: A pooled analysis of three large case-control studies. Cancer Causes and Control. 2011; 22 (2): 189-97.
84. MacKenzie T, Zens MS, Ferrara A, Schned A, Karagas MR. Diabetes and risk of bladder cancer: evidence from a case-control study in New England. Cancer. 2011; 117(7): 1552-6.
85. Eddi R, Karki A, Shah A, DeBari VA, DePasquale JR. Association of type 2 diabetes and colon adenomas. J Gastrointest Cancer. 2012; 43(1): 87-92.
86. Lund SS, Tarnow L, Frandsen M, Nielsen BB, Hansen BV, Pedersen O, et al. Combining insulin with metformin or an insulin secretagogue in non-obese patients with type 2 diabetes: 12 month, randomised, double blind trial. Bmj. 2009; 339: b4324.
87. Goke B, Hershon K, Kerr D, Calle Pascual A, Schweizer A, Foley J, et al. Efficacy and safety of vildagliptin monotherapy during 2-year treatment of drug-naive patients with type 2 diabetes: comparison with metformin. Hormone and metabolic research = Hormon- und Stoffwechselforschung = Hormones et metabolisme. 2008; 40(12): 892-5.
88. Dailey GE, 3rd, Noor MA, Park JS, Bruce S, Fiedorek FT. Glycemic control with glyburide/metformin tablets in combination with rosiglitazone in patients with type 2 diabetes: a randomized, double-blind trial. The American journal of medicine. 2004; 116(4): 223-9.

89. Wong TY, Szeto CC, Chow KM, Leung CB, Lam CW, Li PK. Rosiglitazone reduces insulin requirement and C-reactive protein levels in type 2 diabetic patients receiving peritoneal dialysis. American journal of kidney diseases : the official journal of the National Kidney Foundation. 2005; 46(4): 713-9.
90. Zhou Z, Li X, Huang G, Peng J, Yang L, Yan X, et al. Rosiglitazone combined with insulin preserves islet beta cell function in adult-onset latent autoimmune diabetes (LADA). Diabetes/metabolism research and reviews. 2005; 21(2): 203-8.
91. Derosa G, Gaddi AV, Ciccarelli L, Fogari E, Ghelfi M, Ferrari I, et al. Long-term effect of glimepiride and rosiglitazone on non-conventional cardiovascular risk factors in metformin-treated patients affected by metabolic syndrome: a randomized, double-blind clinical trial. The Journal of international medical research. 2005; 33(3): 284-94.
92. Reynolds LR, Kingsley FJ, Karounos DG, Tannock LR. Differential effects of rosiglitazone and insulin glargine on inflammatory markers, glycemic control, and lipids in type 2 diabetes. Diabetes research and clinical practice. 2007; 77(2): 180-7.
93. Weissman P, Goldstein BJ, Rosenstock J, Waterhouse B, Cobitz AR, Wooddell MJ, et al. Effects of rosiglitazone added to submaximal doses of metformin compared with dose escalation of metformin in type 2 diabetes: the EMPIRE Study. Current medical research and opinion. 2005; 21(12): 2029-35.
94. Ko GT, Tsang PC, Wai HP, Kan EC, Chan HC. Rosiglitazone versus bedtime insulin in the treatment of patients with conventional oral antidiabetic drug failure: a 1-year randomized clinical trial. Adv Ther. 2006; 23(5): 799-808.
95. Garber A, Klein E, Bruce S, Sankoh S, Mohideen P. Metformin-glibenclamide versus metformin plus rosiglitazone in patients with type 2 diabetes inadequately controlled on metformin monotherapy. Diabetes Obes Metab. 2006; 8(2): 156-63.
96. Kelly AS, Thelen AM, Kaiser DR, Gonzalez-Campoy JM, Bank AJ. Rosiglitazone improves endothelial function and inflammation but not asymmetric dimethylarginine or oxidative stress in patients with type 2 diabetes mellitus. Vasc Med. 2007; 12(4): 311-8.
97. Wang G, Wei J, Guan Y, Jin N, Mao J, Wang X. Peroxisome proliferator-activated receptor-gamma agonist rosiglitazone reduces clinical inflammatory responses in type 2 diabetes with coronary artery disease after coronary angioplasty. Metabolism: clinical and experimental. 2005; 54(5): 590-7.
98. Jung HS, Youn BS, Cho YM, Yu KY, Park HJ, Shin CS, et al. The effects of rosiglitazone and metformin on the plasma concentrations of resistin in patients with type 2 diabetes mellitus. Metabolism: clinical and experimental. 2005; 54(3): 314-20.
99. Osman A, Otero J, Brizolara A, Waxman S, Stouffer G, Fitzgerald P, et al. Effect of rosiglitazone on restenosis after coronary stenting in patients with type 2 diabetes. American heart journal. 2004; 147(5): e23.
100. Pfutzner A, Paz-Pacheco E, Allen E, Frederich R, Chen R. Initial combination therapy with saxagliptin and metformin provides sustained glycaemic control and is well tolerated for up to 76 weeks. Diabetes Obes Metab. 2011; 13(6): 567-76.
101. Rosenstock J, Sankoh S, List JF. Glucose-lowering activity of the dipeptidyl peptidase-4 inhibitor saxagliptin in drug-naive patients with type 2 diabetes. Diabetes Obes Metab. 2008; 10(5): 376-86.
102. Hollander P, Li J, Allen E, Chen R. Saxagliptin added to a thiazolidinedione improves glycemic control in patients with type 2 diabetes and inadequate control on thiazolidinedione alone. The Journal of clinical endocrinology and metabolism. 2009; 94(12): 4810-9.
103. Goke B, Gallwitz B, Eriksson J, Hellqvist A, Gause-Nilsson I. Saxagliptin is non-inferior to glipizide in patients with type 2 diabetes mellitus inadequately controlled on metformin alone: a 52-week randomised controlled trial. Int J Clin Pract. 2010; 64(12): 1619-31.
104. Raz I, Hanefeld M, Xu L, Caria C, Williams-Herman D, Khatami H. Efficacy and safety of the dipeptidyl peptidase-4 inhibitor sitagliptin as monotherapy in patients with type 2 diabetes mellitus. Diabetologia. 2006; 49(11): 2564-71.
105. Dejager S, Razac S, Foley JE, Schweizer A. Vildagliptin in drug-naive patients with type 2 diabetes: a 24-week, double-blind, randomized, placebo-controlled, multiple-dose study. Hormone and metabolic research = Hormon- und Stoffwechselforschung = Hormones et metabolisme. 2007; 39(3): 218-23.
106. Chacra AR, Tan GH, Apanovitch A, Ravichandran S, List J, Chen R. Saxagliptin added to a submaximal dose of sulphonylurea improves glycaemic control compared with uptitration of sulphonylurea in patients with type 2 diabetes: a randomised controlled trial. Int J Clin Pract. 2009; 63(9): 1395-406.
107. Rosenstock J, Inzucchi SE, Seufert J, Fleck PR, Wilson CA, Mekki Q. Initial combination therapy with alogliptin and pioglitazone in drug-naive patients with type 2 diabetes. Diabetes Care. 2010; 33(11): 2406-8.

108. Rosenstock J, Baron MA, Dejager S, Mills D, Schweizer A. Comparison of vildagliptin and rosiglitazone monotherapy in patients with type 2 diabetes: a 24-week, double-blind, randomized trial. Diabetes Care. 2007; 30(2): 217-23.
109. Bosi E, Camisasca RP, Collober C, Rochotte E, Garber AJ. Effects of vildagliptin on glucose control over 24 weeks in patients with type 2 diabetes inadequately controlled with metformin. Diabetes Care. 2007; 30(4): 890-5.
110. Garber AJ, Foley JE, Banerji MA, Ebeling P, Gudbjornsdottir S, Camisasca RP, et al. Effects of vildagliptin on glucose control in patients with type 2 diabetes inadequately controlled with a sulphonylurea. Diabetes Obes Metab. 2008; 10(11): 1047-56.
111. Scherbaum WA, Schweizer A, Mari A, Nilsson PM, Lalanne G, Jauffret S, et al. Efficacy and tolerability of vildagliptin in drug-naive patients with type 2 diabetes and mild hyperglycaemia*. Diabetes Obes Metab. 2008; 10(8): 675-82.
112. Ferrannini E, Fonseca V, Zinman B, Matthews D, Ahren B, Byiers S, et al. Fifty-two-week efficacy and safety of vildagliptin vs. glimepiride in patients with type 2 diabetes mellitus inadequately controlled on metformin monotherapy. Diabetes Obes Metab. 2009; 11(2): 157-66.
113. Pan C, Yang W, Barona JP, Wang Y, Niggli M, Mohideen P, et al. Comparison of vildagliptin and acarbose monotherapy in patients with Type 2 diabetes: a 24-week, double-blind, randomized trial. Diabetic medicine : a journal of the British Diabetic Association. 2008; 25(4): 435-41.
114. DeFronzo RA, Hissa MN, Garber AJ, Luiz Gross J, Yuyan Duan R, Ravichandran S, et al. The efficacy and safety of saxagliptin when added to metformin therapy in patients with inadequately controlled type 2 diabetes with metformin alone. Diabetes Care. 2009; 32(9): 1649-55.
115. Nauck MA, Ellis GC, Fleck PR, Wilson CA, Mekki Q. Efficacy and safety of adding the dipeptidyl peptidase-4 inhibitor alogliptin to metformin therapy in patients with type 2 diabetes inadequately controlled with metformin monotherapy: a multicentre, randomised, double-blind, placebo-controlled study. Int J Clin Pract. 2009; 63(1): 46-55.
116. DeFronzo RA, Fleck PR, Wilson CA, Mekki Q. Efficacy and safety of the dipeptidyl peptidase-4 inhibitor alogliptin in patients with type 2 diabetes and inadequate glycemic control: a randomized, double-blind, placebo-controlled study. Diabetes Care. 2008; 31(12): 2315-7.
117. Pratley RE, Kipnes MS, Fleck PR, Wilson C, Mekki Q. Efficacy and safety of the dipeptidyl peptidase-4 inhibitor alogliptin in patients with type 2 diabetes inadequately controlled by glyburide monotherapy. Diabetes Obes Metab. 2009; 11(2): 167-76.
118. Pratley RE, Reusch JE, Fleck PR, Wilson CA, Mekki Q. Efficacy and safety of the dipeptidyl peptidase-4 inhibitor alogliptin added to pioglitazone in patients with type 2 diabetes: a randomized, double-blind, placebo-controlled study. Current medical research and opinion. 2009; 25(10): 2361-71.
119. Bergenstal RM, Wysham C, Macconell L, Malloy J, Walsh B, Yan P, et al. Efficacy and safety of exenatide once weekly versus sitagliptin or pioglitazone as an adjunct to metformin for treatment of type 2 diabetes (DURATION-2): a randomised trial. Lancet. 2010; 376(9739): 431-9.
120. Arechavaleta R, Seck T, Chen Y, Krobot KJ, O'Neill EA, Duran L, et al. Efficacy and safety of treatment with sitagliptin or glimepiride in patients with type 2 diabetes inadequately controlled on metformin monotherapy: a randomized, double-blind, non-inferiority trial. Diabetes Obes Metab. 2011; 13(2): 160-8.
121. Chan JC, Scott R, Arjona Ferreira JC, Sheng D, Gonzalez E, Davies MJ, et al. Safety and efficacy of sitagliptin in patients with type 2 diabetes and chronic renal insufficiency. Diabetes Obes Metab. 2008; 10(7): 545-55.
122. Pratley R, Nauck M, Bailey T, Montanya E, Cuddihy R, Filetti S, et al. One year of liraglutide treatment offers sustained and more effective glycaemic control and weight reduction compared with sitagliptin, both in combination with metformin, in patients with type 2 diabetes: a randomised, parallel-group, open-label trial. Int J Clin Pract. 2011; 65(4): 397-407.
123. Home PD, Lagarenne P. Combined randomised controlled trial experience of malignancies in studies using insulin glargine. Diabetologia. 2009; 52(12): 2499-506.
124. Blickle JF, Hancu N, Piletic M, Profozic V, Shestakova M, Dain MP, et al. Insulin glargine provides greater improvements in glycaemic control vs. intensifying lifestyle management for people with type 2 diabetes treated with OADs and 7-8% A1c levels. The TULIP study. Diabetes Obes Metab. 2009; 11(4): 379-86.
125. Davies MJ, Donnelly R, Barnett AH, Jones S, Nicolay C, Kilcoyne A. Exenatide compared with long-acting insulin to achieve glycaemic control with minimal weight gain in patients with type 2 diabetes: results of the Helping Evaluate Exenatide in patients with diabetes compared with Long-Acting insulin (HEELA) study. Diabetes Obes Metab. 2009; 11(12): 1153-62.

126. Apovian CM, Bergenstal RM, Cuddihy RM, Qu Y, Lenox S, Lewis MS, et al. Effects of exenatide combined with lifestyle modification in patients with type 2 diabetes. The American journal of medicine. 2010; 123(5): 468 e9-17.
127. Liutkus J, Rosas Guzman J, Norwood P, Pop L, Northrup J, Cao D, et al. A placebo-controlled trial of exenatide twice-daily added to thiazolidinediones alone or in combination with metformin. Diabetes Obes Metab. 2010; 12(12): 1058-65.
128. Heine RJ, Van Gaal LF, Johns D, Mihm MJ, Widel MH, Brodows RG. Exenatide versus insulin glargine in patients with suboptimally controlled type 2 diabetes: a randomized trial. Ann Intern Med. 2005; 143(8): 559-69.
129. Nauck MA, Duran S, Kim D, Johns D, Northrup J, Festa A, et al. A comparison of twice-daily exenatide and biphasic insulin aspart in patients with type 2 diabetes who were suboptimally controlled with sulfonylurea and metformin: a non-inferiority study. Diabetologia. 2007; 50(2): 259-67.
130. DeFronzo RA, Triplitt C, Qu Y, Lewis MS, Maggs D, Glass LC. Effects of exenatide plus rosiglitazone on beta-cell function and insulin sensitivity in subjects with type 2 diabetes on metformin. Diabetes Care. 2010; 33(5): 951-7.
131. Gallwitz B, Bohmer M, Segiet T, Molle A, Milek K, Becker B, et al. Exenatide twice daily versus premixed insulin aspart 70/30 in metformin-treated patients with type 2 diabetes: a randomized 26-week study on glycemic control and hypoglycemia. Diabetes Care. 2011; 34(3): 604-6.
132. Gill A, Hoogwerf BJ, Burger J, Bruce S, Macconell L, Yan P, et al. Effect of exenatide on heart rate and blood pressure in subjects with type 2 diabetes mellitus: a double-blind, placebo-controlled, randomized pilot study. Cardiovasc. 2010; 9: 6.
133. Kadowaki T, Namba M, Imaoka T, Yamamura A, Goto W, Boardman MK, et al. Improved glycemic control and reduced bodyweight with exenatide: A double-blind, randomized, phase 3 study in Japanese patients with suboptimally controlled type 2 diabetes over 24 weeks. Journal of diabetes investigation. 2011; 2(3): 210-7.
134. Buse JB, Bergenstal RM, Glass LC, Heilmann CR, Lewis MS, Kwan AY, et al. Use of twice-daily exenatide in Basal insulin-treated patients with type 2 diabetes: a randomized, controlled trial. Ann Intern Med. 2011; 154(2): 103-12.
135. Fonseca V, Schweizer A, Albrecht D, Baron MA, Chang I, Dejager S. Addition of vildagliptin to insulin improves glycaemic control in type 2 diabetes. Diabetologia. 2007; 50(6): 1148-55.
136. Jin HM, Pan Y. Renoprotection provided by losartan in combination with pioglitazone is superior to renoprotection provided by losartan alone in patients with type 2 diabetic nephropathy. Kidney Blood Press Res. 2007; 30(4): 203-11.
137. Derosa G, D'Angelo A, Salvadeo SA, Ferrari I, Fogari E, Gravina A, et al. Modulation of adipokines and vascular remodelling markers during OGTT with acarbose or pioglitazone treatment. Biomed Pharmacother. 2009; 63(10): 723-33.
138. Derosa G, Mereu R, D'Angelo A, Salvadeo SA, Ferrari I, Fogari E, et al. Effect of pioglitazone and acarbose on endothelial inflammation biomarkers during oral glucose tolerance test in diabetic patients treated with sulphonylureas and metformin. Journal of clinical pharmacy and therapeutics. 2010; 35(5): 565-79.
139. Nakamura T, Matsuda T, Kawagoe Y, Ogawa H, Takahashi Y, Sekizuka K, et al. Effect of pioglitazone on carotid intima-media thickness and arterial stiffness in type 2 diabetic nephropathy patients. Metabolism: clinical and experimental. 2004; 53(10): 1382-6.
140. Heliovaara MK, Herz M, Teppo AM, Leinonen E, Ebeling P. Pioglitazone has anti-inflammatory effects in patients with Type 2 diabetes. J Endocrinol Invest. 2007; 30(4): 292-7.
141. Tan MH, Johns D, Strand J, Halse J, Madsbad S, Eriksson JW, et al. Sustained effects of pioglitazone vs. glibenclamide on insulin sensitivity, glycaemic control, and lipid profiles in patients with Type 2 diabetes. Diabetic medicine : a journal of the British Diabetic Association. 2004; 21(8): 859-66.
142. Perriello G, Pampanelli S, Di Pietro C, Brunetti P. Comparison of glycaemic control over 1 year with pioglitazone or gliclazide in patients with Type 2 diabetes. Diabetic medicine : a journal of the British Diabetic Association. 2006; 23(3): 246-52.
143. Matthews DR, Charbonnel BH, Hanefeld M, Brunetti P, Schernthaner G. Long-term therapy with addition of pioglitazone to metformin compared with the addition of gliclazide to metformin in patients with type 2 diabetes: a randomized, comparative study. Diabetes/metabolism research and reviews. 2005; 21(2): 167-74.
144. Yamanouchi T, Sakai T, Igarashi K, Ichiyanagi K, Watanabe H, Kawasaki T. Comparison of metabolic effects of pioglitazone, metformin, and glimepiride over 1 year in Japanese patients with newly diagnosed Type 2 diabetes. Diabet Med. 2005; 22(8): 980-5.

145. Derosa G, Maffioli P, Salvadeo SA, Ferrari I, Gravina A, Mereu R, et al. Direct comparison among oral hypoglycemic agents and their association with insulin resistance evaluated by euglycemic hyperinsulinemic clamp: the 60's study. Metabolism. 2009; 58(8): 1059-66.
146. Tan M, Johns D, Gonzalez Galvez G, Antunez O, Fabian G, Flores-Lozano F, et al. Effects of pioglitazone and glimepiride on glycemic control and insulin sensitivity in Mexican patients with type 2 diabetes mellitus: A multicenter, randomized, double-blind, parallel-group trial. Clin Ther. 2004; 26(5): 680-93.
147. Abe M, Okada K, Maruyama T, Maruyama N, Soma M, Matsumoto K. Clinical effectiveness and safety evaluation of long-term pioglitazone treatment for erythropoietin responsiveness and insulin resistance in type 2 diabetic patients on hemodialysis. Expert Opin Pharmacother. 2010; 11(10): 1611-20.
148. Dargie HJ, Hildebrandt PR, Riegger GA, McMurray JJ, McMorn SO, Roberts JN, et al. A randomized, placebo-controlled trial assessing the effects of rosiglitazone on echocardiographic function and cardiac status in type 2 diabetic patients with New York Heart Association Functional Class I or II Heart Failure. J Am Coll Cardiol. 2007; 49(16): 1696-704.
149. Hedblad B, Zambanini A, Nilsson P, Janzon L, Berglund G. Rosiglitazone and carotid IMT progression rate in a mixed cohort of patients with type 2 diabetes and the insulin resistance syndrome: main results from the Rosiglitazone Atherosclerosis Study. J Intern Med. 2007; 261(3): 293-305.
150. Berberoglu Z, Yazici AC, Demirag NG. Effects of rosiglitazone on bone mineral density and remodelling parameters in Postmenopausal diabetic women: a 2-year follow-up study. Clin Endocrinol (Oxf). 2010; 73(3): 305-12.
151. Rosenstock J, Goldstein BJ, Vinik AI, O'Neill M C, Porter LE, Heise MA, et al. Effect of early addition of rosiglitazone to sulphonylurea therapy in older type 2 diabetes patients (>60 years): the Rosiglitazone Early vs. SULphonylurea Titration (RESULT) study. Diabetes Obes Metab. 2006; 8(1): 49-57.
152. Gerstein HC, Ratner RE, Cannon CP, Serruys PW, Garcia-Garcia HM, van Es GA, et al. Effect of rosiglitazone on progression of coronary atherosclerosis in patients with type 2 diabetes mellitus and coronary artery disease: the assessment on the prevention of progression by rosiglitazone on atherosclerosis in diabetes patients with cardiovascular history trial. Circulation. 2010; 121(10): 1176-87.
153. Gram J, Henriksen JE, Grodum E, Juhl H, Hansen TB, Christiansen C, et al. Pharmacological treatment of the pathogenetic defects in type 2 diabetes: the randomized multicenter South Danish Diabetes Study. Diabetes Care. 2011; 34(1): 27-33.
154. Bertrand OF, Poirier P, Rodes-Cabau J, Rinfret S, Title LM, Dzavik V, et al. Cardiometabolic effects of rosiglitazone in patients with type 2 diabetes and coronary artery bypass grafts: A randomized placebo-controlled clinical trial. Atherosclerosis. 2010; 211(2): 565-73.
155. Mazzone T, Meyer PM, Feinstein SB, Davidson MH, Kondos GT, D'Agostino RB, Sr., et al. Effect of pioglitazone compared with glimepiride on carotid intima-media thickness in type 2 diabetes: a randomized trial. Jama. 2006; 296(21): 2572-81.
156. Nissen SE, Nicholls SJ, Wolski K, Nesto R, Kupfer S, Perez A, et al. Comparison of pioglitazone vs glimepiride on progression of coronary atherosclerosis in patients with type 2 diabetes: the PERISCOPE randomized controlled trial. Jama. 2008; 299(13): 1561-73.
157. Tolman KG, Freston JW, Kupfer S, Perez A. Liver safety in patients with type 2 diabetes treated with pioglitazone: results from a 3-year, randomized, comparator-controlled study in the US. Drug Saf. 2009; 32(9): 787-800.
158. Giles TD, Elkayam U, Bhattacharya M, Perez A, Miller AB. Comparison of pioglitazone vs glyburide in early heart failure: insights from a randomized controlled study of patients with type 2 diabetes and mild cardiac disease. Congest Heart Fail. 2010; 16(3): 111-7.
159. Owens DR, Swallow R, Dugi KA, Woerle HJ. Efficacy and safety of linagliptin in persons with type 2 diabetes inadequately controlled by a combination of metformin and sulphonylurea: a 24-week randomized study. Diabet Med. 2011; 28(11): 1352-61.
160. Gomis R, Espadero RM, Jones R, Woerle HJ, Dugi KA. Efficacy and safety of initial combination therapy with linagliptin and pioglitazone in patients with inadequately controlled type 2 diabetes: a randomized, double-blind, placebo-controlled study. Diabetes Obes Metab. 2011; 13(7): 653-61.
161. Kawamori R, Inagaki N, Araki E, Watada H, Hayashi N, Horie Y, et al. Linagliptin monotherapy provides superior glycaemic control versus placebo or voglibose with comparable safety in Japanese patients with type 2 diabetes: a randomized, placebo and active comparator-controlled, double-blind study. Diabetes Obes Metab. 2012; 14(4): 348-57.

162. Haak T, Meinicke T, Jones R, Weber S, von Eynatten M, Woerle HJ. Initial combination of linagliptin and metformin improves glycaemic control in type 2 diabetes: a randomized, double-blind, placebo-controlled study. Diabetes Obes Metab. 2012; 14(6): 565-74.
163. Lewin AJ, Arvay L, Liu D, Patel S, von Eynatten M, Woerle HJ. Efficacy and tolerability of linagliptin added to a sulfonylurea regimen in patients with inadequately controlled type 2 diabetes mellitus: an 18-week, multicenter, randomized, double-blind, placebo-controlled trial. Clin Ther. 2012; 34(9): 1909-19 e15.
164. Nauck MA, Meininger G, Sheng D, Terranella L, Stein PP. Efficacy and safety of the dipeptidyl peptidase-4 inhibitor, sitagliptin, compared with the sulfonylurea, glipizide, in patients with type 2 diabetes inadequately controlled on metformin alone: a randomized, double-blind, non-inferiority trial. Diabetes Obes Metab. 2007; 9(2): 194-205.
165. Mohan V, Yang W, Son HY, Xu L, Noble L, Langdon RB, et al. Efficacy and safety of sitagliptin in the treatment of patients with type 2 diabetes in China, India, and Korea. Diabetes Res Clin Pract. 2009; 83(1): 106-16.
166. Dobs AS, Goldstein BJ, Aschner P, Horton ES, Umpierrez GE, Duran L, et al. Efficacy and safety of sitagliptin added to ongoing metformin and rosiglitazone combination therapy in a randomized placebo-controlled 54-week trial in patients with type 2 diabetes. J Diabetes. 2013; 5(1): 68-79.
167. Olansky L, Reasner C, Seck TL, Williams-Herman DE, Chen M, Terranella L, et al. A treatment strategy implementing combination therapy with sitagliptin and metformin results in superior glycaemic control versus metformin monotherapy due to a low rate of addition of antihyperglycaemic agents. Diabetes Obes Metab. 2011; 13(9): 841-9.
168. Alba M, Ahren B, Inzucchi SE, Guan Y, Mallick M, Xu L, et al. Sitagliptin and pioglitazone provide complementary effects on postprandial glucose and pancreatic islet cell function. Diabetes Obes Metab. 2013; 15(12): 1101-10.
169. Scott R, Loeys T, Davies MJ, Engel SS. Efficacy and safety of sitagliptin when added to ongoing metformin therapy in patients with type 2 diabetes. Diabetes Obes Metab. 2008; 10(10): 959-69.
170. Rahman S, Ismail AA, Ismail SB, Naing NN, Abdul Rahman AR. Effect of rosiglitazone/ramipril on preclinical vasculopathy in newly diagnosed, untreated diabetes and IGT patients: 1-year randomised, double-blind, placebo-controlled study. Eur J Clin Pharmacol. 2007; 63(8): 733-41.
171. Schweizer A, Couturier A, Foley JE, Dejager S. Comparison between vildagliptin and metformin to sustain reductions in HbA(1c) over 1 year in drug-naive patients with Type 2 diabetes. Diabet Med. 2007; 24(9): 955-61.
172. Kahn SE, Haffner SM, Heise MA, Herman WH, Holman RR, Jones NP, et al. Glycemic durability of rosiglitazone, metformin, or glyburide monotherapy. N Engl J Med. 2006; 355(23): 2427-43.
173. Rosenstock J, Brazg R, Andryuk PJ, Lu K, Stein P. Efficacy and safety of the dipeptidyl peptidase-4 inhibitor sitagliptin added to ongoing pioglitazone therapy in patients with type 2 diabetes: a 24-week, multicenter, randomized, double-blind, placebo-controlled, parallel-group study. Clin Ther. 2006; 28(10): 1556-68.
174. Aschner P, Kipnes MS, Lunceford JK, Sanchez M, Mickel C, Williams-Herman DE. Effect of the dipeptidyl peptidase-4 inhibitor sitagliptin as monotherapy on glycemic control in patients with type 2 diabetes. Diabetes Care. 2006; 29(12): 2632-7.
175. Raz I, Chen Y, Wu M, Hussain S, Kaufman KD, Amatruda JM, et al. Efficacy and safety of sitagliptin added to ongoing metformin therapy in patients with type 2 diabetes. Curr Med Res Opin. 2008; 24(2): 537-50.
176. Vilsboll T, Rosenstock J, Yki-Jarvinen H, Cefalu WT, Chen Y, Luo E, et al. Efficacy and safety of sitagliptin when added to insulin therapy in patients with type 2 diabetes. Diabetes Obes Metab. 2010; 12(2): 167-77.
177. Yoon KH, Shockey GR, Teng R, Golm GT, Thakkar PR, Meehan AG, et al. Effect of initial combination therapy with sitagliptin, a dipeptidyl peptidase-4 inhibitor, and pioglitazone on glycemic control and measures of beta-cell function in patients with type 2 diabetes. Int J Clin Pract. 2011; 65(2): 154-64.
178. Charbonnel B, Karasik A, Liu J, Wu M, Meininger G. Efficacy and safety of the dipeptidyl peptidase-4 inhibitor sitagliptin added to ongoing metformin therapy in patients with type 2 diabetes inadequately controlled with metformin alone. Diabetes Care. 2006; 29(12): 2638-43.
179. Hermansen K, Kipnes M, Luo E, Fanurik D, Khatami H, Stein P. Efficacy and safety of the dipeptidyl peptidase-4 inhibitor, sitagliptin, in patients with type 2 diabetes mellitus inadequately controlled on glimepiride alone or on glimepiride and metformin. Diabetes Obes Metab. 2007; 9(5): 733-45.
180. Bolli G, Dotta F, Colin L, Minic B, Goodman M. Comparison of vildagliptin and pioglitazone in patients with type 2 diabetes inadequately controlled with metformin. Diabetes Obes Metab. 2009; 11(6): 589-95.
181. Del Prato S, Barnett AH, Huisman H, Neubacher D, Woerle HJ, Dugi KA. Effect of linagliptin monotherapy on glycaemic control and markers of beta-cell function in patients with inadequately controlled type 2 diabetes: a randomized controlled trial. Diabetes Obes Metab. 2011; 13(3): 258-67.

182. Taskinen MR, Rosenstock J, Tamminen I, Kubiak R, Patel S, Dugi KA, et al. Safety and efficacy of linagliptin as add-on therapy to metformin in patients with type 2 diabetes: a randomized, double-blind, placebo-controlled study. Diabetes Obes Metab. 2011; 13(1): 65-74.
183. Aschner P, Katzeff HL, Guo H, Sunga S, Williams-Herman D, Kaufman KD, et al. Efficacy and safety of monotherapy of sitagliptin compared with metformin in patients with type 2 diabetes. Diabetes Obes Metab. 2010; 12(3): 252-61.
184. Home PD, Kahn SE, Jones NP, Noronha D, Beck-Nielsen H, Viberti G. Experience of malignancies with oral glucose-lowering drugs in the randomised controlled ADOPT (A Diabetes Outcome Progression Trial) and RECORD (Rosiglitazone Evaluated for Cardiovascular Outcomes and Regulation of Glycaemia in Diabetes) clinical trials. Diabetologia. 2010; 53(9): 1838-45.
185. Hanefeld M, Brunetti P, Schernthaner GH, Matthews DR, Charbonnel BH. One-year glycemic control with a sulfonylurea plus pioglitazone versus a sulfonylurea plus metformin in patients with type 2 diabetes. Diabetes Care. 2004; 27(1): 141-7.
186. Schernthaner G, Matthews DR, Charbonnel B, Hanefeld M, Brunetti P. Efficacy and safety of pioglitazone versus metformin in patients with type 2 diabetes mellitus: a double-blind, randomized trial. J Clin Endocrinol Metab. 2004; 89(12): 6068-76.
187. Cryer DR, Nicholas SP, Henry DH, Mills DJ, Stadel BV. Comparative outcomes study of metformin intervention versus conventional approach the COSMIC Approach Study. Diabetes Care. 2005; 28(3): 539-43.
188. Williams-Herman D, Johnson J, Teng R, Golm G, Kaufman KD, Goldstein BJ, et al. Efficacy and safety of sitagliptin and metformin as initial combination therapy and as monotherapy over 2 years in patients with type 2 diabetes. Diabetes Obes Metab. 2010; 12(5): 442-51.
189. Effect of intensive blood-glucose control with metformin on complications in overweight patients with type 2 diabetes (UKPDS 34). UK Prospective Diabetes Study (UKPDS) Group. Lancet. 1998; 352(9131): 854-65.
190. Ferrannini E, Ramos SJ, Salsali A, Tang W, List JF. Dapagliflozin monotherapy in type 2 diabetic patients with inadequate glycemic control by diet and exercise: a randomized, double-blind, placebo-controlled, phase 3 trial. Diabetes Care. 2010; 33(10): 2217-24.
191. Bailey CJ, Gross JL, Pieters A, Bastien A, List JF. Effect of dapagliflozin in patients with type 2 diabetes who have inadequate glycaemic control with metformin: a randomised, double-blind, placebo-controlled trial. Lancet. 2010; 375(9733): 2223-33.
192. Nauck MA, Del Prato S, Meier JJ, Duran-Garcia S, Rohwedder K, Elze M, et al. Dapagliflozin versus glipizide as add-on therapy in patients with type 2 diabetes who have inadequate glycemic control with metformin: a randomized, 52-week, double-blind, active-controlled noninferiority trial. Diabetes Care. 2011; 34(9): 2015-22.
193. Jain R, Osei K, Kupfer S, Perez AT, Zhang J. Long-term safety of pioglitazone versus glyburide in patients with recently diagnosed type 2 diabetes mellitus. Pharmacotherapy. 2006; 26(10): 1388-95.
194. Derumeaux G, Ernande L, Serusclat A, Servan E, Bruckert E, Rousset H, et al. Echocardiographic evidence for valvular toxicity of benfluorex: a double-blind randomised trial in patients with type 2 diabetes mellitus. PLoS One. 2012; 7(6): e38273.
195. Kikuchi M, Abe N, Kato M, Terao S, Mimori N, Tachibana H. Vildagliptin dose-dependently improves glycemic control in Japanese patients with type 2 diabetes mellitus. Diabetes Res Clin Pract. 2009; 83(2): 233-40.
196. Kawamori R, Iwamoto Y, Kadowaki T, Iwasaki M, Kim SW, Woo JT, et al. Effects of insulin glulisine as mono- or add-on therapy in patients with type 2 diabetes mellitus. Diabetes Obes Metab. 2009; 11(9): 900-9.
197. Kaku K, Araki T, Yoshinaka R. Randomized, double-blind, dose-ranging study of TAK-875, a novel GPR40 agonist, in Japanese patients with inadequately controlled type 2 diabetes. Diabetes Care. 2013; 36(2): 245-50.
198. Kendall DM, Rubin CJ, Mohideen P, Ledeine JM, Belder R, Gross J, et al. Improvement of glycemic control, triglycerides, and HDL cholesterol levels with muraglitazar, a dual (alpha/gamma) peroxisome proliferator-activated receptor activator, in patients with type 2 diabetes inadequately controlled with metformin monotherapy: A double-blind, randomized, pioglitazone-comparative study. Diabetes Care. 2006; 29(5): 1016-23.
199. Seino Y, Rasmussen MF, Zdravkovic M, Kaku K. Dose-dependent improvement in glycemia with once-daily liraglutide without hypoglycemia or weight gain: A double-blind, randomized, controlled trial in Japanese patients with type 2 diabetes. Diabetes Res Clin Pract. 2008; 81(2): 161-8.
200. Kadowaki T, Tajima N, Odawara M, Nishii M, Taniguchi T, Ferreira JC. Addition of sitagliptin to ongoing metformin monotherapy improves glycemic control in Japanese patients with type 2 diabetes over 52 weeks. J Diabetes Investig. 2013; 4(2): 174-81.

201. Gallwitz B, Rosenstock J, Rauch T, Bhattacharya S, Patel S, von Eynatten M, et al. 2-year efficacy and safety of linagliptin compared with glimepiride in patients with type 2 diabetes inadequately controlled on metformin: a randomised, double-blind, non-inferiority trial. Lancet. 2012; 380(9840): 475-83.
202. Diamant M, Van Gaal L, Stranks S, Guerci B, MacConell L, Haber H, et al. Safety and efficacy of once-weekly exenatide compared with insulin glargine titrated to target in patients with type 2 diabetes over 84 weeks. Diabetes Care. 2012; 35(4): 683-9.
203. Wilding JP, Woo V, Rohwedder K, Sugg J, Parikh S. Dapagliflozin in patients with type 2 diabetes receiving high doses of insulin: efficacy and safety over 2 years. Diabetes Obes Metab. 2013.
204. Strojek K, Yoon KH, Hruba V, Elze M, Langkilde AM, Parikh S. Effect of dapagliflozin in patients with type 2 diabetes who have inadequate glycaemic control with glimepiride: a randomized, 24-week, double-blind, placebo-controlled trial. Diabetes Obes Metab. 2011; 13(10): 928-38.
205. Rosenstock J, Vico M, Wei L, Salsali A, List JF. Effects of dapagliflozin, an SGLT2 inhibitor, on HbA(1c), body weight, and hypoglycemia risk in patients with type 2 diabetes inadequately controlled on pioglitazone monotherapy. Diabetes Care. 2012; 35(7): 1473-8.
206. Barnett AH, Patel S, Harper R, Toorawa R, Thiemann S, von Eynatten M, et al. Linagliptin monotherapy in type 2 diabetes patients for whom metformin is inappropriate: an 18-week randomized, double-blind, placebo-controlled phase III trial with a 34-week active-controlled extension. Diabetes Obes Metab. 2012; 14(12): 1145-54.
207. Aschner P, Chan J, Owens DR, Picard S, Wang E, Dain MP, et al. Insulin glargine versus sitagliptin in insulin-naive patients with type 2 diabetes mellitus uncontrolled on metformin (EASIE): a multicentre, randomised open-label trial. Lancet. 2012; 379(9833): 2262-9.
208. Bolinder J, Ljunggren O, Johansson L, Wilding J, Langkilde AM, Sjostrom CD, et al. Dapagliflozin maintains glycaemic control while reducing weight and body fat mass over 2 years in patients with type 2 diabetes mellitus inadequately controlled on metformin. Diabetes Obes Metab. 2013.
209. White WB, Cannon CP, Heller SR, Nissen SE, Bergenstal RM, Bakris GL, et al. Alogliptin after acute coronary syndrome in patients with type 2 diabetes. N Engl J Med. 2013; 369(14): 1327-35.
210. Scirica BM, Bhatt DL, Braunwald E, Steg PG, Davidson J, Hirshberg B, et al. Saxagliptin and cardiovascular outcomes in patients with type 2 diabetes mellitus. N Engl J Med. 2013; 369(14): 1317-26.
211. Wang JS, Huang CN, Hung YJ, Kwok CF, Sun JH, Pei D, et al. Acarbose plus metformin fixed-dose combination outperforms acarbose monotherapy for type 2 diabetes. Diabetes Res Clin Pract. 2013; 102(1): 16-24.
212. Seino Y, Hiroi S, Hirayama M, Kaku K. Efficacy and safety of alogliptin added to sulfonylurea in Japanese patients with type 2 diabetes: A randomized, double-blind, placebo-controlled trial with an open-label, long-term extension study. J Diabetes Investig. 2012; 3(6): 517-25.
213. Lund SS, Tarnow L, Stehouwer CD, Schalkwijk CG, Frandsen M, Smidt UM, et al. Targeting hyperglycaemia with either metformin or repaglinide in non-obese patients with type 2 diabetes: results from a randomized crossover trial. Diabetes Obes Metab. 2007; 9(3): 394-407.
214. Erdmann E, Song E, Spanheimer R, van Troostenburg de Bruyn AR, Perez A. Observational follow-up of the PROactive study: a 6-year update. Diabetes Obes Metab. 2014; 16(1): 63-74.

4) Supplementary Figures

Supplementary Figure 1. Pooled analysis of the association between insulin and cancer incidence/mortality

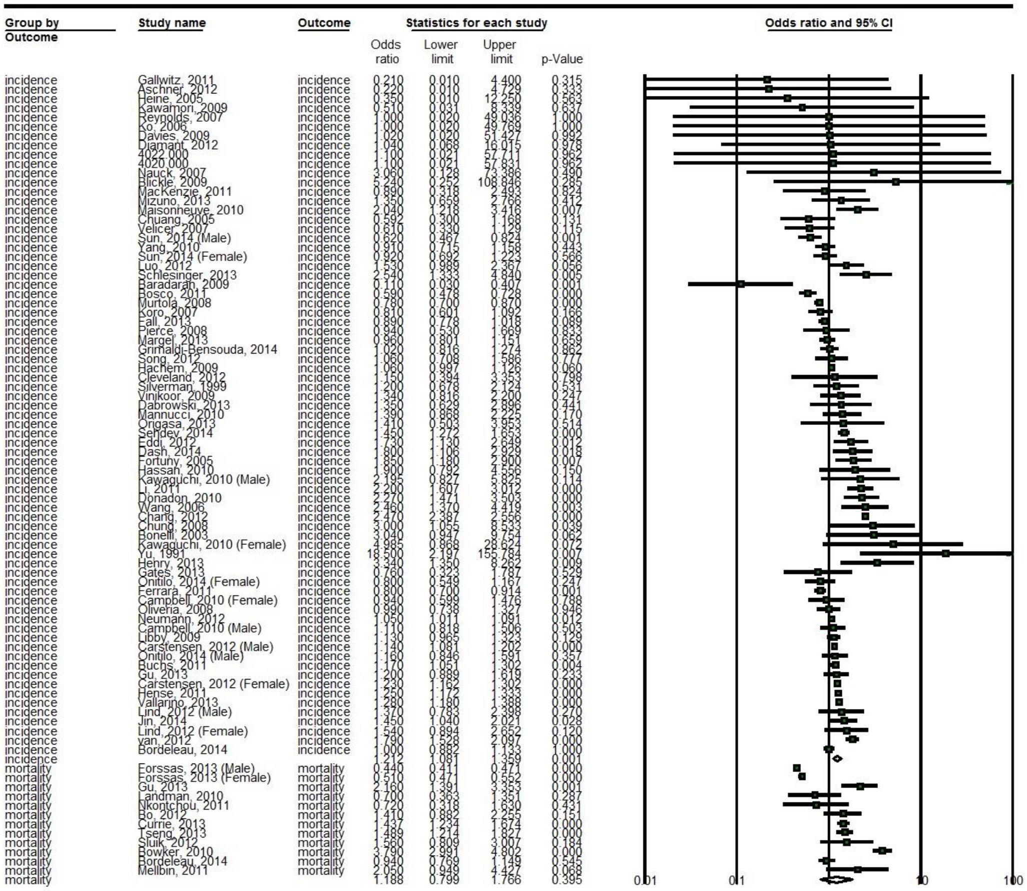

Supplementary Figure 2. Pooled analysis of the association between metformin and cancer incidence/mortality

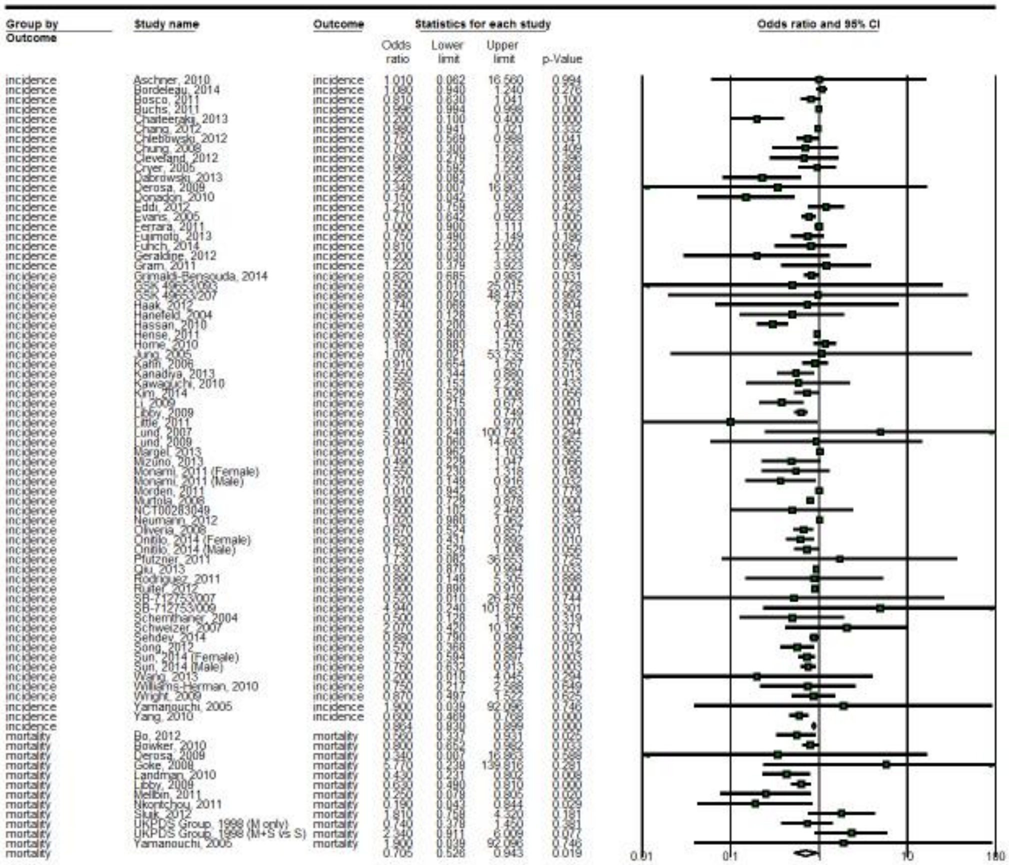

Supplementary Figure 3. Pooled analysis of the association between sulfonylureas and cancer incidence/mortality

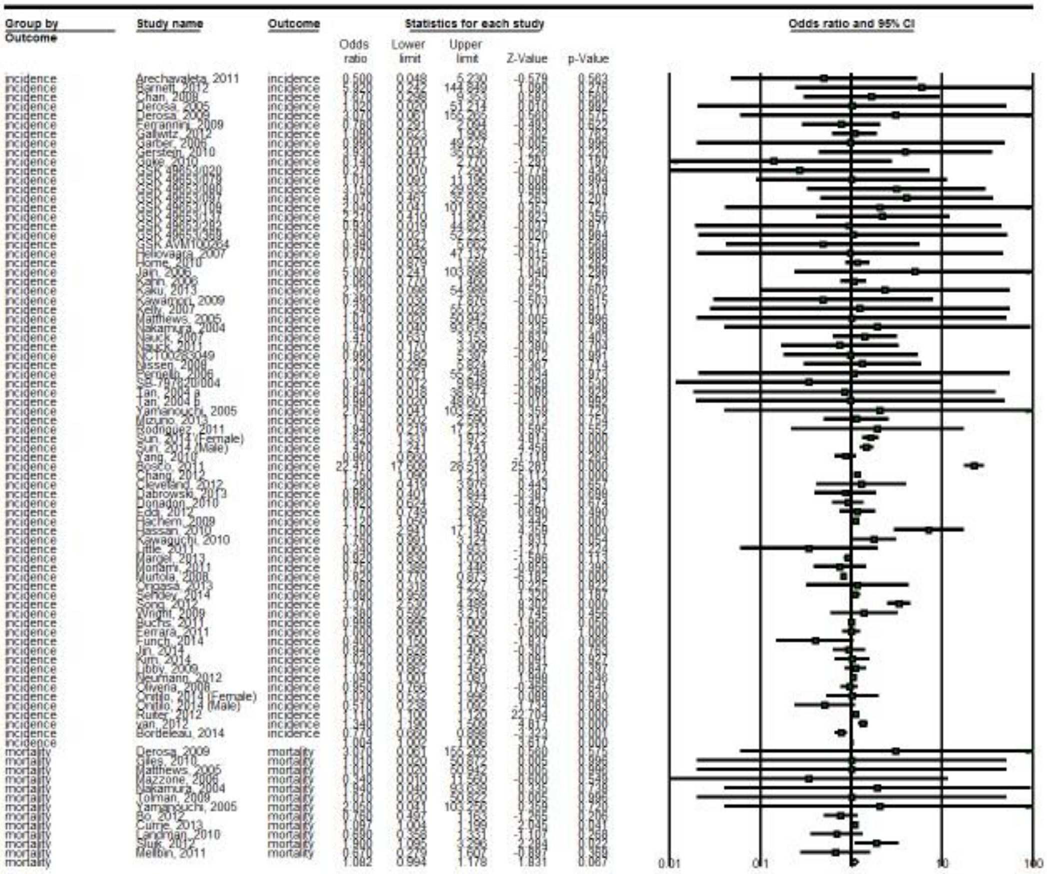

Supplementary Figure 4. Pooled analysis of the association between TZDs and cancer incidence

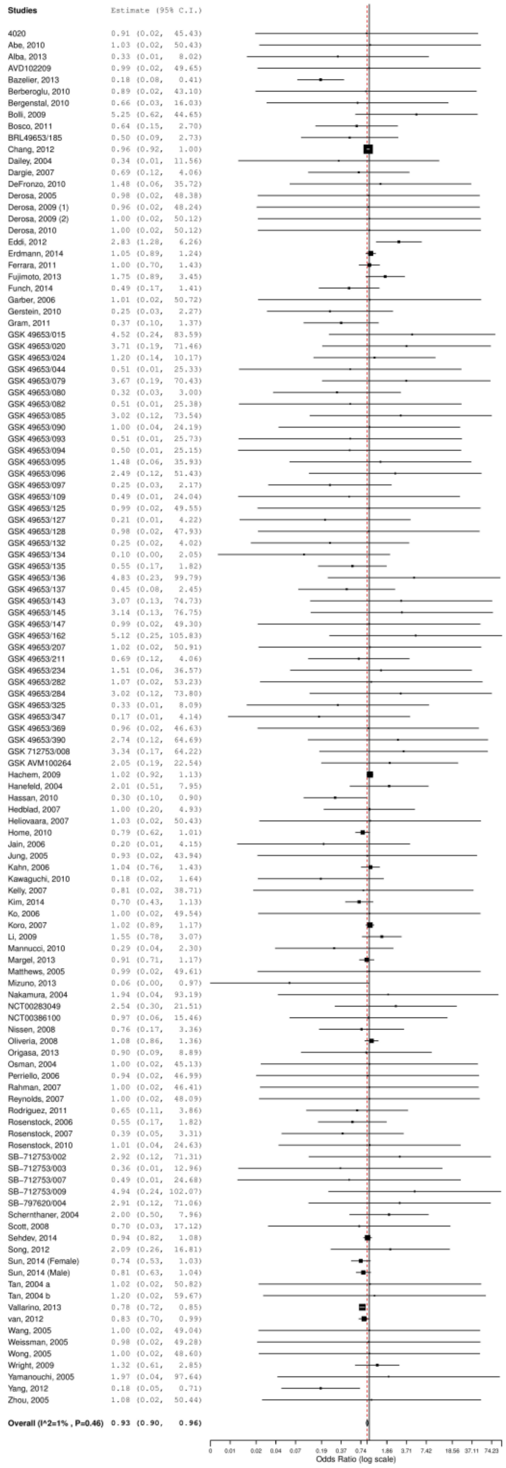

Supplementary Figure 5. Pooled analysis of the association between TZDs and cancer mortality

Study name	Outcome	Odds ratio	Lower limit	Upper limit	p-Value
Abe, 2010	mortality	1.030	0.021	51.721	0.988
Berberoglu, 2010	mortality	0.890	0.019	41.316	0.953
Dargie, 2007	mortality	3.110	0.129	74.943	0.485
Derosa, 2009 (1)	mortality	0.960	0.020	47.148	0.984
Derosa, 2009 (2)	mortality	1.000	0.020	50.060	1.000
Hedblad, 2007	mortality	3.010	0.317	28.540	0.337
Matthews, 2005	mortality	0.990	0.020	49.307	0.996
Nakamura, 2004	mortality	1.940	0.040	93.639	0.738
Rosenstock, 2006	mortality	0.960	0.020	46.947	0.984
Yamanouchi, 2005	mortality	1.970	0.040	97.331	0.733
Bertrand, 2010	mortality	0.970	0.020	47.708	0.988
Giles, 2010	mortality	0.990	0.020	49.212	0.996
Jin, 2007	mortality	1.000	0.020	49.412	1.000
Kendall, 2006	mortality	0.340	0.010	11.560	0.549
Mazzone, 2006	mortality	2.970	0.121	73.067	0.505
Tolman, 2009	mortality	0.990	0.020	49.535	0.996
		1.397	0.574	3.398	0.462

Odds ratio and 95% CI

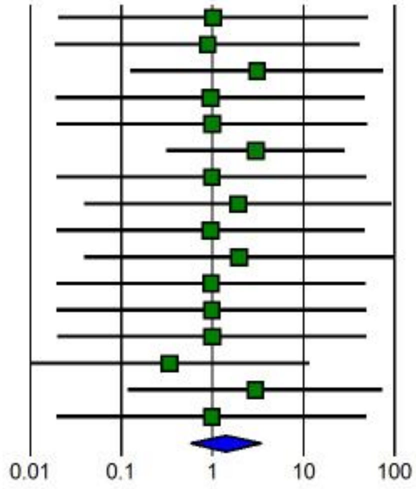

Supplementary Figure 6. Pooled analysis of the association between DPP-4 inhibitor and cancer incidence/mortality

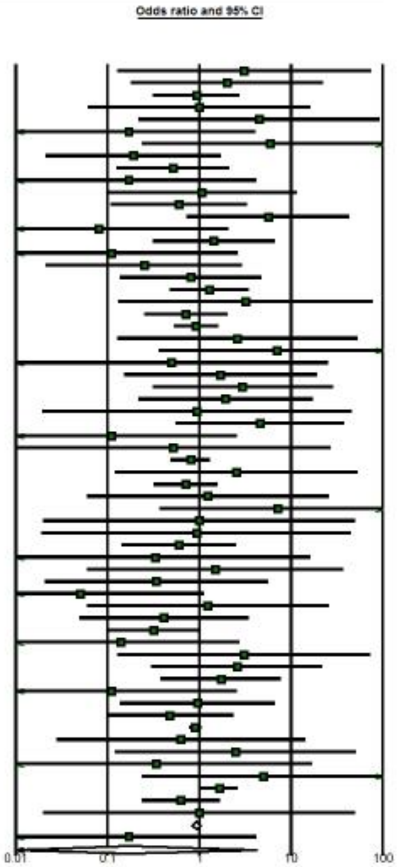

Group by Outcome	Study name	Outcome	Statistics for each study: Odds ratio	Lower limit	Upper limit	p-Value
incidence	Alba, 2013	incidence	3.090	0.129	74.170	0.487
incidence	Arechavaleta, 2011	incidence	2.010	0.181	22.282	0.569
incidence	Aschner, 2006	incidence	0.930	0.317	2.731	0.895
incidence	Aschner, 2010	incidence	0.990	0.061	16.045	0.994
incidence	Aschner, 2012	incidence	4.490	0.218	92.351	0.330
incidence	Barnett, 2012	incidence	0.170	0.007	4.114	0.276
incidence	Bergenstal, 2010	incidence	5.860	0.240	143.036	0.278
incidence	Bolli, 2009	incidence	0.190	0.021	1.710	0.138
incidence	Bosco, 2011	incidence	0.520	0.127	2.129	0.363
incidence	Bosi, 2007	incidence	0.170	0.007	4.119	0.276
incidence	Chacra, 2009	incidence	1.070	0.099	11.574	0.956
incidence	Chan, 2008	incidence	0.600	0.108	3.331	0.559
incidence	Charbonnel, 2006	incidence	5.620	0.730	43.263	0.097
incidence	DeFronzo, 2008	incidence	0.080	0.003	2.055	0.127
incidence	DeFronzo, 2009	incidence	1.430	0.311	6.573	0.646
incidence	Dejager, 2007	incidence	0.110	0.005	2.589	0.171
incidence	Del, 2011	incidence	0.250	0.021	2.915	0.269
incidence	Dobs, 2013	incidence	0.810	0.139	4.728	0.815
incidence	Ferrannini, 2009	incidence	1.280	0.478	3.427	0.623
incidence	Fonseca, 2007	incidence	3.170	0.130	77.195	0.479
incidence	Funch, 2014	incidence	0.710	0.251	2.008	0.519
incidence	Gallwitz, 2012	incidence	0.920	0.529	1.598	0.767
incidence	Garber, 2008	incidence	2.600	0.128	52.956	0.534
incidence	Goke, 2010	incidence	7.030	0.362	136.513	0.198
incidence	Gomis, 2011	incidence	0.500	0.010	25.125	0.729
incidence	Haak, 2012	incidence	1.700	0.153	18.946	0.666
incidence	Hermansen, 2007	incidence	2.960	0.310	28.247	0.346
incidence	Hollander, 2009	incidence	1.930	0.219	17.045	0.554
incidence	Kadowaki, 2013	incidence	0.940	0.019	45.354	0.975
incidence	Kawamori, 2012	incidence	4.550	0.551	37.600	0.160
incidence	Kikuchi, 2009	incidence	0.110	0.005	2.551	0.169
incidence	Lewin, 2012	incidence	0.520	0.010	26.622	0.745
incidence	Mathieu, 2013	incidence	0.800	0.491	1.303	0.370
incidence	Mohan, 2009	incidence	2.540	0.121	53.143	0.548
incidence	Nauck, 2007	incidence	0.710	0.320	1.578	0.400
incidence	Nauck, 2009	incidence	1.240	0.060	25.613	0.889
incidence	NCT00305604	incidence	7.140	0.372	137.110	0.192
incidence	NCT00698932	incidence	1.000	0.020	50.115	1.000
incidence	NCT01028391	incidence	0.930	0.019	44.963	0.971
incidence	Olansky, 2011	incidence	0.600	0.143	2.525	0.486
incidence	Owens, 2011	incidence	0.330	0.007	16.138	0.576
incidence	Pan, 2008	incidence	1.500	0.061	37.083	0.804
incidence	Pfutzner, 2011	incidence	0.340	0.021	5.561	0.449
incidence	Pratley, 2009 a	incidence	0.050	0.002	1.129	0.060
incidence	Pratley, 2009 b	incidence	1.240	0.060	25.663	0.889
incidence	Pratley, 2011	incidence	0.410	0.049	3.416	0.410
incidence	Raz, 2006	incidence	0.320	0.100	1.027	0.055
incidence	Raz, 2008	incidence	0.140	0.007	2.734	0.195
incidence	Rosenstock, 2006	incidence	3.050	0.127	72.965	0.491
incidence	Rosenstock, 2007	incidence	2.570	0.301	21.963	0.389
incidence	Rosenstock, 2008	incidence	1.710	0.380	7.697	0.485
incidence	Rosenstock, 2010	incidence	0.110	0.005	2.561	0.169
incidence	Scherbaum, 2008	incidence	0.960	0.138	6.661	0.967
incidence	Schweizer, 2007	incidence	0.480	0.098	2.342	0.364
incidence	Scirica, 2013	incidence	0.900	0.774	1.046	0.169
incidence	Scott, 2008	incidence	0.630	0.028	14.213	0.771
incidence	Seino, 2012	incidence	2.480	0.120	51.182	0.556
incidence	Taskinen, 2011	incidence	0.340	0.007	16.785	0.588
incidence	Vilsboll, 2010	incidence	4.950	0.239	102.436	0.301
incidence	White, 2013	incidence	1.650	1.051	2.591	0.030
incidence	Williams-Herman, 2010	incidence	0.630	0.238	1.667	0.352
incidence	Yoon, 2011	incidence	0.990	0.020	49.416	0.996
incidence			0.921	0.818	1.036	0.171
mortality	Goke, 2008	mortality	0.170	0.007	4.179	0.278
mortality			0.170	0.007	4.179	0.278

Supplementary Figure 7. Pooled analysis of the association between alpha glucosidase inhibitor and cancer incidence/mortality

Group by Outcome	Study name	Outcome	Odds ratio	Lower limit	Upper limit	p-Value
incidence	Monami, 2011	incidence	0.190	0.010	3.457	0.262
incidence	Origasa, 2013	incidence	0.700	0.205	2.394	0.570
incidence	Bosco, 2011	incidence	1.030	0.633	1.677	0.905
incidence	Chang, 2012	incidence	1.100	1.051	1.151	0.000
incidence	Kawaguchi, 2010	incidence	1.298	0.646	2.609	0.464
incidence	Dabrowski, 2013	incidence	3.590	1.073	12.007	0.038
incidence	Yang, 2010	incidence	0.910	0.638	1.297	0.602
incidence	Tseng, 2011	incidence	1.000	0.493	2.030	1.000
incidence	Kawamori, 2012	incidence	0.410	0.050	3.371	0.407
incidence	Pan, 2008	incidence	0.670	0.029	15.617	0.803
incidence	Derosa, 2009 (2)	incidence	1.000	0.020	50.060	1.000
incidence	Derosa, 2010	incidence	1.000	0.020	50.060	1.000
incidence	Nakamura, 2004	incidence	1.940	0.040	93.639	0.738
incidence			1.097	1.049	1.147	0.000
mortality	Derosa, 2009 (2)	mortality	1.000	0.020	50.060	1.000
mortality	Nakamura, 2004	mortality	1.940	0.040	93.639	0.738
mortality			1.397	0.089	21.945	0.812

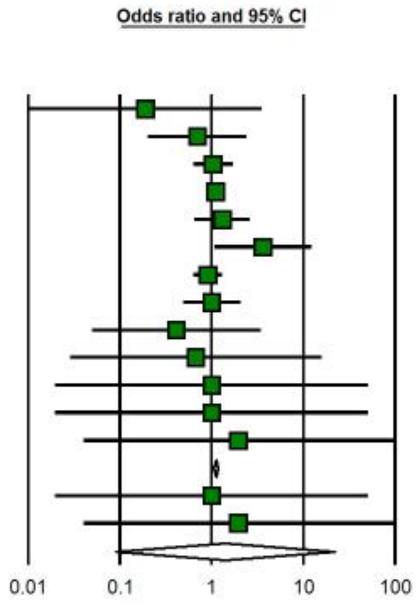

Supplementary Figure 8. Pooled analysis of the association between glinides and cancer incidence

Group by Outcome	Study name	Outcome	Odds ratio	Lower limit	Upper limit	p-Value
incidence	Lund, 2007	incidence	0.200	0.010	4.030	0.294
incidence	Kawaguchi, 2010	incidence	0.515	0.199	1.331	0.171
incidence	Sun, 2014 (Female)	incidence	0.770	0.349	1.697	0.517
incidence	Sun, 2014 (Male)	incidence	0.800	0.433	1.479	0.477
incidence	Wright, 2009	incidence	1.040	0.062	17.449	0.978
incidence	Lund, 2009	incidence	1.060	0.069	16.279	0.967
incidence	Qiu, 2013	incidence	1.170	0.718	1.906	0.528
incidence	Chang, 2012	incidence	1.300	1.241	1.361	0.000
incidence			1.065	0.828	1.370	0.625

Statistics for each study

Odds ratio and 95% CI

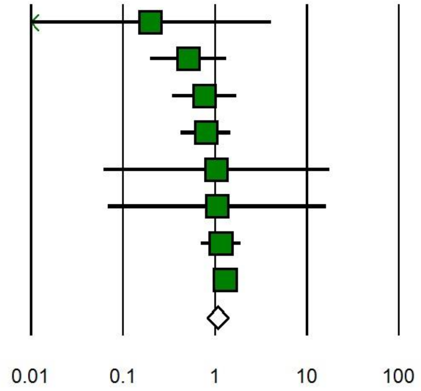

Supplementary Figure 9. Pooled analysis of the association between GLP-1 agonist and cancer incidence

Study name	Outcome	Odds ratio	Lower limit	Upper limit	p-Value
Kadowaki, 2011	incidence	0.240	0.020	2.880	0.260
Nauck, 2007	incidence	0.330	0.012	9.322	0.515
Liutkus, 2010	incidence	0.490	0.010	24.214	0.720
Bergenstal, 2010	incidence	0.690	0.029	16.319	0.818
Seino, 2008	incidence	0.780	0.031	19.536	0.880
Funch, 2014	incidence	0.840	0.200	3.524	0.812
Buse, 2011	incidence	0.890	0.019	42.019	0.953
Gill, 2010	incidence	0.930	0.020	44.261	0.971
Diamant, 2012	incidence	0.960	0.060	15.285	0.977
Davies, 2009	incidence	0.980	0.020	48.577	0.992
Apovian, 2010	incidence	1.020	0.020	51.472	0.992
DeFronzo, 2010	incidence	1.480	0.061	36.111	0.810
Romley, 2012	incidence	1.543	0.489	4.869	0.459
Pratley, 2011	incidence	2.460	0.290	20.879	0.409
Heine, 2005	incidence	2.840	0.118	68.318	0.520
Gallwitz, 2011	incidence	4.720	0.229	97.310	0.315
		1.117	0.606	2.057	0.724

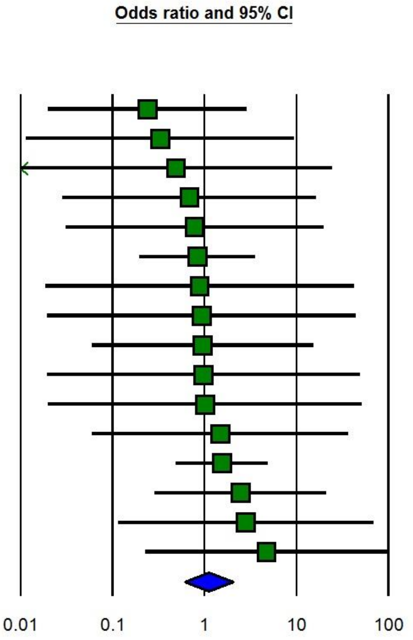

Supplementary Figure 10. Pooled analysis of the association between dapagliflozin and cancer incidence

refid	Outcome	Statistics for each study			
		Odds ratio	Lower limit	Upper limit	p-Value
Bailey, 2010	incidence	0.560	0.138	2.275	0.418
Wilding, 2013	incidence	0.810	0.320	2.050	0.657
Ferrannini, 2010	incidence	0.910	0.109	7.623	0.931
Strojek, 2011	incidence	0.970	0.101	9.349	0.979
Nauck, 2011	incidence	1.340	0.301	5.968	0.701
Rosenstock, 2012	incidence	1.490	0.061	36.664	0.807
Bolinder, 2013	incidence	3.000	0.122	73.836	0.501
		0.899	0.491	1.648	0.731

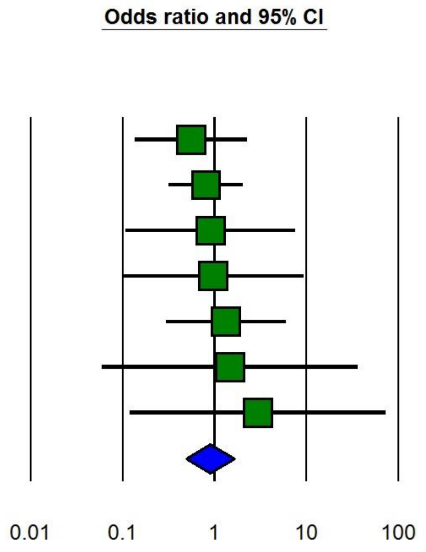

Supplement: Supplementary Information [file srep10147-s1.pdf]
