# Supplementary material for: Pharmacologic Therapy of Diabetes and Overall Cancer Risk and Mortality: A Meta-Analysis of 265 Studies
Source: Sci Rep. 2015 Jun 15;5:10147. doi: 10.1038/srep10147 (PMC4467243; doi:10.1038/srep10147)
Supplement: Supplementary Tables [file srep10147-s2.pdf]

Pharmacologic therapy of diabetes and overall cancer risk and mortality: A Meta-analysis of 265 studies

Lang Wu[1], Jingjing Zhu[2], Larry J. Prokop[3], Mohammad Hassan Murad[4,5]

1 Center for Clinical and Translational Science, Mayo Clinic, Rochester, Minnesota;

2 Program of Quantitative Methods in Education, University of Minnesota, Minneapolis, Minnesota;

3 Mayo Clinic Libraries, Mayo Clinic, Rochester, Minnesota;

4 Division of Preventive Medicine, Mayo Clinic, Rochester, Minnesota;

5 Mayo Clinic Robert D. and Patricia E. Kern Center for the Science of Health Care Delivery, Mayo Clinic, Rochester, Minnesota

Corresponding Author:

Lang Wu, Center for Clinical and Translational Science, Mayo Clinic, 200 First Street SW, Rochester, MN 55905. Email: Wu.Lang@mayo.edu; Tel: 1-507-293-1756

Table S1. Characteristics of included observational studies assessing risk of overall cancer with anti-diabetic medications

First author(reference#), publication year, Country, Study design	Cases/subject (age), duration of follow up	Exposure categories	RR/HR (95% CI)	Matched/Adjusted factors	Outcome	Risk of Bias
**Cohort studies**						
Schlesinger et al ([1]), 2013, European, CS	37/8588 (57.7 ±7.9), mean 8.5 y	Insulin use: no (Ref)	2.54 (1.33, 4.83)	unadjusted	Cancer incidence	moderate
Sluik et al ([2]), 2012, European, CS	120/4516 (mean ~57), median 9.3 y	Insulin use: no medication use (Ref)<br><br>Metformin use exclusively: no medication use (Ref)<br><br>Sulfonylurea use: no medication use (Ref)	1.56 (0.81, 3.01)<br><br>1.81 (0.76, 4.33)<br><br>1.90 (1.10, 3.31)	Age- and center-stratified and adjusted for gender, diabetes duration, self-reported prevalence of heart disease, stroke, or cancer, physical activity, smoking status, educational attainment, body mass index, systolic blood pressure, HbA1c and storage time	Cancer mortality	low
Mathieu et al ([3]), 2013, Europe, Central and Latin America, Asia and Middle East, CS	67/ 43,791 (57.8 ±11.8), 1 y	Vildagliptin use: Comparator (Ref)	0.80 (0.49, 1.30)	unadjusted	Cancer incidence	moderate
Geraldine et al ([4]), 2012, Belgium, CS	NA/4009 (mean ~65), about 5 y	Metformin use: diet group (Ref)	0.20 (0.03, 1.64)	age, sex, weight and initial glycolysated haemoglobin (HbA1c) value	Cancer incidence	low
Bowker et al ([5]), 2010, Canada, CS	407/ 10,309 (mean ~63), mean 5.4 y	Metformin use: no (Ref)<br><br>Insulin use: no (Ref)	0.80 (0.65, 0.98)<br><br>3.79 (2.99, 4.80)	age, sex and chronic disease score	Cancer mortality	low
Gu et al ([6]), 2013, China, CS	268/8774 (mean ~61.5), mean 4 y	Insulin use: no (Ref)<br><br>Insulin use: no (Ref)	1.20 (0.89, 1.62) (CA incidence)<br><br>2.16 (1.39, 3.35) (CA mortality)	age, sex, smoking status, diabetes duration, macrovascular, HbA1c. and concomitant oral glucose lowering agents	Cancer incidence Cancer mortality	low
Carstensen et al ([7]), 2012, Denmark, CS	22826/1.519 million (NA), 15 y	Insulin use: no (Ref)<br>Male:<br><br>Female:	 1.14 (1.08–1.20)<br><br>1.23 (1.16–1.30)	Age, current date of follow-up, date of birth	Cancer incidence	moderate
Bazelier et al ([8]), 2013, Denmark, CS	838/179056 (mean 62.5), mean 5.3 y	TZD use: no (Ref)	0.18 (0.08,0.37)	Sex, age	Cancer incidence	low
Nkontchou et al ([9]),	39/100 (61 ±	Metformin use: no (Ref)	0.19 (0.04, 0.79)	Gender, serum GGT and AFP levels	Cancer	moderate

2011, France, CS	11), median 5 y	Insulin use: only diet use (Ref)	0.72 (0.32,1.64)		mortality	
Neumann et al ([10]), 2012, France, CS	10618/ 1,485,146 (40-79), 3.5 y	Pioglitazone use: no (Ref)<br><br>Rosiglitazone use: no (Ref)<br><br>Metformin use: no (Ref)<br><br>Sulfonylurea use: no (Ref)<br><br>Insulin use: no (Ref)	0.97 (0.90, 1.05)<br><br>0.88 (0.82, 0.95)<br><br>1.02 (0.98, 1.07)<br><br>1.04 (1.00, 1.08)<br><br>1.05 (1.01, 1.11)	age, sex (when applicable), and exposure to glucose-lowering drugs	Cancer incidence	low
Hense et al ([11]), 2011, German, CS	1364/26742 (mean 64), median 3.7 y	Metformin use only: no (Ref)<br><br>Insulin use: no (Ref)	0.95 (0.90, 1.01)<br><br>1.25 (1.17, 1.33)	sex, BMI, diabetes duration, medication at study entry	Cancer incidence	low
Yang et al ([12]), 2012, Hong Kong, CS	270/6074 (mean ~58), median 4.93 y	Thiazolidinedione use: no (Ref)	0.18 (0.04, 0.71)	age, sex, employment status, smoking status, alcohol intake, duration of diabetes, body mass index, glycated hemoglobin, systolic blood pressure at enrolment, low-density lipoprotein cholesterol (LDL-C) related risk indicators (LDL-C _ 3.8 mmol/L and LDL-C < 2.8 mmol/L plus albuminuria), non-linear associations of high-density lipoprotein cholesterol and triglyceride with cancer, use of glibenclamide, gliclazide, metformin, statins, fibrates, renin-angiotensin system (RAS) inhibitors and insulin during follow-up etc	Cancer incidence	low
Yang et al ([13]), 2010, Hong Kong, CS	271/6103 (mean ~57),median 4.91 y	Sulfonylurea use: no (Ref)<br><br>Acarbose use: no (Ref)<br><br>Metformin use: no (Ref)<br><br>Insulin use: no (Ref)	0.86 (0.66, 1.12)<br><br>0.91 (0.64, 1.30)<br><br>0.60 (0.47, 0.77)<br><br>0.91 (0.71, 1.15)	unadjusted	Cancer incidence	low
Buchs et al ([14]), 2011, Israel, CS	2168/ 36 342 (NA), mean 4.5 y	Metformin purchase: no (Ref)<br><br>Sulfonylurea purchase: no (Ref)<br><br>Insulin purchase: no (Ref)	0.996 (0.994, 0.998)<br><br>0.998 (0.996, 1.001)<br><br>1.17 (1.05, 1.30)	age sex, metformin purchases, sulfonylurea purchases, glargine purchases, detemir purchases, other insulin purchases	Cancer incidence	low
Bo et al ([15]), 2012, Italy, CS	122/3685 (mean ~ 69), 4.5 y	Metformin use: diet only (Ref)<br><br>Sulfonylureas use: diet only	0.56 (0.34,0.94)<br><br>0.76 (0.50,1.17)	propensity score	Cancer mortality	low

		(Ref)<br><br>Insulin use: diet only (Ref)	1.41 (0.88,2.25)			
Fujimoto et al ([16]), 2013, Japan, CS	170/ 21,335 (NA), 12 y	pioglitazone use: no (Ref)	1.75 (0.89, 3.45)	Unadjusted	Cancer incidence	low
Jin et al ([17]), 2014, Korea, CS (NC-CS for other medications except pioglitazone)	267/113,193 (mean ~63), 5.5 y	pioglitazone use: no (Ref)<br><br>metformin use: no (Ref)<br><br>Sulfonylurea use: no (Ref)<br><br>Insulin use: no (Ref)<br><br>rosiglitazone use: no (Ref)	1.135 (0.769, 1.677)<br><br>0.75 (0.49, 1.15)<br><br>0.94 (0.63, 1.41)<br><br>1.45 (1.04, 2.02)<br><br>1.42 (0.84, 2.39)	Age, sex	Cancer incidence	low
Kim et al ([18]), 2014, Korea, CS	318/39989 (30-97), median 4.5 y	metformin use: no (Ref)<br><br>Thiazolidinedione use: no (Ref)<br><br>Sulfonylurea use: no (Ref)	0.73 (0.53, 1.01)<br><br>0.70 (0.43, 1.13)<br><br>1.02 (0.67, 1.57)	sex, age, residential area, and other anti-diabetic drug use	Cancer incidence	low
Ruiter et al ([19]), 2012, Netherlands, CS	3552/85,289 (mean ~63), 2.8-4.6 y	metformin use: no (Ref)<br><br>sulfonylurea use: no (Ref)	0.90 (0.89, 0.91)<br><br>1.11 (1.10, 1.12)	Age at first OGLD prescription, sex, number of unique other drugs used in the year before the start of OGLD, number of hospitalizations in the year before the start of OGLD, and calendar time	Cancer incidence	low
Landman et al ([20]), 2010, Netherlands, CS	122/1353 (mean 68), median 9.6 y	metformin use: no (Ref)<br><br>Sulfonylurea use: no (Ref)<br><br>Insulin use: no (Ref)	0.43 (0.23, 0.80)<br><br>0.69 (0.36, 1.34)<br><br>0.70 (0.36, 1.34)	smoking (yes or no), age, sex, diabetes duration, A1C, serum creatinine, BMI, blood pressure, total cholesterol–to–HDL ratio, albuminuria, insulin use, sulfonylurea use, and macrovascular complications	Cancer mortality	low
Forssas et al ([21]), 2013, Finland, CS	Male: 9434/1,095,758<br>Female: 8525/1,137,797 (0-75+), 8 y	Insulin use: no (Ref)<br>Male<br><br>Female	 0.44 (0.41, 0.47)<br><br>0.51 (0.47, 0.55)	Unadjusted	Cancer mortality	moderate
Lind et al ([22]), 2012, Sweden, CS	Male: 185/11613 (mean 52.4), mean 6.9 y<br>Female: 115/7942 (mean 52.2), mean 7 y	Insulin glargine use: no (Ref)<br>Male<br><br>Female	 1.37 (0.78, 2.39)<br><br>1.54 (0.90, 2.67)	time since start of follow-up, age, BMI, type of diabetes, smoking	Cancer incidence	low
Tseng et al ([23]),	362/52131(from	Acarbose use: no (Ref)	1.00 (0.49, 2.02)	age, diabetes duration, comorbidities, other	Cancer	moderate

2011, Taiwan, CS	<40 to >75), 3 y			medications, living region, and occupation	incidence	
Tseng et al ([24]), 2013, Taiwan, CS	1345/88694 (≥25), 12 y	Insulin use: no (Ref)	1.489 (1.214, 1.827)	age, sex, diabetes duration, BMI, smoking	Cancer mortality	low
Qiu et al ([25]), 2013, UK, CS	3507/56844 (mean ~62), mean 2.6-5 y	Metformin use: sulphonylurea use (Ref)<br><br>glinides use: no (Ref)	0.93 (0.87, 1.02)<br><br>1.17 (0.72, 1.91)	Age, sex	Cancer incidence	low
van et al ([26]), 2012, UK, CS	13453/ 206,940 (mean 64), mean ~4 y	Thiazolidinediones use: no (Ref)<br><br>Sulfonylureas use: no (Ref)<br><br>Insulin use: no (Ref)	0.83 (0.70, 0.99)<br><br>1.34 (1.19, 1.51)<br><br>1.79 (1.53, 2.10)	Age, sex, practice, index date, calendar year	Cancer incidence	low
Currie et al ([27]), 2013, UK, CS	6630/84622 (mean ~62), mean ~3 y	Sulfonylureas monotherapy: metformin monotherapy (Ref)<br><br>Insulin monotherapy: metformin monotherapy (Ref)	1.097 (1.004, 1.199)<br><br>1.437 (1.234, 1.674)	age, gender, systolic blood pressure, HbA1c, total cholesterol, serum creatinine, body mass index, smoking status, other risk-factor management, duration of diabetes, prior history of cancer, LVD, microvascular disease, number of contacts with the general practitioner in the year prior to the index date, and the Charlson comorbidity index	Cancer mortality	low
Libby et al ([28]), 2009, UK, CS	771/8170 (35-100), >5.8 y	metformin use: no (Ref)<br>Cancer Incidence<br>Cancer Mortality<br><br>Insulin use: no (Ref)<br><br>Sulfonylureas use: no (Ref)	 0.63 (0.53, 0.75)<br>0.63 (0.49–0.81)<br><br>1.13 (0.97–1.33)<br><br>1.12 (0.87–1.47)	Age, sex, smoking status, Carstairs deprivation category, BMI, A1C	Cancer incidence, Cancer mortality	low
Campbell et al ([29]), 2010, US, CS	Male: 227/ 73,312 (mean 65.1), 15 y<br>Female:108/ 81,663 (mean 63.2), 15 y	Insulin use: no (Ref)<br>Male<br><br>Female	 1.11 (0.82, 1.51)<br><br>0.94 (0.60, 1.48)	age at baseline, education, BMI in 1992, physical activity, nonsteroidal anti-inflammatory drug use, alcohol use, family history of colorectal cancer, and endoscopy history	Cancer incidence	low
Sun et al ([30]), 2014, US, CS	Male: 513/13060 (mean 62)<br>Female: 379/12553 (mean 61), median 1.5 y	metformin use: no (Ref)<br>Male<br>Female<br><br>glinides use: no (Ref)<br>Male<br>Female<br><br>Sulphonylurea use: no (Ref)<br>Male<br>Female	 0.76 (0.63, 0.91)<br>0.73 (0.59, 0.89)<br><br><br>0.80 (0.43, 1.47)<br>0.77 (0.35, 1.70)<br><br><br>1.47 (1.24, 1.74)<br>1.62 (1.33, 1.97)	unadjusted	Cancer incidence	moderate

		Thiazolidinedione use: no (Ref)<br>Male<br>Female<br><br>Insulin use: no (Ref)<br>Male<br>Female	 0.81 (0.62, 1.04)<br>0.74 (0.54, 1.03)<br><br><br>0.62 (0.47, 0.83)<br>0.92 (0.69, 1.22)			
Kanadiya et al ([31]), 2013, US, CS	NA/426 (mean 64), NA	metformin use: no (Ref)	0.55 (0.34, 0.87)	unadjusted	Cancer incidence	moderate
Morden et al ([32]), 2011, US, CS	5466/ 81,681 (mean 77.4), mean 2 y	metformin use: no (Ref)	1.01 (0.94, 1.08)	Age, race/ethnicity, sex, and Part D low-income subsidy status, obesity diagnosis, tobacco exposure, Charlson comorbidities excluding malignancy, diabetes, and tobacco exposure	Cancer incidence	moderate
Chuang et al ([33]), 2005, US, CS	156/6918 (>50), 20 y	Insulin use: no (Ref)	0.592 (0.30, 1.06)	unadjusted	Cancer incidence	moderate
Ferrara et al ([34]), 2011, US, CS	9082/252,467 (≥40), 0.01-8.5 y	TZD use: no (Ref)<br><br>Metformin use: no (Ref)<br><br>Insulin use: no (Ref)<br><br>sulfonylureas use: no (Ref)	1.00 (0.7, 1.3)<br><br>1.00 (0.90, 1.10)<br><br>0.8 (0.7, 0.9)<br><br>1.0 (0.8, 1.1)	age, sex, year of cohort entry, race/ethnicity, income, smoking, glycemic control, diabetes duration, creatinine levels, congestive heart failure, and use of other diabetes medications	Cancer incidence	low
Onitilo et al ([35]), 2014, US, CS	524/9486 (mean 61.7), mean 7.1 y	Insulin use: no (Ref)<br>Male<br>Female<br><br>Metformin use: no (Ref)<br>Male<br>Female<br><br>sulfonylureas use: no (Ref)<br>Male<br>Female	 1.16 (0.84, 1.58)<br>0.80 (0.55, 1.17)<br><br><br>0.73 (0.53, 1.01)<br>0.62 (0.43, 0.89)<br><br><br>0.51 (0.24, 1.10)<br>1.03 (0.53, 1.99)	BMI, age, DM diagnosis date, insurance status, comorbidities, smoking history, and location of residence	Cancer incidence	low
Romley et al ([36]), 2012, US, CS	294/209,306 (mean 64.4), 2 y	Exenatide use: no (Ref)	1.543 (0.489, 4.869)	age, gender, years since diabetes diagnosis, year of analysis, and a set of 19 co-morbid conditions	Cancer incidence	low
Funch et al ([37]), 2014, US, CS	225/NA (median 53), median 1.25 y	Exenatide use: liraglutide use (Ref)<br><br>DPP-4 inhibitors use: liraglutide use (Ref)	0.84 (0.20, 3.52)<br><br><br>0.71 (0.25, 2.00)	age, gender, healthcare utilization and the Diabetes Complications and Severity Index	Cancer incidence	low

		Metformin use: liraglutide use (Ref)<br><br>Sulfonylureas use: liraglutide use (Ref)<br><br>Pioglitazone use: liraglutide use (Ref)	0.81 (0.32, 2.05)<br><br>0.40 (0.15, 1.06)<br><br>0.49 (0.17, 1.41)			
Vallarino et al ([38]), 2013, US, CS	1994/49575 (≥45), mean ~2 y	pioglitazone use: insulin use (Ref)<br><br>Insulin use: pioglitazone use (Ref)	0.78 (0.71, 0.85)<br><br>1.28 (1.18, 1.41)	inverse probability of treatment weights (about 30 variables including age and sex)	Cancer incidence	low
Chlebowski et al ([39]), 2012, US, CS	347/3401 (mean 64), mean 11.8 y	Metformin use: no (Ref)	0.75 (0.57, 0.99)	Age, first-degree relatives with breast cancer, prior breast biopsy, age at menarche, age at menopause, age at parity, age at first live birth, number of months of breastfeeding, education, smoking, alcohol use, body mass index (BMI; linear), physical activity, duration of prior use of estrogen alone and duration of use of estrogen plus progestin, and bilateral oophorectomy	Cancer incidence	low
Luo et al ([40]), 2012, US, CS	108/8154 (mean 64.3), mean 11 y	Insulin use: no (Ref)	1.53 (0.99, 2.37)	Unadjusted	Cancer incidence	low
Gates et al ([41]), 2013, US, CS	55/NA (NA), 34 y	Insulin use: no (Ref)	0.76 (0.32, 1.77)	age, oral contraceptive use, parity, menopausal status, postmenopausal hormone use and body mass index	Cancer incidence	moderate
Oliveria et al ([42]), 2008, US, CS	813/191,223 (mean 56), median 3.9 y	Sulfonylureas monotherapy: no (Ref)<br><br>Metformin monotherapy: no (Ref)<br><br>Insulin monotherapy: no (Ref)<br><br>TZD use: no (Ref)	0.95 (0.76, 1.17)<br><br>0.67 (0.52, 0.85)<br><br>0.99 (0.74, 1.33)<br><br>1.08 (0.86, 1.36)	age, gender, and selected cancer risk factors (history of polyps, ulcerative colitis, Crohn's disease)	Cancer incidence	low
Velicer et al ([43]), 2007, US, CS	67/2878 (50-76), 2 y	Insulin use: no (Ref)	0.61 (0.33, 1.13)	Unadjusted	Cancer incidence	low
Mellbin et al ([44]), 2011, Europe, post-hoc analysis of RCT	37/1145 (mean ~68), median 4.1 y	metformin use: no (Ref)<br><br>Sulfonylureas use: no (Ref)<br><br>Insulin use: no (Ref)	0.25 (0.08, 0.83)<br><br>0.67 (0.28, 1.61)<br><br>2.05 (0.95, 4.43)	sex, age, smoking habits, previous myocardial infarction or previous congestive heart failure recorded at time of hospital admission, creatinine at randomisation, percutaneous transluminal coronary angioplasty or coronary artery bypass grafting during the hospitalisation, and mean updated blood glucose	Cancer mortality	high*
Bordeleau et al ([45]),	953/12, 537	metformin use: no (Ref)	1.08 (0.94, 1.24)	glargine allocation, n-3 fatty acid allocation, baseline	Cancer	low*

2014, international, post-hoc analysis of RCT	(mean 63.5), median 6.2 y	Sulfonylureas use: no (Ref)<br><br>Insulin use: no (Ref)	0.77 (0.66, 0.88) (incidence)<br><br>1.00 (0.88, 1.13) (incidence)<br>0.94 (0.77, 1.15) (mortality)	diabetes status (none, new, established), previous cardiovascular disease status, and current smoking, HbA_{1c}, weight	incidence, cancer mortality	
Rodríguez et al ([46]), 2011, Spain, Greece, CS	5/4585 (mean ~61), 1 y	Sulphonylurea use: no (Ref)<br><br>Pioglitazone use: no (Ref)<br><br>Metformin use: no (Ref)	1.94 (0.22, 17.32)<br><br>0.65 (0.11, 3.86)<br><br>0.89 (0.15, 5.33)	Unadjusted	Cancer incidence	moderate

Table S1. Continued

First author(reference[#]), publication year, Country, Study design	Cases/controls (age)	Exposure categories	OR (95% CI)	Matched/Adjusted factors	Outcome	Risk of Bias
**Case-control studies**						
Bosco et al ([47]), 2011, Denmark, NC-CS	393/3930 (50+)	Metformin use: no (Ref)<br><br>Insulin use: no (Ref)<br><br>Sulfonamides use: no (Ref)<br><br>a-Glucoside inhibitors use: no (Ref)<br><br>Thiazolidinedine use: no (Ref)<br><br>Dipeptidyl peptidase 4 inhibitors use: no (Ref)	0.81 (0.63,0.96)<br><br>0.59 (0.48, 0.73)<br><br>22.41 (17.61, 28.52)<br><br>1.03 (0.63, 1.67)<br><br>0.64 (0.15, 2.70)<br><br>0.52 (0.13, 2.18)	County of residence, complications due to diabetes, clinical obesity, age at index date, postmenopausal hormone use, and multiple imputation to impute missing parity	Cancer incidence	low
Chang et al ([48]), 2012, Taiwan, NC-CS	17941/70559 (mean 67-70)	metformin use: no (Ref)<br><br>Sulfonylurea use: no (Ref)<br><br>Alpha-glucosidase inhibitors use: no (Ref)	0.98 (0.94, 1.02)<br><br>1.15 (1.09, 1.22)<br><br>1.10 (1.05, 1.15)	Age, sex	Cancer incidence	low

		Thiazolidinediones use: no (Ref)<br><br>Glinides use: no (Ref)<br><br>Insulin use: no (Ref)	0.96 (0.92, 1.00)<br><br>1.30 (1.24,1.36)<br><br>2.47 (2.39, 2.56)			
Dash et al ([49]), 2014, US, NC-CS	917/2751 (mean 54.6)	Insulin use: no (Ref)	1.80 (1.11, 2.94)	Age, follow-up time	Cancer incidence	low
Maisonneuve et al ([50]), 2010, Australia, Canada, the Netherlands, and Poland, PC-CS	144/133 (from <40 to 80+)	Insulin use: no (Ref)	2.04 (1.22, 3.42)	Unadjusted	Cancer incidence	moderate
Margel et al ([51]), 2013, Canada, NC-CS	5306/ 26 530 (>66)	metformin use: no (Ref)<br><br>Sulfonylurea use: no (Ref)<br><br>Insulin use: no (Ref)<br><br>TZD use: no (Ref)	1.03 (0.96, 1.10)<br><br>0.92 (0.83, 1.02)<br><br>0.96 (0.8, 1.15)<br><br>0.91 (0.7, 1.17)	age and cohort entry date, same diabetes duration, use of other antidiabetic drugs, weighted adjusted clinical groups comorbidity index, socioeconomic status, rural/urban, and use of cyclooxygenase 2, statins and 5-alpha reductase inhibitors	Cancer incidence	low
Murtola et al ([52]), 2008, Finland, PC-CS	24723/24723 (median 68)	metformin use: no (Ref)<br><br>Sulfonylurea use: no (Ref)<br><br>Insulin use: no (Ref)	0.80 (0.73, 0.88)<br><br>0.82 (0.77, 0.88)<br><br>0.78 (0.70, 0.87)	Age, residential area, and simultaneous use of other medications (aspirin, cholesterol-lowering drugs, or antihypertensive drugs)	Cancer incidence	low
Baradaran et al ([53]), 2009, Iran, HC-CS	21/63 (mean 67-71)	Insulin use: no (Ref)	0.11 (0.03, 0.41)	ethnicity	Cancer incidence	moderate
Bonelli et al ([54]), 2003, Italy, HC-CS	37/30 (18-75)	Insulin use: no (Ref)	3.04 (0.95, 9.78)	gender, age (±5 years)<br>and place of residence	Cancer incidence	moderate
Donadon et al ([55]), 2010, Italy, HC-CS	190/215 (mean ~69)	metformin use: no (Ref)<br><br>Sulfonylurea use: no (Ref)<br><br>Insulin use: no (Ref)	0.15 (0.04, 0.50)<br><br>0.92 (0.63, 1.37)<br><br>2.27 (1.47, 3.50)	sex, age, BMI, HBV and HCV infection, alcohol abuse, ALT level, triglycerides, cholesterol, and DM2 duration	Cancer incidence	low
Monami et al ([56]), 2011, Italy, NC-CS	112/370 (mean 68)	metformin use: no (Ref)<br>male<br>female<br><br>Sulfonylurea use: no (Ref)<br><br>Acarbose use: no (Ref)	 0.37 (0.15, 0.92)<br>0.55 (0.23, 1.32)<br><br>0.75 (0.39, 1.45)<br><br>0.19 (0.01, 3.31)	age, sex, BMI, comorbidity, glargine, and total insulin doses	Cancer incidence	moderate
Mannucci et al ([57]), 2010, Italy, NC-CS	112/370 (mean 68)	TZD use: no (Ref)<br><br>Human insulin use: no (Ref)	0.29 (0.04, 2.30)<br><br>1.39 (0.87, 2.23)	age, sex, BMI, follow-up time	Cancer incidence	moderate

Mizuno et al ([58]), 2013, Japan, HC-CS	40/120 (mean ~66)	Insulin use: no (Ref)<br><br>Sulfonylurea use: no (Ref)<br><br>metformin use: no (Ref)<br><br>pioglitazone use: no (Ref)	1.35 (0.66, 2.77)<br><br>1.14 (0.50, 2.58)<br><br>0.49 (0.23, 1.05)<br><br>0.06 (0.003, 0.97)	Unadjusted	Cancer incidence	moderate
Origasa et al ([59]), 2013, Japan, NC-CS	40/55 (mean 69)	Pioglitazone use: no (Ref)<br><br>Insulin use: no (Ref)<br><br>Sulfonylurea use: no (Ref)<br><br>Alpha-glucosidase inhibitors use: no (Ref)	0.90 (0.09, 8.89)<br><br>1.41 (0.50, 3.93)<br><br>1.16 (0.32, 4.25)<br><br>0.70 (0.20, 2.34)	Sex, age, visit date within 60 days, HbA1c, other antidiabetic medications	Cancer incidence	low
Kawaguchi et al ([60]), 2010, Japan, NC-CS	138/103 (mean ~67)	Insulin use: no (Ref)<br>Male<br>Female<br><br>Sulfonylurea use: no (Ref)<br><br>Alpha-glucosidase inhibitors use: no (Ref)<br><br>Glinide use: no (Ref)<br><br>Metformin use: no (Ref)<br><br>pioglitazone use: no (Ref)	 2.195 (0.827, 5.825)<br>4.985 (0.868, 28.618)<br><br>1.76 (0.99, 3.12)<br><br>1.298 (0.646, 2.609)<br><br>0.515 (0.199, 1.330)<br><br>0.585 (0.153, 2.235)<br><br>0.181 (0.020, 1.641)	Age, sex, BMI, alcohol intake (Z50 g/day), the incidence of cirrhosis, use of other medications, total bilirubin, albumin, AST, ALT, LDH, ALP, g-GTP, platelet count, fasting plasma glucose and HbA1c	Cancer incidence	low
Chung et al ([61]), 2008, Korea, NC-CS	100/100 (mean 66.5)	Insulin use (more than 1 y): no (Ref)<br><br>Metformin use (more than 1 y): no (Ref)	3 (1.1, 8.9)<br><br>0.7 (0.3, 1.4)	Age, sex, body mass index, duration of diabetes, serum levels of hemoglobin A1C and lipids, aspirin therapy	Cancer incidence	moderate
Song et al ([62]), 2012, Korea, HC-CS	329/658 (mean 69.4)	pioglitazone use: no (Ref)<br><br>Sulfonylurea use: no (Ref)<br><br>Insulin use: no (Ref)<br><br>Metformin use: no (Ref)	2.09 (0.26, 16.81)<br><br>3.37 (2.53, 4.49)<br><br>1.06 (0.71, 1.59)<br><br>0.57 (0.37, 0.89)	Age, sex, alcohol, smoking, coexisting cancer, Hemoglobin, Albumin	Cancer incidence	low
Dąbrowski et al ([63]), 2013, Poland, HC-CS	53/53 (44+)	Metformin use: no (Ref)<br><br>Insulin use: no (Ref)	0.228 (0.083, 0.633)<br><br>1.35 (0.63, 2.90)	Age, sex	Cancer incidence	high

		Sulfonylurea use: no (Ref)<br><br>Acarbose use: no (Ref)<br><br>Incretins use: no (Ref)	0.86 (0.40, 1.84)<br><br>3.59 (1.07, 11.97)<br><br>5.19 (0.24, 110.83)			
Evans et al ([64]), 2005, Scotland, PC-CS	923/1846 (mean 73)	Metformin use: no (Ref)	0.77 (0.64, 0.92)	Age, year of diagnosis, sex, index dates, smoking, BMI, blood pressure, postcode rank for material deprivation	Cancer incidence	moderate
Fortuny et al ([65]), 2005, Spain, HC-CS	93/68 (17-96)	Insulin use: no (Ref)	1.85 (1.18, 2.90)	age (±5 years), gender and study centre, socioeconomic status, body mass index in three categories	Cancer incidence	moderate
Fall et al ([66]), 2013, Sweden, PC-CS	1481/9240 (median 71)	Insulin use: no (Ref)	0.89 (0.78, 1.02)	age	Cancer incidence	moderate
Little et al ([67]), 2011, UK, PC-CS	208/NA (median 71)	Metformin use: no (Ref)<br><br>Sulfonylurea use: no (Ref)	0.10 (0.01, 0.94)<br><br>0.34 (0.06, 1.94)	gender, age at diagnosis, cigarette smoking, and type II diabetes	Cancer incidence	moderate
Grimaldi-Bensouda et al ([68]), 2014, UK, Canada, France, PC-CS	775/3050 (mean 66)	Metformin use: no (Ref)<br><br>Insulin use: no (Ref)	0.82 (0.69, 0.99)<br><br>1.02 (0.82, 1.28)	Country, age, recruitment date, diabetes type, management	Cancer incidence	low
Vinikoor et al ([69]), 2009, US, PC-CS	153/142 (mean 64)	Insulin use (≥1 y): no (Ref)	1.34 (0.82, 2.21)	age, sex, and race	Cancer incidence	low
Silverman et al ([70]), 1999, US, PC-CS	64/181 (30-79)	Insulin use: no (Ref)	1.20 (0.68, 2.13)	Age, race, gender	Cancer incidence	low
Wright et al ([71]), 2009, US, PC-CS	97/101 (35-74)	Metformin use: no (Ref)<br><br>Sulfonylurea use: no (Ref)<br><br>TZD use: no (Ref)<br><br>glinides use: no (Ref)	0.87 (0.50, 1.53)<br><br>1.38 (0.59, 3.21)<br><br>1.32 (0.61, 2.85)<br><br>1.04 (0.06, 16.89)	age	Cancer incidence	moderate
Pierce et al ([72]), 2008, US, PC-CS	150/148 (from <50 to 74)	Insulin use: no (Ref)	0.94 (0.53, 1.67)	age	Cancer incidence	low
Cleveland et al ([73]), 2012, US, PC-CS	121/95 (mean 63.6)	Insulin use: no (Ref)<br><br>Metformin use: no (Ref)<br><br>Sulfonylurea use: no (Ref)	1.15 (0.40, 3.40)<br><br>0.68 (0.28, 1.66)<br><br>1.29 (0.42, 3.99)	Age, menopausal status, obesity, and race	Cancer incidence	moderate
Yu et al ([74]), 1991, US, PC-CS	18/14 (18-74)	Insulin use: no (Ref)	18.5 (2.2, 156.0)	age, sex, and race	Cancer incidence	low
Sehdev et al ([75]), 2014, US, PC-CS	2682/5364 (18-64)	Metformin use: no (Ref)<br><br>Insulin use: no (Ref)	0.88 (0.79, 0.98)<br><br>1.45 (1.27, 1.65)	age, sex, and geographical region, IBD, NSAID and statin prescription, coronary artery disease, obesity, polycystic ovary disease	Cancer incidence	low

		Sulfonylurea use: no (Ref)<br><br>TZD use: no (Ref)	1.09 (0.96, 1.24)<br><br>0.94 (0.82, 1.08)			
Chaiteerakij et al ([76]), 2013, US, HC-CS	105/34 (mean ~61)	Metformin use: no (Ref)	0.2 (0.1, 0.4)	Age, sex, ethnicity, and residential area	Cancer incidence	low
Hachem et al ([77]), 2009, US, NC-CS	6080/24320 (mean 74)	Insulin use: no (Ref)<br><br>Sulfonylurea use: no (Ref)<br><br>Thioglitazone use: no (Ref)	1.06 (0.997, 1.14)<br><br>1.12 (1.05, 1.18)<br><br>1.02 (0.92, 1.13)	Age, sex, enrollment date in the diabetic cohort, CRC diagnosis date	Cancer incidence	low
Wang et al ([78]), 2006, US, PC-CS	68/150 (NA)	Insulin use: no (Ref)	2.46 (1.37, 4.42)	Sex, age within 5-year categories	Cancer incidence	moderate
Henry et al ([79]), 2013, US, PC-CS	28/42 (mean 66)	Insulin use: no (Ref)	3.34 (1.35, 8.26)	age, race, sex, education, smoking status, pack-years, and alcohol use	Cancer incidence	low
Koro et al ([80]), 2007, US, NC-CS	1564/7760 (18+)	TZD use: no (Ref)<br><br>Insulin use: no (Ref)	1.02 (0.88, 1.17)<br><br>0.81 (0.60, 1.09)	age, gender, calendar year, and time in the database	Cancer incidence	low
Hassan et al ([81]), 2010, US, HC-CS	140/115 (from ≤40 to ≥70)	Metformin use: no (Ref)<br><br>Sulfonylurea use: no (Ref)<br><br>TZD use: no (Ref)<br><br>Insulin use: no (Ref)	0.3 (0.2, 0.6)<br><br>7.1 (2.9, 16.9)<br><br>0.3 (0.1, 0.7)<br><br>1.9 (0.8, 4.6)	age, sex, race, educational level, cigarette smoking, alcohol drinking, hepatitis C virus, hepatitis B virus, and family history of cancer	Cancer incidence	moderate
Li et al ([82]), 2009, US, HC-CS	259/109 (from ≤50 to ≥70)	Metformin use: no (Ref)<br><br>TZD use: no (Ref)	0.38 (0.22, 0.69)<br><br>1.55 (0.78, 3.07)	age (±5 years), sex, and race, smoking, alcohol, BMI, family history of cancer, diabetes duration, and use of insulin	Cancer incidence	moderate
Li et al ([83]), 2011, US, 1 HC-CS and 2 PC-CS	448/561 (median 63)	Insulin use: no (Ref)	2.2 (1.6, 3.0)	Age, sex, diabetes duration, other antidiabetic medications	Cancer incidence	moderate
MacKenzie et al ([84]), 2011, US, PC-CS	66/25 (mean ~61)	Insulin use: no (Ref)	0.89 (0.32, 2.51)	unadjusted	Cancer incidence	moderate
Eddi et al ([85]), 2012, US, HC-CS	89/136 (mean 71)	Metformin use: no (Ref)<br><br>Sulfonylurea use: no (Ref)<br><br>TZD use: no (Ref)<br><br>Insulin use: no (Ref)	1.21 (0.76, 1.93)<br><br>1.17 (0.75, 1.83)<br><br>2.83 (1.28, 6.26)<br><br>1.73 (1.13, 2.65)	Age, sex	Cancer incidence	moderate

RR: relative risk; CI: confidence interval; NC-CS: nested case-control study; CS: cohort study; PC-CS: population-based case-control study; HC-CS: hospital-based case-control study; NA: not available; NS: not significant; BMI: body mass index.
* quality assessment according to RCT design
# numbered as supplementary references

Table S2. Characteristics of included RCTs assessing risk of overall cancer with anti-diabetic medications

First author(reference #), Country	Trial duration	Treatment groups	Cancer incidences in each group (no. of cancer and no. of group size)	RR (95% CI)	Outcome	Risk of Bias
Lund et al ([86]), Italy	12 months	Metformin+insulin vs repaglinide +insulin	1/52 vs 1/49	glinides use: no (Ref)<br>1.06 (0.07, 16.51)<br>Metformin use: no (Ref)<br>0.94 (0.06, 14.66)	Cancer incidence	high
Göke et al ([87]), US and Europe	52 weeks	Metformin vs vildagliptin	1/158 vs 0/305	DPP-4 inhibitor use: no (Ref)<br>0.17 (0.007, 4.23)<br>Metformin use: no (Ref)<br>5.77 (0.24, 140.92)	Cancer mortality	high
Dailey et al ([88]), US	24 weeks	glyburide/metformin+ rosiglitazone vs glyburide/metformin	0/181 vs 1/184	TZD use: no (Ref)<br>0.34 (0.01, 8.26)	Cancer incidence	high
Wong et al ([89]), Kong Kong	24 weeks	Rosiglitazone+insulin vs insulin	0/26 vs 0/26	TZD use: no (Ref)<br>1.00 (0.02, 48.60)	Cancer incidence	high
Zhou et al ([90]), China	6 months	Rosiglitazone+insulin vs insulin	0/11 vs 0/12	TZD use: no (Ref)<br>1.08 (0.02, 50.44)	Cancer incidence	high
Derosa et al ([91]), Italy	12 months	Rosiglitazone+metformin vs glimepiride+metformin	0/48 vs 0/47	TZD use: no (Ref)<br>0.98 (0.02, 48.38)<br>Sulfonylurea use: no (Ref)<br>1.02 (0.02, 50.42)	Cancer incidence	low
Reynolds et al ([92]), US	24 weeks	Rosiglitazone vs insulin	0/20 vs 0/20	TZD use: no (Ref)<br>1.00 (0.02, 48.09)<br>Insulin use: no (Ref)<br>1.00 (0.02, 48.09)	Cancer incidence	high
Weissman et al ([93]), US	24 weeks	Rosiglitazone+metformin vs metformin	0/358 vs 0/351	TZD use: no (Ref)<br>0.98 (0.02, 49.28)	Cancer incidence	high
Ko et al ([94]), Hong Kong	1 year	Rosiglitazone vs insulin	0/56 vs 0/56	TZD use: no (Ref)<br>1.00 (0.02, 49.54)<br>Insulin use: no (Ref)<br>1.00 (0.02, 49.54)	Cancer incidence	high
Garber et al ([95]), US	24 weeks	Rosiglitazone+metformin vs glibenclamide +metformin	0/158 vs 0/160	TZD use: no (Ref)<br>1.01 (0.02, 50.72)<br>Sulfonylurea use: no (Ref)<br>0.99 (0.02, 49.47)	Cancer incidence	high
Kelly et al ([96]), US	6 months	Rosiglitazone+metformin vs glyburide +metformin	0/20 vs 0/16	TZD use: no (Ref)<br>0.81 (0.02, 38.71)<br>Sulfonylurea use: no (Ref)<br>1.24 (0.03, 59.07)	Cancer incidence	high
Wang et al ([97]),	6 months	Rosiglitazone vs control	0/35 vs 0/35	TZD use: no (Ref)	Cancer	high

China				1.00 (0.02, 49.04)	incidence	
Jung et al ([98]), Korea	6 months	Rosiglitazone vs metformin	0/14 vs 0/13	TZD use: no (Ref)<br>0.93 (0.02, 43.94)<br>Metformin use: no (Ref)<br>1.07 (0.02, 50.44)	Cancer incidence	high
Osman et al ([99]), US	6 months	Rosiglitazone vs placebo	0/8 vs 0/8	TZD use: no (Ref)<br>1.00 (0.02, 45.13)	Cancer incidence	high
GSK 49653/080, US	3 years	Rosiglitazone vs Glyburide	1/104 vs 3/99	TZD use: no (Ref)<br>0.32 (0.03, 3.00)<br>Sulfonylurea use: no (Ref)<br>3.15 (0.33, 29.79)	Cancer incidence	high
GSK 49653/097, US	3 years	Rosiglitazone vs Glyburide	1/122 vs 4/120	TZD use: no (Ref)<br>0.25 (0.03, 2.17)<br>Sulfonylurea use: no (Ref)<br>4.07 (0.46, 35.86)	Cancer incidence	high
GSK 49653/135, US and Canada	2 years	Rosiglitazone+ Glipizide vs Glipizide	4/116 vs 7/111	TZD use: no (Ref)<br>0.55 (0.16, 1.82)	Cancer incidence	high
GSK 49653/211, Europe	52 weeks	Rosiglitazone vs placebo	2/110 vs 3/114	TZD use: no (Ref)<br>0.69 (0.12, 4.06)	Cancer incidence	high
GSK 49653/020, Europe	52 weeks	Rosiglitazone vs glibenclamide	3/384 vs 0/203	TZD use: no (Ref)<br>3.71 (0.19, 71.46)<br>Sulfonylurea use: no (Ref)<br>0.27 (0.01, 5.19)	Cancer incidence	high
GSK AVM100264, German	52 weeks	Rosiglitazone+metformin vs sulphonylurea +metformin	2/294 vs 1/302	TZD use: no (Ref)<br>2.05 (0.19, 22.54)<br>Sulfonylurea use: no (Ref)<br>0.49 (0.04, 5.34)	Cancer incidence	high
GSK 712753/008, Canada	48 weeks	Rosiglitazone+metformin vs metformin	3/284 vs 0/135	TZD use: no (Ref)<br>3.34 (0.17, 64.22)	Cancer incidence	high
GSK 49653/137, US	32 weeks	Rosiglitazone+metformin vs Glyburide+metformin	2/204 vs 4/185	TZD use: no (Ref)<br>0.45 (0.08, 2.45)<br>Sulfonylurea use: no (Ref)<br>2.21 (0.41, 11.90)	Cancer incidence	high
BRL49653/185, Canada	32 weeks	Rosiglitazone vs control	4/563 vs 2/142	TZD use: no (Ref)<br>0.50 (0.09, 2.73)	Cancer incidence	high
SB-712753/003	32 weeks	Rosiglitazone+metformin vs metformin	0/254 vs 1/272	TZD use: no (Ref)<br>0.36 (0.01, 8.72)	Cancer incidence	high
SB-712753/007	32 weeks	Rosiglitazone+metformin vs metformin vs Rosiglitazone	0/155 vs 0/154 vs 0/159	TZD use: no (Ref)<br>0.49 (0.01, 24.68)<br>Metformin use: no (Ref)<br>0.52 (0.01, 25.89)	Cancer incidence	high
GSK 49653/128, Taiwan	24 weeks	Rosiglitazone vs placebo	0/39 vs 0/38	TZD use: no (Ref)<br>0.98 (0.02, 47.93)	Cancer incidence	high
GSK 49653/134,	26 weeks	Rosiglitazone vs placebo	0/561 vs 2/276	TZD use: no (Ref)	Cancer	high

US, Canada and Mexico				0.10 (0.005, 2.05)	incidence	
SB-797620/004, 19 countries internationally	28 weeks	Rosiglitazone vs glimepiride	1/232 vs 0/225	TZD use: no (Ref)<br>2.91 (0.12, 71.06)<br>Sulfonylurea use: no (Ref)<br>0.34 (0.01, 8.39)	Cancer incidence	high
GSK 49653/024, US	26 weeks	Rosiglitazone vs placebo	5/774 vs 1/185	TZD use: no (Ref)<br>1.20 (0.14, 10.17)	Cancer incidence	high
GSK 49653/044, Canada and Mexico	26 weeks	Rosiglitazone vs placebo	0/101 vs 0/51	TZD use: no (Ref)<br>0.51 (0.01, 25.33)	Cancer incidence	high
GSK 49653/079, US	26 weeks	Rosiglitazone vs glibencamide vs Rosiglitazone+ glibencamide	1/104 vs 0/106 vs 2/99	TZD use: no (Ref)<br>3.67 (0.19, 70.43)<br>Sulfonylurea use: no (Ref)<br>1.01 (0.09, 11.06)	Cancer incidence	high
GSK 49653/082, US	26 weeks	Rosiglitazone vs placebo	0/212 vs 0/107	TZD use: no (Ref)<br>0.51 (0.01, 25.38)	Cancer incidence	high
GSK 49653/085, Europe	26 weeks	Rosiglitazone vs placebo	1/138 vs 0/139	TZD use: no (Ref)<br>3.02 (0.12, 73.54)	Cancer incidence	high
GSK 49653/093, US	26 weeks	Rosiglitazone vs metformin vs Rosiglitazone+ metformin	0/107 vs 0/109 vs 0/106	TZD use: no (Ref)<br>0.51 (0.01, 25.73)<br>Metformin use: no (Ref)<br>0.50 (0.01, 25.03)	Cancer incidence	high
GSK 49653/094, US	26 weeks	Rosiglitazone vs placebo	0/232 vs 0/116	TZD use: no (Ref)<br>0.50 (0.01, 25.15)	Cancer incidence	high
GSK 49653/095, US	26 weeks	Rosiglitazone vs placebo	1/196 vs 0/96	TZD use: no (Ref)<br>1.48 (0.06, 35.93)	Cancer incidence	high
GSK 49653/096, US	26 weeks	Rosiglitazone vs placebo	2/232 vs 0/115	TZD use: no (Ref)<br>2.49 (0.12, 51.43)	Cancer incidence	high
GSK 49653/109, US	26 weeks	Rosiglitazone vs glipizide	0/52 vs 0/25	TZD use: no (Ref)<br>0.49 (0.01, 24.04)<br>Sulfonylurea use: no (Ref)<br>2.04 (0.04, 99.88)	Cancer incidence	high
GSK 49653/125, Asia, South America and Africa	26 weeks	Rosiglitazone+ sulphonylurea vs sulphonylurea	0/175 vs 0/173	TZD use: no (Ref)<br>0.99 (0.02, 49.55)	Cancer incidence	high
GSK 49653/127, US and Canada	26 weeks	Rosiglitazone+ glyburide vs placebo+ glyburide	0/56 vs 2/58	TZD use: no (Ref)<br>0.21 (0.01, 4.22)	Cancer incidence	high
GSK 49653/136, Europe	26 weeks	Rosiglitazone vs placebo	2/148 vs 0/143	TZD use: no (Ref)<br>4.83 (0.23, 99.79)	Cancer incidence	high
GSK 49653/145	26 weeks	Rosiglitazone+ gliclazide vs gliclazide	1/231 vs 0/242	TZD use: no (Ref)<br>3.14 (0.13, 76.75)	Cancer incidence	high
GSK 49653/147,	26 weeks	Rosiglitazone+ sulphonylurea vs placebo+	0/89 vs 0/88	TZD use: no (Ref)	Cancer	high

UK		sulphonylurea		0.99 (0.02, 49.30)	incidence	
GSK 49653/162, Europe	26 weeks	Rosiglitazone+ glibenclamide vs glibenclamide	2/168 vs 0/172	TZD use: no (Ref)<br>5.12 (0.25, 105.83)	Cancer incidence	high
GSK 49653/234, Germany	26 weeks	Rosiglitazone+ glimepiride vs placebo + glimepiride	1/116 vs 0/58	TZD use: no (Ref)<br>1.51 (0.06, 36.57)	Cancer incidence	high
GSK 49653/390, Nigeria	26 weeks	Rosiglitazone+ sulphonylurea vs sulphonylurea	1/33 vs 0/30	TZD use: no (Ref)<br>2.74 (0.12, 64.69)	Cancer incidence	high
GSK 49653/369, Sweden	6 months	Rosiglitazone vs glibenclamide	0/25 vs 0/24	TZD use: no (Ref)<br>0.96 (0.02, 46.63)<br>Sulfonylurea use: no (Ref)<br>1.04 (0.02, 50.43)	Cancer incidence	high
GSK 49653/132, China	24 weeks	Rosiglitazone+ sulphonylurea vs placebo+ sulphonylurea	1/442 vs 1/112	TZD use: no (Ref)<br>0.25 (0.02, 4.02)	Cancer incidence	high
GSK 49653/347, US	24 weeks	Rosiglitazone+insulin vs placebo+insulin	0/418 vs 1/212	TZD use: no (Ref)<br>0.17 (0.007, 4.14)	Cancer incidence	high
GSK 49653/015, Europe	26 weeks	Rosiglitazone+ sulphonylurea vs placebo+ sulphonylurea	4/395 vs 0/198	TZD use: no (Ref)<br>4.52 (0.24, 83.59)	Cancer incidence	high
GSK 49653/284, US	24 weeks	Rosiglitazone+ metformin vs metformin	1/382 vs 0/384	TZD use: no (Ref)<br>3.02 (0.12, 73.80)	Cancer incidence	high
SB-712753/002	24 weeks	Rosiglitazone+ metformin vs metformin	1/288 vs 0/280	TZD use: no (Ref)<br>2.92 (0.12, 71.31)	Cancer incidence	high
GSK 49653/090, US	8 weeks	Rosiglitazone vs placebo	1/228 vs 0/75	TZD use: no (Ref)<br>1.00 (0.04, 24.19)	Cancer incidence	high
GSK 49653/325, US and Canada	24 weeks	Rosiglitazone vs placebo	0/196 vs 1/195	TZD use: no (Ref)<br>0.33 (0.01, 8.09)	Cancer incidence	high
SB-712753/009	24 weeks	Rosiglitazone+ metformin+insulin vs insulin	2/162 vs 0/160	TZD use: no (Ref)<br>4.94 (0.24, 102.07)<br>Metformin use: no (Ref)<br>4.94 (0.24, 102.07)	Cancer incidence	high
AVD102209, China	24 weeks	Rosiglitazone+insulin vs placebo+insulin	0/132 vs 0/131	TZD use: no (Ref)<br>0.99 (0.02, 49.65)	Cancer incidence	high
GSK 49653/143, US	24 weeks	Rosiglitazone+ glyburide vs glyburide	1/121 vs 0/124	TZD use: no (Ref)<br>3.07 (0.13, 74.73)	Cancer incidence	high
GSK 49653/207, International	24 weeks	Rosiglitazone vs metformin	0/99 vs 0/101	TZD use: no (Ref)<br>1.02 (0.02, 50.91)<br>Metformin use: no (Ref)<br>0.98 (0.02, 48.93)	Cancer incidence	high
GSK 49653/282, US	24 weeks	Rosiglitazone+ metformin vs glyburide + metformin	0/70 vs 0/75	TZD use: no (Ref)<br>1.07 (0.02, 53.23)<br>Sulfonylurea use: no (Ref)<br>0.93 (0.02, 46.46)	Cancer incidence	high
Pfu¨ tzner et al ([100]), international	76 weeks	Saxagliptin+metformin vs saxagliptin vs metformin	1/643 vs 0/335 vs 1/328	DPP-4 inhibitor use: no (Ref)<br>0.34 (0.02, 5.35)<br>Metformin use: no (Ref)	Cancer incidence	high

				1.73 (0.08, 35.91)		
Rosenstock et al (101), US	12 weeks	Saxagliptin vs placebo	10/315 vs 2/108	DPP-4 inhibitor use: no (Ref)<br>1.71 (0.38, 7.70)	Cancer incidence	low
Hollander et al (102), US	24 weeks	Saxagliptin+TZD vs placebo+TZD	4/381 vs 1/184	DPP-4 inhibitor use: no (Ref)<br>1.93 (0.22, 17.16)	Cancer incidence	low
Go¨ ke et al (103), Europe	52 weeks	Saxagliptin vs glipizide	3/428 vs 0/430	DPP-4 inhibitor use: no (Ref)<br>7.03 (0.36, 135.75)<br>Sulfonylurea use: no (Ref)<br>0.14 (0.007, 2.74)	Cancer incidence	low
Raz et al (104), international	44 weeks	Saxagliptin vs placebo	6/411 vs 5/110	DPP-4 inhibitor use: no (Ref)<br>0.32 (0.10, 1.03)	Cancer incidence	high
Dejager et al (105), international	24 weeks	Vildagliptin vs placebo	0/472 vs 1/160	DPP-4 inhibitor use: no (Ref)<br>0.11 (0.005, 2.77)	Cancer incidence	low
Chacra et al (106),international	24 weeks	Saxagliptin+ glyburide vs placebo+glyburide	2/501 vs 1/267	DPP-4 inhibitor use: no (Ref)<br>1.07 (0.10, 11.70)	Cancer incidence	low
Rosenstock et al (107), US and Europe	26 weeks	Alogliptin vs Pioglitazone vs Alogliptin+ Pioglitazone	0/164 vs 1/163 vs 0/327	DPP-4 inhibitor use: no (Ref)<br>0.11 (0.005, 2.71)<br>TZD use: no (Ref)<br>1.01 (0.04, 24.63)	Cancer incidence	high
Rosenstock et al (108), Americas, Europe	24 weeks	Vildagliptin vs Rosiglitazone	5/519 vs 1/267	DPP-4 inhibitor use: no (Ref)<br>2.57 (0.30, 21.91)<br>TZD use: no (Ref)<br>0.39 (0.05, 3.31)	Cancer incidence	high
Bosi et al (109), US and Europe	24 weeks	Vildagliptin vs placebo	0/362 vs 1/182	DPP-4 inhibitor use: no (Ref)<br>0.17 (0.007, 4.11)	Cancer incidence	high
Garber et al (110),Americas, Europe	24 weeks	Vildagliptin+ glimepiride vs placebo+ glimepiride	2/339 vs 0/176	DPP-4 inhibitor use: no (Ref)<br>2.60 (0.13, 53.93)	Cancer incidence	high
Scherbaum et al (111),Europe	52 weeks	Vildagliptin vs placebo	2/156 vs 2/150	DPP-4 inhibitor use: no (Ref)<br>0.96 (0.14, 6.74)	Cancer incidence	high
Ferrannini et al (112), US and Germany	52 weeks	Vildagliptin+metformin vs glimepiride+metformin	9/1396 vs 7/1393	DPP-4 inhibitor use: no (Ref)<br>1.28 (0.48, 3.44)<br>Sulfonylurea use: no (Ref)<br>0.78 (0.29, 2.09)	Cancer incidence	high
Pan et al (113), China, Europe	24 weeks	Vildagliptin vs acarbose	1/441 vs 0/220	DPP-4 inhibitor use: no (Ref)<br>1.50 (0.06, 36.67)<br>Alpha-glucosidase inhibitor use: no (Ref)<br>0.67 (0.03, 16.30)	Cancer incidence	high
DeFronzo et al (114), international	24 weeks	Saxagliptin+metformin vs placebo+ metformin	9/564 vs 2/179	DPP-4 inhibitor use: no (Ref)<br>1.43 (0.31, 6.55)	Cancer incidence	low
Nauck et al (115), international (15 countries)	26 weeks	Alogliptin+ metformin vs placebo+ metformin	2/423 vs 0/104	DPP-4 inhibitor use: no (Ref)<br>1.24 (0.06, 25.60)	Cancer incidence	high

DeFronzo et al ([116]), international	26 weeks	Alogliptin vs placebo	0/264 vs 1/64	DPP-4 inhibitor use: no (Ref)<br>0.08 (0.003, 1.98)	Cancer incidence	high
Pratley et al ([117]), 16 countries	26 weeks	Alogliptin vs placebo	2/401 vs 0/99	DPP-4 inhibitor use: no (Ref)<br>1.24 (0.06, 25.70)	Cancer incidence	low
Pratley et al ([118]), international	26 weeks	Alogliptin+pioglitazone vs placebo+pioglitazone	0/396 vs 2/97	DPP-4 inhibitor use: no (Ref)<br>0.05 (0.002, 1.02)	Cancer incidence	high
NCT00305604, US	24 weeks	Sitagliptin vs placebo	3/102 vs 0/104	DPP-4 inhibitor use: no (Ref)<br>7.14 (0.37, 136.44)	Cancer incidence	high
Bergenstal et al ([119]), US, India, Mexico	26 weeks	Exenatide vs Sitagliptin vs Pioglitazone	0/160 vs 1/166 vs 0/165	GLP-1 agonist use: no (Ref)<br>0.69 (0.03, 16.78)<br>DPP-4 inhibitor use: no (Ref)<br>5.86 (0.24, 142.99)<br>TZD use: no (Ref)<br>0.66 (0.03, 16.03)	Cancer incidence	low
NCT00698932, Asia	24 weeks	Saxagliptin vs placobo	0/284 vs 0/284	DPP-4 inhibitor use: no (Ref)<br>1.00 (0.02, 50.23)	Cancer incidence	high
Arechavaleta et al ([120]), US	30 weeks	Sitagliptin vs Glimepiride	2/516 vs 1/519	DPP-4 inhibitor use: no (Ref)<br>2.01 (0.18, 22.12)<br>Sulfonylurea use: no (Ref)<br>0.50 (0.05, 5.47)	Cancer incidence	low
NCT01028391, US	30 weeks	Sitagliptin+ Pioglitazone vs Pioglitazone	0/164 vs 0/153	DPP-4 inhibitor use: no (Ref)<br>0.93 (0.02, 46.75)	Cancer incidence	high
Chan et al ([121]), international	54 weeks	Sitagliptin vs glipizide	3/65 vs 2/26	DPP-4 inhibitor use: no (Ref)<br>0.60 (0.11, 3.39)<br>Sulfonylurea use: no (Ref)<br>1.67 (0.30, 9.41)	Cancer incidence	low
Pratley et al ([122]), America and Europe	52 weeks	Sitagliptin vs liraglutide	1/219 vs 5/446	DPP-4 inhibitor use: no (Ref)<br>0.41 (0.05, 3.47)<br>GLP-1 agonist use: no (Ref)<br>2.46 (0.29, 20.89)	Cancer incidence	low
4022[123]	48 weeks	Insulin glargine vs oral glucose-lowering drugs	0/118 vs 0/130	Insulin use: no (Ref)<br>1.10 (0.02, 55.05)	Cancer incidence	high
4020[123]	48 weeks	insulin glargine vs Pioglitazone	0/164 vs 0/181	Insulin use: no (Ref)<br>1.10 (0.02, 55.28)<br>TZD use: no (Ref)<br>0.91 (0.02, 45.43)	Cancer incidence	high
Blickle´ et al ([124]), Europe	9 months	insulin glargine vs oral glucose-lowering drugs+dietary	2/103 vs 0/108	Insulin use: no (Ref)<br>5.24 (0.25, 107.87)	Cancer incidence	high
Davies et al ([125]), UK	26 weeks	insulin glargine vs exenatide	0/116 vs 0/118	Insulin use: no (Ref)<br>1.02 (0.02, 50.84)<br>GLP-1 agonist use: no (Ref)<br>0.98 (0.02, 49.14)	Cancer incidence	high
Apovian et al ([126]), US	24 weeks	Exenatide vs placebo	0/96 vs 0/98	GLP-1 agonist use: no (Ref)<br>1.02 (0.02, 50.93)	Cancer incidence	high

Liutkus et al ([127]), international	26 weeks	Exenatide vs placebo	0/111 vs 0/54	GLP-1 agonist use: no (Ref)<br>0.49 (0.01, 24.42)	Cancer incidence	high
Heine et al ([128]), international	26 weeks	Exenatide vs Insulin Glargine	1/282 vs 0/267	GLP-1 agonist use: no (Ref)<br>2.84 (0.12, 69.44)<br>Insulin use: no (Ref)<br>0.35 (0.01, 8.60)	Cancer incidence	high
Nauck et al ([129]),international	52 weeks	Exenatide vs Biphasic Insulin Aspart	0/253 vs 1/248	GLP-1 agonist use: no (Ref)<br>0.33 (0.01, 7.98)<br>Insulin use: no (Ref)<br>3.06 (0.13, 74.77)	Cancer incidence	high
DeFronzo et al ([130]), US	20 weeks	Exenatide vs Exenatide+ Rosiglitazone vs Rosiglitazone	0/45 vs 1/47 vs 0/45	GLP-1 agonist use: no (Ref)<br>1.48 (0.06, 35.72)<br>TZD use: no (Ref)<br>1.48 (0.06, 35.72)	Cancer incidence	high
Gallwitz et al ([131]), Germany	26 weeks	Exenatide vs Premixed Insulin Aspart	2/247 vs 0/233	GLP-1 agonist use: no (Ref)<br>4.72 (0.23, 97.76)<br>Insulin use: no (Ref)<br>0.21 (0.01, 4.39)	Cancer incidence	high
Gill et al ([132]), Canada and the Netherlands	12 weeks	Exenatide vs placebo	0/28 vs 0/26	GLP-1 agonist use: no (Ref)<br>0.93 (0.02, 45.30)	Cancer incidence	high
Kadowaki et al ([133]), Japan	24 weeks	Exenatide vs placebo	1/144 vs 1/35	GLP-1 agonist use: no (Ref)<br>0.24 (0.02, 3.79)	Cancer incidence	high
Buse et al ([134]), international	30 weeks	Exenatide vs placebo	0/137 vs 0/122	GLP-1 agonist use: no (Ref)<br>0.89 (0.02, 44.58)	Cancer incidence	high
Fonseca et al ([135]), US and Europe	24 weeks	Vildagliptin+insulin vs placebo+insulin	1/144 vs 0/152	DPP-4 inhibitor use: no (Ref)<br>3.17 (0.13, 77.09)	Cancer incidence	high
Jin et al ([136]), China	52 weeks	Losartan+pioglitazone vs losartan	0/30 vs 0/30	TZD use: no (Ref)<br>1.00 (0.02, 48.83)	Cancer mortality	high
Derosa et al ([137]), Italy	52 weeks	Pioglitazone vs Acarbose	0/175 vs 0/175	TZD use: no (Ref)<br>1.00 (0.02, 50.12)<br>Alpha-glucosidase inhibitor use: no (Ref)<br>1.00 (0.02, 50.12)	Cancer incidence, same for cancer mortality	high
Derosa et al ([138]), Italy	108 weeks	Pioglitazone vs Acarbose	0/175 vs 0/175	TZD use: no (Ref)<br>1.00 (0.02, 50.12)<br>Alpha-glucosidase inhibitor use: no (Ref)<br>1.00 (0.02, 50.12)	Cancer incidence	high
Nakamura et al ([139]), Japan	52 weeks	Pioglitazone vs voglibose vs glibenclamide	0/15 vs 0/15 vs 0/15	TZD use: no (Ref)<br>1.94 (0.04, 93.19)<br>Alpha-glucosidase inhibitor use: no (Ref)<br>1.94 (0.04, 93.19)	Cancer incidence, same for cancer mortality	high

				Sulfonylurea use: no (Ref)<br>1.94 (0.04, 93.19)		
Heliovaara et al ([140]), Europe	52 weeks	Pioglitazone vs glibenclamide	0/29 vs 0/30	TZD use: no (Ref)<br>1.03 (0.02, 50.43)<br>Sulfonylurea use: no (Ref)<br>0.97 (0.02, 47.23)	Cancer incidence	high
Tan et al ([141]),Europe	52 weeks	Pioglitazone vs glibenclamide	0/91 vs 0/109	TZD use: no (Ref)<br>1.20 (0.02, 59.67)<br>Sulfonylurea use: no (Ref)<br>0.84 (0.02, 41.74)	Cancer incidence	high
Perriello et al ([142]), Italy	52 weeks	Pioglitazone vs gliclazide	0/146 vs 0/137	TZD use: no (Ref)<br>0.94 (0.02, 46.99)<br>Sulfonylurea use: no (Ref)<br>1.07 (0.02, 53.32)	Cancer incidence	high
Matthews et al ([143]), Australia, Europe	52 weeks	Pioglitazone vs gliclazide	0/317 vs 0/313	TZD use: no (Ref)<br>0.99 (0.02, 49.61)<br>Sulfonylurea use: no (Ref)<br>1.01 (0.02, 50.88)	Cancer incidence, same for cancer mortality	high
Yamanouchi et al ([144]), Japan	12 months	Pioglitazone vs Glimepiride vs metformin	0/38 vs 0/37 vs 0/39	TZD use: no (Ref)<br>1.97 (0.04, 97.64)<br>Sulfonylurea use: no (Ref)<br>2.05 (0.04, 101.48)<br>Metformin use: no (Ref)<br>1.90 (0.04, 93.98)	Cancer incidence, same for cancer mortality	high
Derosa et al ([145]), Italy	15 months	Pioglitazone vs metformin vs Pioglitazone+metformin vs Glimepiride+metformin	0/69 vs 0/67 vs 0/69 vs 0/66	TZD use: no (Ref)<br>0.96 (0.02, 48.24)<br>Metformin use: no (Ref)<br>0.34 (0.007, 17.22)<br>Sulfonylurea use: no (Ref)<br>3.07 (0.06, 153.47)	Cancer incidence, same for cancer mortality	high
Tan et al ([146]), Mexico	52 weeks	Pioglitazone vs Glimepiride	0/121 vs 0/123	TZD use: no (Ref)<br>1.02 (0.02, 50.82)<br>Sulfonylurea use: no (Ref)<br>0.98 (0.02, 49.19)	Cancer incidence	high
Abe et al ([147]), Japan	96 weeks	Pioglitazone vs control	0/31 vs 0/32	TZD use: no (Ref)<br>1.03 (0.02, 50.43)	Cancer incidence, same for cancer mortality	high
NCT00386100, Brazil, Taiwan	80 weeks	Rosiglitazone+ metformin vs metformin	1/344 vs 1/334	TZD use: no (Ref)<br>0.97 (0.06, 15.46)	Cancer incidence	high
Dargie et al ([148]), Europe	52 weeks	Rosiglitazone vs placebo	2/110 vs 3/114 (incidence)<br>1/110 vs 0/114 (mortality)	TZD use: no (Ref)<br>0.69 (0.12, 4.06) (incidence)<br>3.11 (0.13, 75.49) (mortality)	Cancer incidence, cancer mortality	high

Hedblad et al ([149]), Sweden	52 weeks	Rosiglitazone vs placebo	3/277 vs 3/278 (incidence)<br>3/277 vs 1/278 (mortality)	TZD use: no (Ref)<br>1.00 (0.20, 4.93) (incidence)<br>3.01 (0.32, 28.77) (mortality)	Cancer incidence, cancer mortality	high
Berberoglu et al ([150]), Turkey	104 weeks	Rosiglitazone vs diet	0/26 vs 0/23	TZD use: no (Ref)<br>0.89 (0.02, 43.10)	Cancer incidence, same for cancer mortality	high
Rosenstock et al ([151]), US and Canada	104 weeks	Rosiglitazone+ glipizide vs placebo+ glipizide	4/116 vs7/111(incidence)<br>0/116 vs 0/111(mortality)	TZD use: no (Ref)<br>0.55 (0.16, 1.82) (incidence)<br>0.96 (0.02, 47.83) (mortality)	Cancer incidence, cancer mortality	high
Gerstein et al ([152]), international	18 months	Rosiglitazone vs glipizide	1/333 vs 4/339	TZD use: no (Ref)<br>0.25 (0.03, 2.27)<br>Sulfonylurea use: no (Ref)<br>3.93 (0.44, 34.97)	Cancer incidence	high
Gram et al ([153]), Denmark	104 weeks	Rosiglitazone vs placebo; metformin vs placebo	3/187 vs 8/184; 6/184 vs 5/187	TZD use: no (Ref)<br>0.37 (0.10, 1.37)<br>metformin use: no (Ref)<br>1.22 (0.38, 3.93)	Cancer incidence	high
Bertrand et al ([154]), Canada	52 weeks	Rosiglitazone vs placebo	0/98 vs 0/95	TZD use: no (Ref)<br>0.97 (0.02, 48.38)	cancer mortality	high
Mazzone et al ([155]), US	72 weeks	Pioglitazone vs Glimepiride	1/230 vs 0/228	TZD use: no (Ref)<br>2.97 (0.12, 72.63)<br>Sulfonylurea use: no (Ref)<br>0.34 (0.01, 8.21)	cancer mortality	high
Nissen et al ([156]), America	78 weeks	Pioglitazone vs Glimepiride	3/270 vs 4/273	TZD use: no (Ref)<br>0.76 (0.17, 3.36)<br>Sulfonylurea use: no (Ref)<br>1.32 (0.30, 5.84)	Cancer incidence	low
Tolman et al ([157]), US	3 years	Pioglitazone vs glibenclamide	0/1063 vs 0/1057	TZD use: no (Ref)<br>0.99 (0.02, 50.07)<br>Sulfonylurea use: no (Ref)<br>1.01 (0.02, 50.64)	cancer mortality	low
Giles et al ([158]), US	52 weeks	Pioglitazone vs glyburide	0/151 vs 0/149	TZD use: no (Ref)<br>0.99 (0.02, 49.42)<br>Sulfonylurea use: no (Ref)<br>1.01 (0.02, 50.74)	cancer mortality	high
Owens et al ([159]), international	24 weeks	Linagliptin vs placebo	0/792 vs 0/263	DPP-4 inhibitor use: no (Ref)<br>0.33 (0.007, 16.74)	Cancer incidence	high
Gomis et al ([160]), international	24 weeks	Linagliptin+ Pioglitazone vs placebo+ Pioglitazone	0/259 vs 0/130	DPP-4 inhibitor use: no (Ref)<br>0.50 (0.01, 25.25)	Cancer incidence	high
Kawamori et al ([161]), Japan	26 weeks	Linagliptin vs voglibose vs placebo	6/319 vs 1/162 vs 0/80	DPP-4 inhibitor use: no (Ref)<br>4.55 (0.55, 37.56)	Cancer incidence	low

				Alpha-glucosidase inhibitor use: no (Ref)<br>0.41 (0.05, 3.38)		
Haak et al ([162]), 14 countries	24 weeks	Placebo vs metformin vs Linagliptin vs Linagliptin+metformin	0/72 vs 1/291 vs 1/142 vs 1/286	DPP-4 inhibitor use: no (Ref)<br>1.70 (0.15, 18.63)<br>Metformin use: no (Ref)<br>0.74 (0.07, 8.14)	Cancer incidence	high
Lewin et al ([163]), international	18 weeks	Linagliptin vs placebo	0/161 vs 0/84	DPP-4 inhibitor use: no (Ref)<br>0.52 (0.01, 26.21)	Cancer incidence	high
Nauck et al ([164]), US, Germany	52 weeks	Sitagliptin vs Glipizide	10/588 vs 14/584	DPP-4 inhibitor use: no (Ref)<br>0.71 (0.32, 1.58)<br>Sulfonylurea use: no (Ref)<br>1.41 (0.63, 3.15)	Cancer incidence	high
Mohan et al ([165]), Asia	18 weeks	Sitagliptin vs placebo	2/352 vs 0/178	DPP-4 inhibitor use: no (Ref)<br>2.54 (0.12, 52.53)	Cancer incidence	low
Dobs et al ([166]), international	54 weeks	Sitagliptin vs placebo	3/170 vs 2/92	DPP-4 inhibitor use: no (Ref)<br>0.81 (0.14, 4.77)	Cancer incidence	low
Olansky et al ([167]), US	44 weeks	Sitagliptin/Metformin vs metformin	3/625 vs 5/621	DPP-4 inhibitor use: no (Ref)<br>0.60 (0.14, 2.48)	Cancer incidence	high
Alba et al ([168]), US, Sweden	12 weeks	Sitagliptin vs Pioglitazone vs Sitagliptin+ Pioglitazone vs placebo	1/52 vs 0/54 vs 0/52 vs 0/53	DPP-4 inhibitor use: no (Ref)<br>3.09 (0.13, 74.90)<br>TZD use: no (Ref)<br>0.33 (0.01, 8.02)	Cancer incidence	high
Scott et al ([169]), New Zealand, US	18 weeks	Sitagliptin vs Rosiglitazone vs placebo	0/94 vs 0/87 vs 1/91	DPP-4 inhibitor use: no (Ref)<br>0.63 (0.03, 15.27)<br>TZD use: no (Ref)<br>0.70 (0.03, 17.12)	Cancer incidence	high
Rahman et al ([170]),Malaysia	52 weeks	Rosiglitazone vs placebo	0/11 vs 0/11	TZD use: no (Ref)<br>1.00 (0.02, 46.41)	Cancer incidence	low
Schweizer et al ([171]), America, Europe	52 weeks	Vildagliptin vs Metformin	3/526 vs 3/254	DPP-4 inhibitor use: no (Ref)<br>0.48 (0.10, 2.38)<br>Metformin use: no (Ref)<br>2.07 (0.42, 10.19)	Cancer incidence	high
NCT00283049, US	12 weeks	Insulin Glargine + Sulfonylurea+ Thiazolidinedione vs Insulin Glargine + Metformin + Thiazolidinedione vs Insulin Glargine + Metformin + Sulfonylurea	3/128 vs 2/128 vs 1/130	Sulfonylurea use: no (Ref)<br>0.99 (0.18, 5.35)<br>TZD use: no (Ref)<br>2.54 (0.30, 21.51)<br>Metformin use: no (Ref)<br>0.50 (0.10, 2.42)	Cancer incidence	high
Kahn et al ([172]), America, Europe	Median 4 years	Metformin vs rosiglitazone vs glyburide	50/1454 vs 55/1456 vs 55/1441	Metformin use: no (Ref)<br>0.91 (0.65, 1.26)<br>TZD use: no (Ref)<br>1.04 (0.76, 1.43)<br>Sulfonylurea use: no (Ref)	Cancer incidence	low

				1.06 (0.77, 1.46)		
Rosenstock et al (173), US	24 weeks	Sitagliptin vs placebo	1/175 vs 0/178	DPP-4 inhibitor use: no (Ref)<br>3.05 (0.13, 74.40)	Cancer incidence	high
Aschner et al (174), US	24 weeks	Sitagliptin vs placebo	9/488 vs 5/253	DPP-4 inhibitor use: no (Ref)<br>0.93 (0.32, 2.76)	Cancer incidence	high
Raz et al (175), Israel, US	30 weeks	Sitagliptin vs placebo	0/96 vs 3/94	DPP-4 inhibitor use: no (Ref)<br>0.14 (0.007, 2.67)	Cancer incidence	low
Visbøll et al (176), international	24 weeks	Sitagliptin vs placebo	2/322 vs 0/319	DPP-4 inhibitor use: no (Ref)<br>4.95 (0.24, 102.78)	Cancer incidence	low
Yoon et al (177), international	24 weeks	Sitagliptin+Pioglitazone vs Pioglitazone	0/261 vs 0/259	DPP-4 inhibitor use: no (Ref)<br>0.99 (0.02, 49.83)	Cancer incidence	low
Charbonnel et al (178), international	24 weeks	Sitagliptin vs placebo	11/464 vs 1/237	DPP-4 inhibitor use: no (Ref)<br>5.62 (0.73, 43.26)	Cancer incidence	high
Hermansen et al (179), international	24 weeks	Sitagliptin vs placebo	3/222 vs 1/219	DPP-4 inhibitor use: no (Ref)<br>2.96 (0.31, 28.23)	Cancer incidence	high
Bolli et al (180), international	52 weeks	Vildagliptin vs Pioglitazone	1/295 vs 5/281	DPP-4 inhibitor use: no (Ref)<br>0.19 (0.02, 1.62)<br>TZD use: no (Ref)<br>5.25 (0.62, 44.65)	Cancer incidence	low
Del Prato et al (181), international	24 weeks	Linagliptin vs Placebo	1/336 vs 2/167	DPP-4 inhibitor use: no (Ref)<br>0.25 (0.02, 2.72)	Cancer incidence	high
Taskinen et al (182),international	24 weeks	Linagliptin vs Placebo	0/523 vs 0/177	DPP-4 inhibitor use: no (Ref)<br>0.34 (0.007, 17.06)	Cancer incidence	high
Aschner et al (183),international	24 weeks	Sitagliptin vs metformin	1/528 vs 1/522	DPP-4 inhibitor use: no (Ref)<br>0.99 (0.06, 15.76)<br>Metformin use: no (Ref)<br>1.01 (0.06, 16.13)	Cancer incidence	high
Home et al (184), Europe and Australasia	5.5 years	Sulfonylurea+metformin vs Sulfonylurea+ Rosiglitazone vs metformin+ Rosiglitazone	143/2227 vs 56/1103 vs 57/1117	Sulfonylurea use: no (Ref)<br>1.17 (0.88, 1.56)<br>Metformin use: no (Ref)<br>1.18 (0.88, 1.57)<br>TZD use: no (Ref)<br>0.79 (0.62, 1.01)	Cancer incidence	high
Hanefeld et al (185), Europe, Canada	52 weeks	Sulfonylurea+ pioglitazone vs Sulfonylurea+metformin	6/319 vs 3/320	TZD use: no (Ref)<br>2.01 (0.51, 7.95)<br>Metformin use: no (Ref)<br>0.50 (0.13, 1.98)	Cancer incidence	high
Schernthaner et al (186), Europe	52 weeks	pioglitazone vs metformin	6/597 vs 3/597	TZD use: no (Ref)<br>2.00 (0.50, 7.96)<br>Metformin use: no (Ref)<br>0.50 (0.13, 1.99)	Cancer incidence	high
Cryer et al (187), US	1 year	Metformin vs others	92/7227 vs 20/1505	Metformin use: no (Ref)<br>0.96 (0.59, 1.55)	Cancer incidence	high
Williams-Herman	104 weeks	Sitagliptin vs Metformin vs metformin+	3/179 vs 9/540 vs 4/489	DPP-4 inhibitor use: no (Ref)	Cancer	low

et al ([188]), international		Sitagliptin		0.63 (0.24, 1.68)<br>Metformin use: no (Ref)<br>0.75 (0.22, 2.62)	incidence	
Turner et al ([189]), UK	Median 10.7 years	Metformin vs diet	13/342 vs 21/411	Metformin use: no (Ref)<br>0.74 (0.38, 1.46)	Cancer mortality	high
Turner et al ([189]), UK	Median 10.7 years	Metformin+ Sulfonylurea vs Sulfonylurea	14/268 vs 6/269	Metformin use: no (Ref)<br>2.34 (0.91, 6.00)	Cancer mortality	high
Ferrannini et al ([190]), international	24 weeks	Dapagliflozin vs placebo	5/410 vs 1/75	Dapagliflozin use: no (Ref)<br>0.91 (0.11, 7.72)	Cancer incidence	high
Bailey et al ([191]), America	24 weeks	Dapagliflozin+metformin vs placebo+metformin	5/409 vs 3/137	Dapagliflozin use: no (Ref)<br>0.56 (0.14, 2.31)	Cancer incidence	low
Nauck et al ([192]), international	52 weeks	Dapagliflozin+metformin vs Glipizide +metformin	4/406 vs 3/408	Dapagliflozin use: no (Ref)<br>1.34 (0.30, 5.95)<br>Sulfonylurea use: no (Ref)<br>0.75 (0.17, 3.31)	Cancer incidence	low
Jain et al ([193]), US, Puerto Rico	56 weeks	Glyburide vs Pioglitazone	2/251 vs 0/251	Sulfonylurea use: no (Ref)<br>5.00 (0.24, 103.63)<br>TZD use: no (Ref)<br>0.20 (0.01, 4.15)	Cancer incidence	high
Derumeaux et al ([194]), international	1 year	Benfluorex vs Pioglitazone	3/421 vs 2/423	TZD use: no (Ref)<br>0.66 (0.11, 3.95)	Cancer incidence	low
Kikuchi et al ([195]), Japan	12 weeks	Vildagliptin vs placebo	0/219 vs 1/72	DPP-4 inhibitor use: no (Ref)<br>0.11 (0.005, 2.69)	Cancer incidence	high
Kawamori et al ([196]), Japan, Korea	16 weeks	insulin glulisine+ sulphonylurea vs insulin glulisine vs sulphonylurea	0/130 vs 1/127 vs 1/130	Insulin use: no (Ref)<br>0.51 (0.03, 8.02)<br>Sulfonylurea use: no (Ref)<br>0.49 (0.03, 7.75)	Cancer incidence	high
Kaku et al ([197]), Japan	12 weeks	TAK-875 vs glimepiride vs placebo	1/299 vs 0/49 vs 0/48	TAK-875 use: no (Ref)<br>0.98 (0.04, 23.86)<br>Sulfonylurea use: no (Ref)<br>2.32 (0.10, 56.18)	Cancer incidence	high
Kendall et al ([198]), US	50 weeks	Muraglitazar vs Pioglitazone	1/587 vs 0/572	TZD use: no (Ref)<br>0.34 (0.01, 8.38)	Cancer mortality	high
Seino et al ([199]), Japan	14 weeks	Liraglutide vs placebo	1/180 vs 0/46	GLP-1 agonist use: no (Ref)<br>0.78 (0.03, 18.82)	Cancer incidence	low
Kadowaki et al ([200]), Japan	12 weeks	Sitagliptin vs placebo	0/77 vs 0/72	DPP-4 inhibitor use: no (Ref)<br>0.94 (0.02, 46.56)	Cancer incidence	high
Gallwitz et al ([201]), international	2 years	Linagliptin vs glimepiride	23/776 vs 25/775	DPP-4 inhibitor use: no (Ref)<br>0.92 (0.53, 1.60)<br>Sulfonylurea use: no (Ref)<br>1.09 (0.62, 1.90)	Cancer incidence	low
Diamant et al ([202]), Europe, US	84 weeks	Exenatide vs Insulin Glargine	1/233 vs 1/223	GLP-1 agonist use: no (Ref)<br>0.96 (0.06, 15.21)<br>Insulin use: no (Ref)	Cancer incidence	high

				1.04 (0.07, 16.60)		
Wilding et al (203), international	104 weeks	Dapagliflozin vs placebo	15/610 vs 6/197	Dapagliflozin use: no (Ref)<br>0.81 (0.32, 2.05)	Cancer incidence	low
Strojek et al (204), international	24 weeks	Dapagliflozin+ Glimepiride vs placebo+Glimepiride	3/450 vs 1/146	Dapagliflozin use: no (Ref)<br>0.97 (0.10, 9.29)	Cancer incidence	low
Rosenstock et al (205), international	24 weeks	Dapagliflozin+ pioglitazone vs placebo+ pioglitazone	1/281 vs 0/139	Dapagliflozin use: no (Ref)<br>1.49 (0.06, 36.33)	Cancer incidence	high
Barnett et al (206), international	1 year	Linagliptin vs Placebo/Glimepiride	0/151 vs 1/76	DPP-4 inhibitor use: no (Ref)<br>0.17 (0.007, 4.10)<br>Sulfonylurea use: no (Ref)<br>5.92 (0.24, 143.68)	Cancer incidence	high
Aschner et al (207), international	24 weeks	Sitagliptin vs Insulin Glargine	2/264 vs 0/237	DPP-4 inhibitor use: no (Ref)<br>4.49 (0.22, 93.07)<br>Insulin use: no (Ref)<br>0.22 (0.01, 4.62)	Cancer incidence	high
Bolinder et al (208), Europe	102 weeks	Dapagliflozin+metformin vs placebo+ metformin	1/91 vs 0/91	Dapagliflozin use: no (Ref)<br>3.00 (0.12, 72.69)	Cancer incidence	low
White et al (209), US and Europe	Median 18 months	Alogliptin vs placebo	50/2701 vs 30/2679	DPP-4 inhibitor use: no (Ref)<br>1.65 (1.05, 2.59)	Cancer incidence	low
Scirica et al (210), international	Median 2.1 years	Saxagliptin vs placebo	327/8280 vs 362/8212	DPP-4 inhibitor use: no (Ref)<br>0.90 (0.77, 1.04)	Cancer incidence	low
Wang et al (211), Taiwan	16 weeks	Metformin+Acarbose vs Acarbose	0/117 vs 2/116	Metformin use: no (Ref)<br>0.20 (0.01, 4.09)	Cancer incidence	high
Seino et al (212), Japan	64 weeks	Alogliptin+ Glimepiride vs Glimepiride	2/209 vs 0/103	DPP-4 inhibitor use: no (Ref)<br>2.48 (0.12, 51.11)	Cancer incidence	high
Lund et al (213), Denmark	4 months	Repaglinide vs metformin	0/48 vs 2/48	glinides use: no (Ref)<br>0.20 (0.01, 4.06)<br>Metformin use: no (Ref)<br>5.00 (0.25, 101.49)	Cancer incidence	high
Erdmann et al (214), Europe	Mean 8.7 years	Pioglitazone vs placebo	257/2605 vs 247/2633	Pioglitazone use: no (Ref)<br>1.05 (0.89, 1.24)	Cancer incidence	low

numbered as supplementary references
